# A Comprehensive Transcriptome-Wide Identification and Screening of *WRKY* Gene Family Engaged in Abiotic Stress *in Glycyrrhiza glabra*

**DOI:** 10.1038/s41598-019-57232-x

**Published:** 2020-01-15

**Authors:** Pooja Goyal, Malik Muzafar Manzoor, Ram A. Vishwakarma, Deepak Sharma, Manoj K. Dhar, Suphla Gupta

**Affiliations:** 1 Plant Biotechnology Department, Council for Scientific Research-Indian Institute of Integrative Medicine, Canal Road, Jammu, India; 2 Genome Research Laboratory, School of Biotechnology, University of Jammu, Jammu, India

**Keywords:** Plant molecular biology, Abiotic

## Abstract

The study reports 147 full-length *WRKY* genes based on the transcriptome analysis of Glycyrrhiza genus (*G. glabra* and *G. uralensis*). Additional motifs in *G. glabra* included DivIVA (GgWRKY20) and SerS Superfamily (GgWRKY21) at the C-terminal, and Coat family motifs (GgWRKY55) at the N-terminal of the proteins, while Exo70 exo cyst complex subunit of 338 amino acid (GuWRKY9) was present at the N-terminal of *G. uralensis* only. Plant Zn cluster super-family domain (17 WRKYs) and bZIP domain (2 WRKYs) were common between the two species. Based on the number of WRKY domains, sequence alignment and phylogenesis, the study identified GuWRKY27 comprising of 3 WRKY domains in *G. uralensis* and a new subgroup-IIf (10 members), having novel zinc finger pattern (C-X_4_-C-X_22_-HXH) in *G. glabra*. Multiple WRKY binding domains (1–11) were identified in the promoter regions of the *GgWRKY* genes indicating strong interacting network between the WRKY proteins. Tissue-specific expression of 25 *GgWRKYs*, under normal and treated conditions, revealed 11 of the 18 induction factor triggered response corroborating to response observed in *AtWRKYs*. The study identified auxin-responsive *GgWRKY* 55 & *GgWRKY*38; GA_3_ responsive *GgWRKYs*15&59 in roots and *GgWRKYs*8, 20, 38, 57 &58 in the shoots of the treated plant. *GgWRKYs* induced under various stresses included *GgWRKY*33 (cold), *GgWRKY*4 (senescence), *GgWRKYs*2, 28 & 33 (salinity) and *GgWRKY*40 (wounding). Overall, 23 *GgWRKYs* responded to abiotic stress, and 17 *WRKYs* were induced by hormonal signals. Of them 13 *WRKYs* responded to both suggesting inter-connection between hormone signalling and stress response. The present study will help in understanding the transcriptional reprogramming, protein-protein interaction and cross-regulation during stress and other physiological processes in the plant.

## Introduction

The complexity in plant cell organization can be directly related to intricate inter-connections between genes and regulatory network inside the cell. This observation is further substantiated by studies on vast genomic and transcriptomic sequence information available in the public domain^1^. The inter-cellular biological circuit in higher plants is governed at several discreet levels, one of them is regulated by a specific group of DNA binding proteins, the *WRKYs*^2^ are among the ten largest families of transcription factors (TFs) in higher plants. The literature cites several papers after the first report on *Ipomea batata* (SPF1) in 1994^3^, from dicots^4^, monocots^5^, orchids^6^ to unicellular eukaryote (*Giardia lamblia*) and the slime mold (*Dictyostelium discoideum*), revealing their evolutionary significance and complex organization^7,8^. The 60 amino-acid characteristic conserved sequence of WRKY transcription factor (TFs) are most commonly identified by specific hepta-nucleotide signature sequence (WRKYGQK), the W-Box, which binds to the promoter sequence of target gene(s) modulating its activity^9^. The large WRKY super-family is phylogenetically classified into three groups (I, II &III) based on the number of WRKY domains and type of zinc finger sequences at the C-terminal. WRKY proteins classified in group I is characterized by two WRKY domains and zinc-finger motifs (C_2_H_2_), while group II and III WRKY proteins constitute single WRKY domain. Zinc-finger motif in group II & III comprise of C_2_H_2_ and C_2_HC zinc-finger pattern, respectively^10^. Studies have shown WRKY binding motifs (W-boxes) are present in multiple numbers in WRKY responsive gene promoters^11^. The promoters of 83% genes of the 72 *WRKYs* in *Arabidopsis*, contain at least two perfect W-boxes (TTGACC/T), and 58% had four or more core element sequence (TTGAC)^11^. Some *WRKYs* had 11 to 12 (*AtWRKY*66, *AtWRKY*17) core elements in the promoter fragment as analysed by Dong *et al*.^12^. Interestingly, studies confirm the presence of W-boxes also in the promoter region of *WRKY* genes, suggesting a potentially strong transcriptional networking between WRKY proteins^11^. Studies using co-transfection assays have revealed role of WRKY proteins on the promoters of their own genes and on other *WRKY* genes thereby modulating reporter gene^13^. Also *in-vitro* DNA-protein binding assays have highlighted single WRKY binding to several target gene promoters as elucidated in WRKY53 binding to three different *WRKY* genes, confirming complex interactive regulatory network. Microarray experiments using *Arabidopsis* genome illustrated more than 70% (45 out of 61) of the *WRKY* genes are co-regulated with other *WRKY*s^14^ and transcription factors^12^. Biological role of WRKYs are being studied in several plants^15^. They have been found to regulate several target genes in response to stress^16^ including metal stress^17^, development^18^ and secondary metabolite biosynthesis^1^. WRKYs have shown regulatory role in pathogen-induced response^12^ resulting in concerted activation of variety of genes. WRKY TFs have been found to rapidly and transiently regulate gene induction in response to signalling molecule^19^, wounding, stress, physiological processes like flowering^20^, seed germination and development^21^ and senescence^4^. Expressed Sequence Tags (ESTs) and other plant database have revealed presence of several hundred *WRKYs* in various tissues under different physiology, stress^18^, cold^22^, stomatal movement^23^ and defense^24,25^ implying their predominant role in varied biological functions. However, under normal growth conditions also, WRKY proteins have demonstrated broad-spectrum regulatory role as reported in morphogenesis and development of trichomes^26^ embryo development^18^, senescence^13^, dormancy^27^, plant growth^28^, immunity^29^, systematic acquired resistant and metabolic pathways^30^.

Two decades of studies on *WRKY* TFs has resulted in more than 14500 *WRKY* genes from 165 plant species^31^ with most of the species from eudicots (100 species) followed by monocots (38 species) and chlorophytae (16 species)^31^. Legumes with 12 species contributed to 1094 *WRKY* genes^32^. No report on *WRKY* transcription factors has been published from Glycyrrhiza species, though transcriptome, genome and EST databases are available in public domain from *G. uralensis*.

Glycyrrhiza belongs to Fabaceae sub-family of Leguminoseae family. The underground roots (Licorice) of the genus (*G. uralensis, G. glabra and G. echinata*) are commercially valued for its pharmaceutical, flavour enhancer natural sweetener, and cosmaceutical properties^33^. Roots of the plant are rich in bioactive flavonoids and tri-terpenoid saponins including glycyrrhizin^34^. Glycyrrhizin molecule is pharmaceutically sought molecule for its multitude of bioactivities^33^. The global demand of the roots of Glycyrrhiza is evident by a market report, as per Transparency Market report (ALBANY, New York, April 4, 2017 /PRNewswire). Where projected compound annual growth rate was estimated to be 5.7% during 2017–2025 equivalent to USD 2,393.9 million by 2025.

Present research underlines the transcriptome-wide identification and characterization of 147 WRKY TFs from Glycyrrhiza genus. Here, we analysed 87 *WRKY* genes from *G. glabra* and 60 from *G. uralensis*, categorized them into different structural groups based on conserved motif composition. We also predicted functions based on STRING prediction algorithm in *G. glabra* WRKY members. Subsequently their expression profiles were investigated under various stress conditions in the aerial tissues of the *in vitro* cultured plant. We also characterized 31 promoters (between 0.5 kb to 4.1 kb) of the 87 Gg*WRKY* genes (from the transcriptomic data) to get an insight into its functioning and regulation of secondary metabolites.

## Results and Discussion

### Transcriptome-wide analysis and characterization of *Glycyrrhiza* WRKY TF

We have done the transcriptomics of G. *glabra* plant and mined the data for the WRKY transcription factor. Among the 125 sequences that matched *WRKY* genes on BLAST and PF03106 HMM profile searches, 87 Gg*WRKY*s had complete CDS, and 38 gene sequences were partial (Table 1). All of these were revalidated using Uniprot (https://www.uniprot.org/) resulting in 78 sequences with best hits, while 47 sequences were found unique. Out of these, 55 (UniProt hits) and 32 (unique) sequences were full length, and 23 (UniProt hits) and 15 (unique) were partial sequences (Table 1). Further, we used the publicly available *G. uralensis* transcriptome data as a reference source (http://ngs-data-archive.psc.riken.jp/Gur-genome/download.pl.) to retrieve the WRKY transcription factor using BLAST and PF03106 HMM profile searches, we could identify 60 WRKY genes from *G. uralensis*. Subsequently, all the full-length protein sequences (147) were re-examined for the presence of WRKY domains using conserved domain database (https://www.ncbi.nlm.nih.gov/cdd/) and through HMMScan (https://www.ebi.ac.uk/Tools/hmmer/search/hmmscan). The *GgWRKY* sequences were submitted to NCBI, and their accession numbers are given in (Supplementary File 1). The identified GuWRKY protein sequences were included in the sequence alignment and phylogenetic studies only (Supplementary File 2). The detailed GgWRKY protein sequence features are listed in Table 2. The deduced GgWRKY proteins had amino acid residues between 112 (GgWRKY67) to 760 (GgWRKY12). The coding sequences of 87 full-length *GgWRKY*s ranged from 339 bp (GgWRKY67) to 2283 bp (GgWRKY12), and their molecular weight (MW) varied between 13291.91 Da (GgWRKY67) to 82181.16 Da (GgWRKY12) (Table 3). The isoelectric point (pI) of 44 *Gg*WRKYs were acidic, one (GgWRKY55) was neutral with pI value equal to 7.0, and the remaining 42 were basic proteins. According to the instability index proteins with index value higher than 40.0 is unstable^35^. In the present study most of the GgWRKYs were found to be unstable, having maximum instability index of 68.68 (GgWRKY34) with the exception of ten GgWRKYs namely, GgWRKY10 (30.20), GgWRKY16 (38.82), GgWRKY48 (39.86), GgWRKY50 (33.92), GgWRKY60 (39.40), GgWRKY73 (33.70), GgWRKY80 (39.40), GgWRKY83 (37.16), GgWRKY84 (32), GgWRKY86 (35.94) (Table 3). Additionally, the WoLFPSORT prediction showed that 81 GgWRKY proteins were localised in nucleus, suggesting that they play regulatory role predominantly in cell nucleus, while 4 GgWRKYs (-23, 32, 75, 84) had chloroplast orientation. GgWRKY73 had mitochondrial and GgWRKY 86 had cytoplasmic subcellular localization (Table 3). Further, five GgWRKY members (GgWRKYs 10,-33,-67,-68,-87) had WRKYGKK domain instead of the common WRKYGQK (Table 2). Earlier studies have also reported replacement of Q by K as common variant. Rice WRKYs have shown 19 variants, where the characteristic WRKY is substitution by WRRY,WSKY,WKKY, WVKY or WKKY motifs^5^.

**Table 1:** Sequence information of WRKY genes in *G. glabra*.

Sequence type	Uniprot matches	Unique	Total
Full length sequences	55	32	87
Partial sequences	23	15	38
Total	78	47	125

**Table 2:** Sequence features of WRKY genes in *G. glabra.*

GgWRKYs	Gene ID	CDS (bp)	ORF (aa)	group	Conserved motif	Domain pattern	Zinc finger
GgWRKY1	MK511239	1563	520	1	2(WRKYGQK)	C-X4-C-X22-HNH	C2H2
GgWRKY2	MK511240	1155	384	1	2(WRKYGQK)	C-X4-C-X22-HNH	C2H2
GgWRKY3	MK511241	804	267	2e	WRKYGQK	C-X5-C-X23-HNH	C2H2
GgWRKY4	MK511242	840	279	2c	WRKYGQK	C-X4-C-X23-HNH	C2H2
GgWRKY5	MK511243	762	253	1	2(WRKYGQK)	C-X4-C-X22-HNH	C2H2
GgWRKY6	MK511244	603	200	2f	WRKYGQK	C-X4-C-X22-HNH	C2H2
GgWRKY7	MK511245	1767	588	1	2(WRKYGQK)	C-X4-C-X22-HNH	C2H2
GgWRKY8	MK511246	1128	375	1	2(WRKYGQK)	C-X4-C-X22-HNH	C2H2
GgWRKY9	MK511247	1440	479	1	2(WRKYGQK)	C-X4-C-X22-HNH	C2H2
GgWRKY10	MK511248	507	168	2c	WRKYGKK	C-X4-C-X23-HXH	C2H2
GgWRKY11	MK511249	1104	367	2c	WRKYGQK	C-X4-C-X23-HNH	C2H2
GgWRKY12	MK511250	2283	760	1	2(WRKYGQK)	C-X4-C-X23-HNH	C2H2
GgWRKY13	MK511251	1104	367	2c	WRKYGQK	C-X4-C-X23-HNH	C2H2
GgWRKY14	MK511252	1266	421	2f	WRKYGQK	C-X4-C-X22-HXH	C2H2
GgWRKY15	MK511253	1305	434	1	2(WRKYGQK)	C-X4-C-X22-HNH	C2H2
GgWRKY16	MK511254	1206	401	1	2(WRKYGQK)	C-X4-C-X23-HNH	C2H2
GgWRKY17	MK511255	669	222	2f	WRKYGQK	C-X4-C-X22-HXH	C2H2
GgWRKY18	MK511256	1608	535	1	2(WRKYGQK)	C-X4-C-X22-HNH	C2H2
GgWRKY19	MK511257	1527	507	1	2(WRKYGQK)	C-X4-C-X23-HXH	C2H2
GgWRKY20	MK511258	1974	657	2b	WRKYGQK	C-X5-C-X23-HXH	C2H2
GgWRKY21	MK511259	1743	580	2b	WRKYGQK	C-X5-C-X23-HNH	C2H2
GgWRKY22	MK511260	1035	344	1	2(WRKYGQK)	C-X4-C-X22-HXH	C2H2
GgWRKY23	MK511261	723	240	2c	WRKYGQK	C-X4-C-X23-HXH	C2H2
GgWRKY24	MK511262	777	258	2e	WRKYGQK	C-X5-C-X23-HNH	C2H2
GgWRKY25	MK511263	624	207	2e	WRKYGQK	C-X5-C-X23-HNH	C2H2
GgWRKY26	MK511264	624	207	2e	WRKYGQK	C-X5-C-X23-HNH	C2H2
GgWRKY27	MK511265	1617	538	2e	WRKYGQK	C-X5-C-X23-HNH	C2H2
GgWRKY28	MK511266	612	203	2f	WRKYGQK	C-X4-C-X22-HXH	C2H2
GgWRKY29	MK511267	1143	380	2e	WRKYGQK	C-X5-C-X23-HNH	C2H2
GgWRKY30	MK511268	1068	355	3	WRKYGQK	C-X7-C-X23-HXC	C2HC
GgWRKY31	MK511269	1437	478	1	2(WRKYGQK)	C-X4-C-X22-HXH	C2H2
GgWRKY32	MK511270	495	165	3	WRKYGQK	C-X7-C-X23-HXC	C2HC
GgWRKY33	MK511271	498	165	2c	WRKYGKK	C-X4-C-X23-HXH	C2H2
GgWRKY34	MK511272	966	321	3	WRKYGQK	C-X7-C-X23-HXC	C2HC
GgWRKY35	MK511273	1029	342	2c	WRKYGQK	C-X4-C-X23-HNH	C2H2
GgWRKY36	MK511274	579	192	2c	WRKYGQK	C-X4-C-X23-HNH	C2H2
GgWRKY37	MK511275	717	238	2a	WRKYGQK	C-X5-C-X23-HNH	C2H2
GgWRKY38	MK511276	1158	385	2b	WRKYGQK	C-X5-C-X23-HNH	C2H2
GgWRKY39	MK511277	1881	626	2b	WRKYGQK	C-X5-C-X23-HNH	C2H2
GgWRKY40	MK511278	789	262	2a	WRKYGQK	C-X5-C-X23-HNH	C2H2
GgWRKY41	MK511279	1248	415	1	2(WRKYGQK)	C-X4-C-X22-HXH	C2H2
GgWRKY42	MK511280	1029	342	2f	WRKYGQK	C-X4-C-X22-HXH	C2H2
GgWRKY43	MK511281	1149	382	1	2(WRKYGQK)	C-X4-C-X22-HXH	C2H2
GgWRKY44	MK511282	1296	431	2b	WRKYGQK	C-X5-C-X23-HNH	C2H2
GgWRKY45	MK511283	786	261	2e	WRKYGQK	C-X5-C-X23-HNH	C2H2
GgWRKY46	MK511284	1356	451	1	2(WRKYGQK)	C-X4-C-X22-HXH	C2H2
GgWRKY47	MK511285	1530	509	1	2(WRKYGQK)	C-X4-C-X22-HXH	C2H2
GgWRKY48	MK511286	594	197	2c	WRKYGQK	C-X4-C-X23-HNH	C2H2
GgWRKY49	MK511287	789	262	2c	WRKYGQK	C-X4-C-X23-HNH	C2H2
GgWRKY50	MK511288	468	155	2f	WRKYGQK	C-X4-C-X22-HXH	C2H2
GgWRKY51	MK511289	894	297	2c	WRKYGQK	C-X4-C-X23-HXH	C2H2
GgWRKY52	MK511290	732	243	2a	WRKYGQK	C-X5-C-X23-HNH	C2H2
GgWRKY53	MK511291	945	314	2a	WRKYGQK	C-X5-C-X23-HNH	C2H2
GgWRKY54	MK511292	867	288	2a	WRKYGQK	C-X5-C-X23-HNH	C2H2
GgWRKY55	MK511293	882	293	2a	WRKYGQK	C-X5-C-X23-HNH	C2H2
GgWRKY56	MK511294	750	248	2a	WRKYGQK	C-X5-C-X23-HNH	C2H2
GgWRKY57	MK511295	783	260	2f	WRKYGQK	C-X4-C-X22-HNH	C2H2
GgWRKY58	MK511296	936	311	2c	WRKYGQK	C-X4-C-X23-HXH	C2H2
GgWRKY59	MK511297	1014	337	2f	WRKYGQK	C-X4-C-X22-HXH	C2H2
GgWRKY60	MK511298	651	216	2c	WRKYGQK	C-X4-C-X23-HNH	C2H2
GgWRKY61	MK511299	732	243	2a	WRKYGQK	C-X5-C-X23-HNH	C2H2
GgWRKY62	MK511300	429	142	2a	WRKYGQK	C-X5-C-X13-HN	C2H2
GgWRKY63	MK511301	1092	363	2d	WRKYGQK	C-X5-C-X23-HNH	C2H2
GgWRKY64	MK511302	1050	349	2d	WRKYGQK	C-X5-C-X23-HNH	C2H2
GgWRKY65	MK511303	1065	354	2e	WRKYGQK	C-X5-C-X23-HNH	C2H2
GgWRKY66	MK511304	1047	348	2d	WRKYGQK	C-X5-C-X23-HNH	C2H2
GgWRKY67	MK511305	339	112	2c	WRKYGKK	C-X4-C-X23-HNH	C2H2
GgWRKY68	MK511306	588	195	2c	WRKYGKK	C-X4-C-X23-HNH	C2H2
GgWRKY69	MK511307	933	310	2d	WRKYGQK	Zinc cluster	
GgWRKY70	MK511308	981	326	2d	WRKYGQK	Zinc cluster	
GgWRKY71	MK511309	1071	356	2d	WRKYGQK	C-X5-C-X23-HNH	C2H2
GgWRKY72	MK511310	957	318	2d	WRKYGQK	C-X5-C-X23-HNH	C2H2
GgWRKY73	MK511311	387	128	2d	WRKYGQK	C-X5-C-X23-HNH	C2H2
GgWRKY74	MK511312	1089	362	2f	WRKYGQK	C-X4-C-X22-HNH	C2H2
GgWRKY75	MK511313	690	229	2c	WRKYGQK	C-X4-C-X23-HNH	C2H2
GgWRKY76	MK511314	1005	334	2c	WRKYGQK	C-X4-C-X23-HNH	C2H2
GgWRKY77	MK511315	1068	355	3	WRKYGQK	C-X7-C-X23-HXC	C2HC
GgWRKY78	MK511316	723	240	2f	WRKYGQK	C-X4-C-X22-HNH	C2H2
GgWRKY79	MK511317	690	229	2c	WRKYGQK	C-X4-C-X23-HXH	C2H2
GgWRKY80	MK511318	651	216	2c	WRKYGQK	C-X4-C-X23-HNH	C2H2
GgWRKY81	MN625734	912	303	2d	WRKYGQK	Zinc cluster	
GgWRKY82	MN625735	780	259	2d	WRKYGQK	Zinc cluster	
GgWRKY83	MK511319	591	196	NG	WRKYGQK		
GgWRKY84	MK511320	531	176	NG	WRKYGQK		
GgWRKY85	MN625736	654	217	NG	WRKYGQK		
GgWRKY86	MN625737	552	183	NG	WRKYGQK		
GgWRKY87	MN625738	426	141	NG	WRKYGKK

**Table 3:** Physical parameters of GgWRKY genes.

GgWRKYs	pI	Mw(Da)	Instability Index	Aliphatic index	GRAVY	Subcellular localization
GgWRKY1	6.64	56446.57	61.13	58.15	−0.760	Nucleus
GgWRKY2	6.61	42812.25	57.19	57.60	−1.054	Nucleus
GgWRKY3	4.87	30524.69	67.49	59.14	−1.006	Nucleus
GgWRKY4	6.55	31717.61	41.31	63.19	−0.743	Nucleus
GgWRKY5	6.51	28190.94	53.76	48.50	−1.096	Nucleus
GgWRKY6	6.17	22124.23	61.94	44.85	−1.109	Nucleus
GgWRKY7	7.18	64879.58	59.87	41.12	−0.988	Nucleus
GgWRKY8	8.89	41166.98	53.56	46.53	−0.984	Nucleus
GgWRKY9	8.63	52606.42	52.87	52.55	−0.903	Nucleus
GgWRKY10	5.30	19325.09	30.20	43.99	−1.340	Nucleus
GgWRKY11	5.91	39455.37	50.96	49.92	−0.843	Nucleus
GgWRKY12	5.74	82181.16	54.43	52.75	−0.836	Nucleus
GgWRKY13	5.91	39455.37	50.96	49.92	−0.843	Nucleus
GgWRKY14	5.63	45531.95	51.96	55.39	−0.773	Nucleus
GgWRKY15	6.12	48273.38	66.44	38.82	−1.086	Nucleus
GgWRKY16	7.70	44055.51	38.82	55.39	−0.859	Nucleus
GgWRKY17	5.52	24792.96	45.33	50.05	−1.027	Nucleus
GgWRKY18	5.81	58714.96	51.32	61.23	−0.758	Nucleus
GgWRKY19	6.34	56060.64	52.16	59.04	−1.002	Nucleus
GgWRKY20	6.45	70757.15	58.09	61.11	−0.700	Nucleus
GgWRKY21	6.86	63675.28	53.72	64.60	−0.622	Nucleus
GgWRKY22	8.10	38457.09	52.91	70.20	−0.784	Nucleus
GgWRKY23	5.81	26383.71	47.29	48.25	−1.033	Chloroplast
GgWRKY24	5.51	28633.96	61.55	58.22	−0.728	Nucleus
GgWRKY25	5.73	23136.95	55.89	59.32	−0.679	Nucleus
GgWRKY26	5.72	23236.34	54.16	65.89	−0.515	Nucleus
GgWRKY27	6.01	59133.88	60.52	54.94	−0.827	Nucleus
GgWRKY28	6.86	22645.0	61.66	65.27	−1.052	Nucleus
GgWRKY29	8.22	41362.95	60.27	59.34	−0.720	Nucleus
GgWRKY30	5.32	40108.97	49.44	63.18	−0.690	Nucleus
GgWRKY31	6.88	52267.98	57.49	55.46	−0.992	Nucleus
GgWRKY32	5.96	18879.89	55.37	53.15	−1.062	Chloroplast
GgWRKY33	6.30	18917.71	60.53	48.97	−1.024	Nucleus
GgWRKY34	5.44	36176.01	68.68	69.84	−0.771	Nucleus
GgWRKY35	6.23	38723.22	62.50	48.22	−1.141	Nucleus
GgWRKY36	9.60	22200.15	58.51	60.94	−0.886	Nucleus
GgWRKY37	6.25	26645.01	52.90	74.20	−0.690	Nucleus
GgWRKY38	8.72	40884.31	52.12	61.53	−0.570	Nucleus
GgWRKY39	5.56	67756.87	46.23	59.11	−0.720	Nucleus
GgWRKY40	6.32	29579.37	51.55	75.57	−0.747	Nucleus
GgWRKY41	7.63	45591.88	53.87	62.46	−0.896	Nucleus
GgWRKY42	6.48	37329.15	62.93	56.14	−1.051	Nucleus
GgWRKY43	9.09	41765.72	55.64	55.60	−0.958	Nucleus
GgWRKY44	7.99	47206.16	55.82	75.66	−0.515	Nucleus
GgWRKY45	5.42	28438.55	60.89	58.35	−0.670	Nucleus
GgWRKY46	7.22	49789.82	50.10	66.08	−0.735	Nucleus
GgWRKY47	7.28	55366.60	54.68	60.33	−0.935	Nucleus
GgWRKY48	8.96	21525.73	39.86	56.85	−0.812	Nucleus
GgWRKY49	6.75	28771.61	45.23	57.98	−0.842	Nucleus
GgWRKY50	7.77	17309.33	33.92	59.74	−0.684	Nucleus
GgWRKY51	7.09	32735.09	63.33	46.09	−0.864	Nucleus
GgWRKY52	9.23	27088.46	43.69	63.74	−0.833	Nucleus
GgWRKY53	8.70	34991.34	51.44	61.72	−0.825	Nucleus
GgWRKY54	8.98	31973.96	50.45	59.86	−0.840	Nucleus
GgWRKY55	7.00	32897.26	52.03	76.45	−0.568	Nucleus
GgWRKY56	8.88	27837.52	52.35	71.20	−0.610	Nucleus
GgWRKY57	5.69	29064.88	64.72	53.27	−0.994	Nucleus
GgWRKY58	6.06	34087.41	45.82	54.15	−0.973	Nucleus
GgWRKY59	7.16	35981.76	53.20	57.63	−0.706	Nucleus
GgWRKY60	9.13	23611.08	39.40	57.27	−0.838	Nucleus
GgWRKY61	9.23	27088.46	43.69	63.74	−0.833	Nucleus
GgWRKY62	9.65	16437.91	45.33	74.15	−0.896	Nucleus
GgWRKY63	9.70	40591.79	54.87	66.61	−0.769	Nucleus
GgWRKY64	9.68	38091.79	59.72	63.52	−0.724	Nucleus
GgWRKY65	5.58	38415.26	54.79	53.79	−0.706	Nucleus
GgWRKY66	9.73	39237.38	51.34	61.93	−0.809	Nucleus
GgWRKY67	9.61	13291.91	58.98	41.70	−1.143	Nucleus
GgWRKY68	6.23	22180.23	60.65	45.95	−1.086	Nucleus
GgWRKY69	9.86	34964.59	52.37	67.94	−0.742	Nucleus
GgWRKY70	9.89	36444.10	55.54	68.19	−0.728	Nucleus
GgWRKY71	9.24	38874.59	47.53	66.29	−0.631	Nucleus
GgWRKY72	9.91	34646.20	51.39	65.06	−0.586	Nucleus
GgWRKY73	9.96	14291.46	33.70	58.67	−0.802	Mitochondria
GgWRKY74	8.36	39382.65	55.22	53.40	0.858	Nucleus
GgWRKY75	7.60	25909.53	42.02	51.05	−0.895	Chloroplast
GgWRKY76	6.27	37066.81	56.89	53.95	−0.845	Nucleus
GgWRKY77	5.45	40568.91	49.05	57.13	−0.830	Nucleus
GgWRKY78	4.93	26513.78	63.03	54.08	−1.010	Nucleus
GgWRKY79	8.31	26029.34	43.79	62.14	−0.738	Nucleus
GgWRKY80	9.13	23611.08	39.40	57.27	−0.838	Nucleus
GgWRKY81	9.96	33185.38	63.25	62.54	−0.746	Nucleus
GgWRKY82	10.05	28317.13	59.62	64.40	−0.639	Nucleus
GgWRKY83	9.11	22167.36	37.16	66.68	−0.463	Nucleus
GgWRKY84	7.73	19597.61	32	57.05	−0.807	Chloroplast
GgWRKY85	5.58	23670.73	64.14	37.83	−1.053	Nucleus
GgWRKY86	9.05	20003.87	35.94	76.72	−0.551	Cytoplasm
GgWRKY87	6.28	16102.53	55.86	43.55	−1.207	Nucleus

### Conserved domain in Glycyrrhiza WRKY members

**Figure 1:**
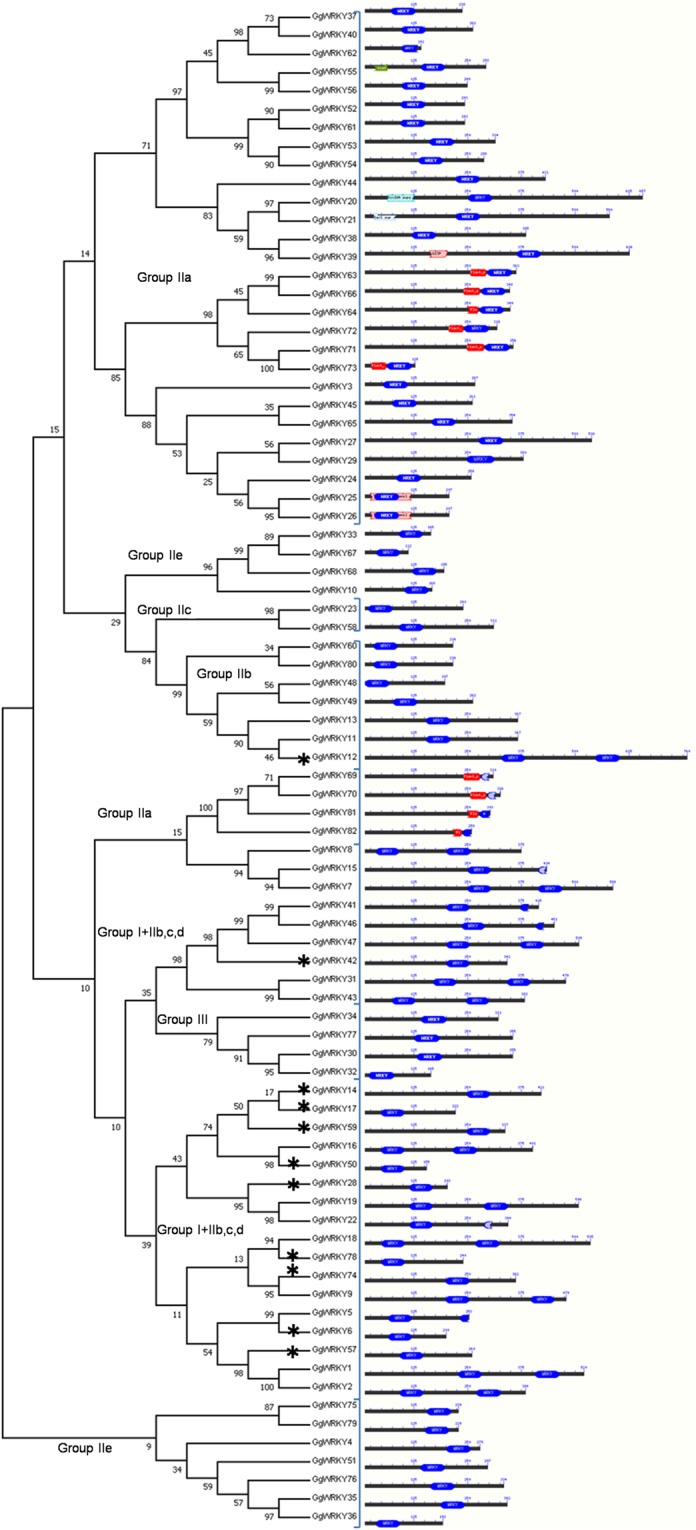
Classification of full-length GgWRKY amino acid sequences with different conserved domains (DivIVA, SerS, bZIP, Coat & Plant Zn cluster, WRKY). The conserved domains were investigated by CDD; * are exceptions in the classified groups and sub-groups in the phylogeny.

**Figure 2:**
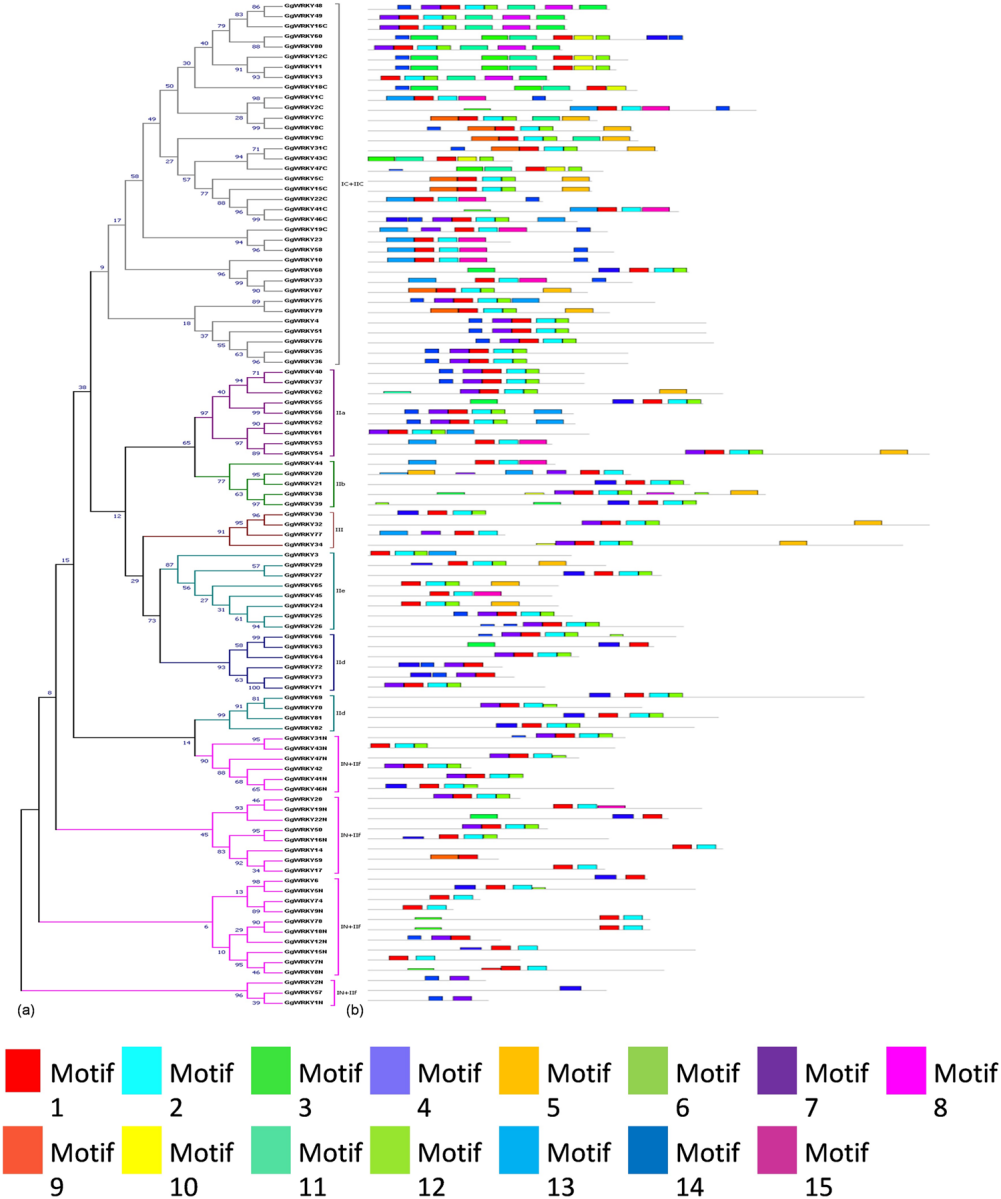
(**a**) Visualization of classification of 82 GgWRKY proteins. Conserved regions of GgWRKYs were used to construct the NJ phylogenetic tree with 1000 bootstrap value**. (b)** Architecture of 15 conserved protein motifs in GgWRKYs. Each motif is represented in different color (Motif 1–15). The conserved motifs were predicted by MEME program.

Generally, similar domains in a protein impart similar function. Transcription factors gene families have a common conserved domain involved in DNA binding. All the 147 WRKYs (*G. glabra* & *G. uralensis*) had a distinctive hepta-peptide DNA binding sequence (WRKYG[Q/K]K), the identifying character of the WRKY family. In the present study, 28 WRKYs showed the presence of additional motif besides the WRKY domain (Figs. 1 and S1). GgWRKY55 possessed Coat family motif (30 amino acid residues) at the N-terminal while GgWRKY20 and GgWRKY21 had DivIVA super-family (63 amino acid residues) and SerS Superfamily motif (61 amino acid residues), respectively at the C-terminal. *G. uralensis*, on the other hand, had GuWRKY9 having Exo70 exo cyst complex subunit (338 amino acid) and Flac-arch super (GuWRKY26) at the N-terminal and PAT1 (GuWRKY23) and SGNH_hydrolase (GuWRKY60) at the C-terminal. However two motifs were common in both the species-Plant Zinc Cluster (26–40 amino acid) in 16 WRKYs and bZIP domain (42amino acid) in 2 WRKYs was present in both the species. Plant Zn cluster super-family domain was present in nine GgWRKYs (GgWRKYs63,64, 66, 69,70,71,72,73 and 86) and seven GuWRKYs (GuWRKYs1,13,18,29,35,49 and 56). All the domains simultaneously reported in the present study in both the species of Glycyrrhiza were reported individually in different plants in earlier studies. We have not come across any report mentioning DivIVA, SerS Superfamily, PAT1, Exo70 exo cyst complex subunit SGNH_hydrolase, Flac-arch super & Coat family protein in plant WRKY proteins. However, bZIP & plant zinc cluster have been reported from *A. thaliana* earlier^36^. The analysis of sequence motifs using MEME platform (http://meme.nbcr.net/meme/cgi-bin/meme.cgi)^37^ displaying common and unique motifs within the GgWRKY sequences are shown in Fig. 2.

### Phylogeny

**Figure 3:**
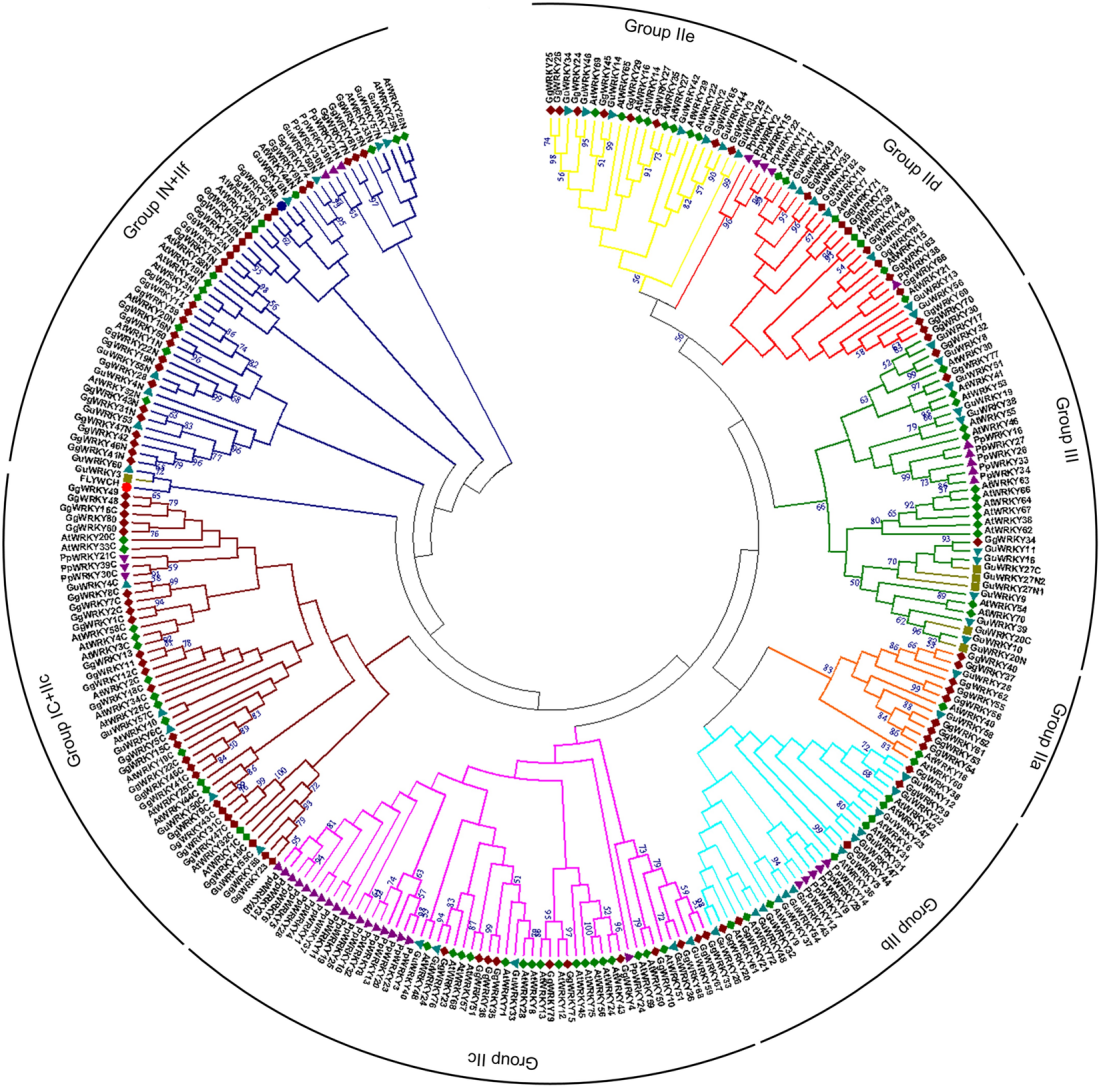
Neighbour-Joining JTT model of phylogenetic tree comprising of 82 *Glycyrrhiza glabra* (maroon), 54 *Glycyrrhiza uralensis* (cyan blue), 70 *Arabidopsis thaliana* (dark green), 37 *Psycometrella patens* (violet), with GCMa (blue) and FLYWCH CRAa (red) WRKY domains. Suffix ‘N’ and ‘C’ indicates the N-terminal and the C-terminal of 60 amino acids WRKY domains of Group I.

The relatedness among 136 Glycyrrhiza WRKY proteins with the 109WRKYs identified from *Arabidopsis thaliana*, *Psychometrella patens*, Human FLYWCH CRAa and GCMa were investigated (Fig. 3) and tabulated in Table 4. The phylogeny of 136 WRKY proteins from the genus Glycyrrhiza displayed 22WRKYs (17GgWRKYs & 5GuWRKYs) belonging to group-I, 98 WRKYs (61 GgWRKYS & 37 GuWRKYs) clustering in group-II and 16 WRKY members comprising of group-III (4GgWRKYS & 12GuWRKYs). Group-II was further sub-divided into five sub-groups, IIa (11), IIb (17), IIc (16 + 8), IId (17), IIe (15) and an additional novel sub-group IIf (14) based on WRKY transcription factor rules adopted in Arabidopsis^9^. The present paper reports few exceptions observed in the WRKY members identified in the genus Glycyrrhiza. The GuWRKY27 possessed three WRKY domains (N1, N2 &C). Few recent publications have also reported more than 2 WRKY domains in *Gossypium raimondii*^38^, *Linum usitatissimum Lupinus angustifolius, Aquilegia coerulea* and *Setaria italic*^32^. Phylogenetic analysis of the indicated proteins, however clubbed them into different subgroups. For example, in G. *raimondii* (WRKY108) the three domains (WRKY108N1, WRKY108N2 &WRKY108C) were clustered into IIc, III & IId sub-groups, respectively. In the present study, however, all the three WRKY domains (N1, N2 &C) of GuWRKY27 were found to be clustered into Group-III having Zn finger pattern similar to groupIII. This implies that the GuWRKY27 protein sequences are highly homologous to the group III WRKY member proteins, unlike the earlier published reports. Another exception was seen in GuWRKY20, where the protein was classified into group I based on the number of WRKY domains (2). However, it was clustered into group-III in the phylogenetic classification. MSA revealed that both the WRKY domains had Zn finger pattern similar to Group-III (C-X_7_-C-X_23_-HXC). The third exception was observed in GuWRKY3 whose Zn finger pattern was unlike any of the existing subgroups of group II. It could be the starting point for the evolution of a new subgroup in group II.

**Table 4:** Phylogenetic classification of WRKY domains identified from *G. glabra*, *A. thaliana*, *P. patens* and *G. uralensis* WRKY proteins.

Group	Sub group	Gene number				*GgWRKY*s	*AtWRKY*s	*PpWRKY*s	*GuWRKYs*
		*GgWRKY*s	*AtWRKY*s	*PpWRKY*s	*GuWRKYs*				
I	IN	17	13	3	5	41,46,47, 31,43,1,2,15,9,16, 19,22,18, 12,7,8,5	58,20,1, 32,3,4,19,44,2,34, 33,25,26	30,39,21	4,57,6,50,55
	IC	17	13	3	5	19,41,46, 22,15,5, 47,31,43, 12,18,9,1,2,7,8,16	58,4,3,33,20,2,26, 34,19,25, 44,32,1	39,30,21	4,57,6,50,55
II	IIa	9	3	0	2	53,54,61, 52,55,56, 62,37,40	40,60,18	--------	58,28
	IIb	5	8	5	12	20,21,44, 38,39	36,6,31, 42,47,61, 9,72	7,9,12, 14,29	54,43,5,12, 22,23,47,31,37,48,32,26
	IIc	11	17	19	5	36,35,76, 51,79,75, 4,10,68, 67,33	45,75,43, 24,56,48, 57,23,68, 71,8,28, 13,12,50, 51,59	37,11,25, 10,3,23, 20,8,13, 32,1,4,19,31,40,28, 5,6,24	40,24,33,36,59
	IId	10	7	5	7	70,69,81, 82,66,63, 64,72,71, 73	11,17,15, 21,39,74, 7	15,22,17, 2,38	1,49,35,13, 56,29,18
	IIe	8	8	0	7	27,29,3, 65,25,26, 24,45	65,69,29, 27,22,16, 14,35	--------	46,34,14,2, 44,25,42
	IC + IIc	8	1	0	0	58,23,60, 80,48,49, 11,13	10	---------	---------
	IN + IIf	10	0	0	4	78,28,50, 14,59,17, 74,6,57,42	---------	------------	53,7,60,3
III		4	13	5	12	34,30,32, 77	63,64,66, 67,38,62, 54,70,55, 46,30,41, 53	16,27,26, 33,34	9,16,11,39, 10,19,38,8, 17,51,20N, 20C,27N1, 27N2,27C
Total		82	70	37	54				

It was further observed among the 82 GgWRKY proteins in the phylogenetic tree, Group IN (17 members) clubbed with ten GgWRKYs (GgWRKYs 59,-14,-17,-28,-50,-42,-6,-57,-74,-78) belonging to Group-II with unique Zn finger pattern (C-X_4_-C-X_22_-HXH) which was not reported earlier in this group. We propose a new subgroup-IIf based on the present findings which could be the initiation of divergence into a new sub-group maintaining the characteristic WRKY domain.

The phylogenetic analysis of the 60 amino acid region of the Glycyrrhiza WRKY proteins indicated their diverse origin. The N-terminal and the C-terminal of Group I of the WRKY proteins clustered them into different clades indicating their dissimilar background. Further, the majority of the subgroup IIc proteins (8 proteins) were found to assemble with group IC indicating their common origin with respective clusters. Contrary to our results, Zhu *et al*.^39^ found that subgroup IIc WRKY domain in *Triticum aestivum*, originated from the N-terminal WRKY domain of group I. However, recent study on legumes have revealed that IIc sub-groups have multiple origins^32^. The present study also showed gathering of sub-groups IIa & IIb, while sub-group IId + IIe were clustered with group III, signifying close relationship with members with respective groups. Previously Zhang & Wang^8^ proposed a phylogenetic tree based evolutionary relationships which classified the *WRKY* gene family into four clades including groups I + IIc, groups IIa + IIb, group IId, and group IIe. But according to Rinerson *et al*.^40^ hypothesis the WRKY protein evolution may have followed two alternative paths, “Group I Hypothesis” which proposed that all WRKY proteins evolved from the C-terminal WRKY domains of group I proteins, and the “ IIa + b Separate Hypothesis” which suggested that groups IIa and IIb have evolved directly from a single domain algal gene separated from a group I-derived lineage. It is hard to explain the origin of the *WRKY* gene family on the basis of any one hypothesis, as mounting number of studies have demonstrated their multiple origins. Based on our phylogenetic analyses, we found that a phylogenetic cluster was a mix of *WRKY* genes from at least two different groups or sub-group indicating their dynamic nature.

Further, the present study could identify eleven WRKY proteins (GgWRKY-83 to-87 & GuWRKY-15, -21, -30, -41, -45 &-52), not included in the phylogenetic analyses, that possessed WRKY domain but had truncated characteristic zinc finger motif. Earlier studies on *Vitis venifera*^41^ and rice^42^ had also shown loss of Zn finger motifs in WRKY proteins. The phylogenetic clustering was further examined at sequence level by multiple sequence alignment (MSA).

### Multiple sequence alignment of the identified WRKY proteins

**Figure 4:**
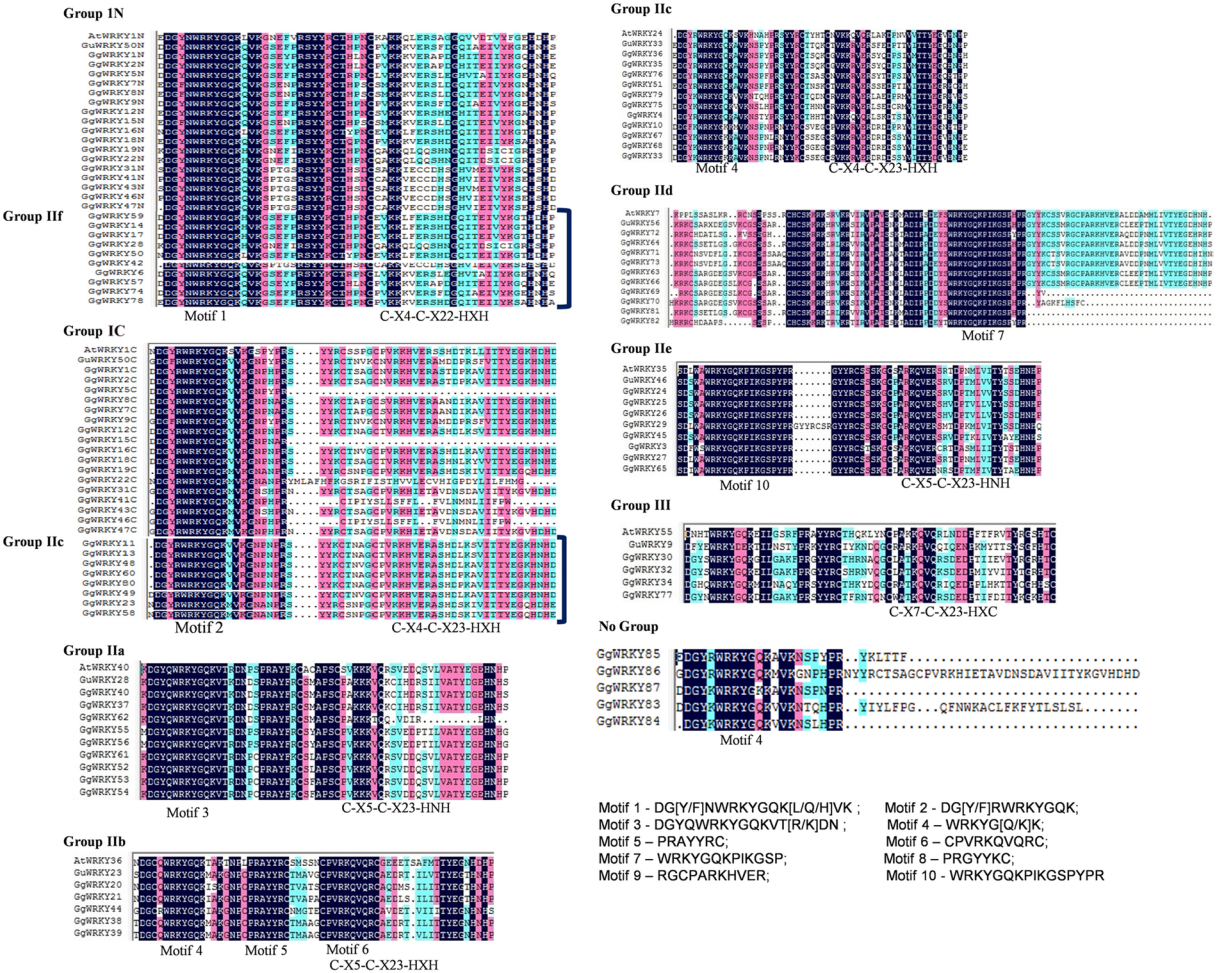
Multiple sequence alignment (MSA) of conserved GgWRKY domain. The alignment was performed using Clustal W program and displayed using DNAMAN software. Conserved motifs (1–10) and type of zinc-finger pattern are indicated within groups or sub-groups. Blue color represents 100% sequence identity. Pink color is for more than 75% while cyan color is for less than 75% sequence identity.

**Figure 5:**
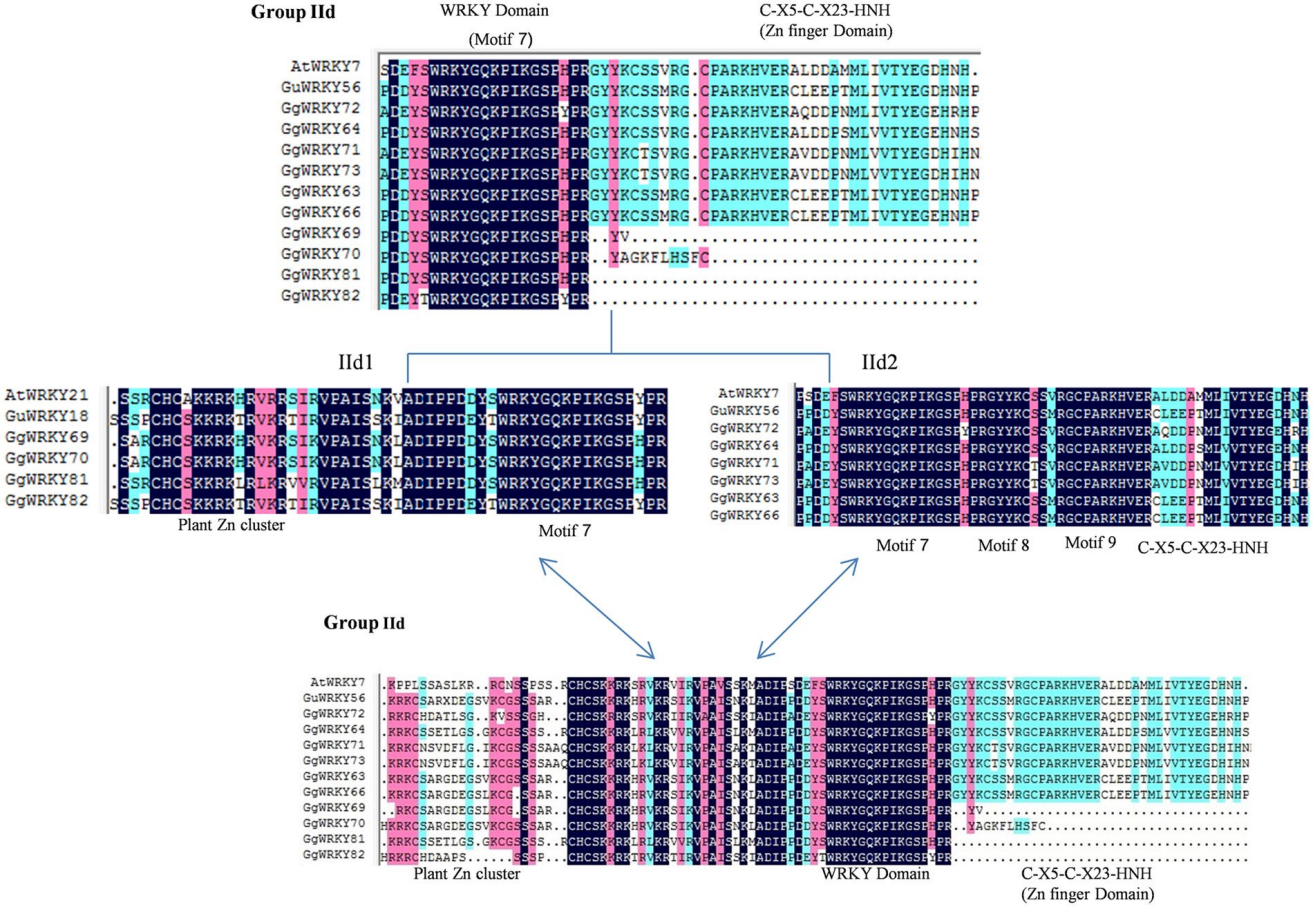
Multiple sequence alignment (MSA) profile of group IId (10 sequences). Initially conserved 60 amino acids region is used to build alignment that showed low sequence identity (32%). When it was separated in two groups (IId1&IId2), identity increased significantly (54.9& 83.7%). Sub-group IId1 (4 sequences) with sequences upstream to WRKY domain having Plant Zinc cluster with motif 7and no zinc finger; subgroup IId2 (6 sequences) with 7, 8, 9 motifs and Zn finger. When four sequences of sub group IId1 were extended 50 amino acids towards N’terminal (total110 amino acid), sequence identity of sub groupIId increased to 70.68%.

The multiple sequence alignment of 60 amino acids conserved region of all the 87 GgWRKY proteins were clustered in 9 different groups and sub-groups with very high homology (>70%) as shown in (Fig. 4). Group IN displayed conserved motif 1 (DG[Y/F]NWRKYGQK[L/Q/H]VK) and zinc finger pattern of C-X_4_-C-X_22_-HXH showing conservancy with 27 GgWRKY proteins, 17 of them belonged to Group IN and 10 GgWRKYs (59,-14,-17,-28,-50,-42,-6,-57,-74,-78) belonging to new sub-group IIf, having Zn finger motif (C-X_4_-C-X_22_-HXH) which was similar to Zn finger domain of group IN unlike group II members. While Group IC had 17 GgWRKYs belonging to group IC and 8 GgWRKYs from group IIc (GgWRKYs 11,-13,-48,-60,-80,-49,-23,-58). All the 25 GgWRKY members in Group IC displayed conserved motif 2 (DG[Y/F]RWRKYGQK), zinc finger pattern of C-X_4_-C-X_23_-HXH and high identity (70.4%) as shown in Fig. 4. The third group IIa had nine GgWRKY proteins (GgWRKYs40,-37,-62,-55,-56,-61,-52,-53,-54) displaying motif 3 (DGYQWRKYGQKVT[R/K] DN) and a zinc finger motif pattern of C-X_5_-C-X_23_-HNH having 86.2% identity, except GgWRKY62 which had C-X_5_-C-X_13_-HN Zn finger pattern. Group IIb had five GgWRKYs (GgWRKYs 20,-21,-44,-38,-39) with three conserved motifs, motif 4 (WRKYG[Q/K]K), motif 5 (PRAYYRC) and motif 6 (CPVRKQVQRC) with 85.8% identity, while Group IIc comprised of 11 sequences (GgWRKYs35,-36,-76,-51,-79,-75,-4,-10,-67,-68,-33) with conserved sequence motif 4 (WRKYG[Q/K]K) and a zinc finger motif pattern of C-X_4_-C-X_23_-HXH (77.6% identity). The 60 amino acid signature sequence of Group IId proteins comprising of 10 GgWRKYs, when aligned together showed only 32% identity (Fig. 5). Six of the ten members had zinc finger, while four (GgWRKYs 69,-70,-81,82) had no zinc finger present in them; instead they had 50 amino acid zinc cluster domain at the N terminal. Based on the conservancy, when this group was divided into two sub-groups IId1 (GgWRKYs 69,-70,-81,-82) & IId2 (GgWRKYs 72, -64,-71,-73,-63,-66,) the identity was significantly increased from 32% to 54.9% and 83.7%, respectively. The members clustered in group IId1 showed conserved motif 7(WRKYGQKPIKGSP) and no zinc finger at the C-terminal end, while subgroup IId2 displayed conservancy of three motifs- 7(WRKYGQKPIKGSP), 8(PRGYYKC) & 9(RGCPARKHVER) along with a common zinc finger pattern C-X_5_-C-X_23_-HNH (Fig. 5). Further, when conserved domain sequence of 60amino acid of all the 10 GgWRKYs of sub-group IId, was increased to 110 amino acids the two sub-groups (IId1&IId2) combined into a single group (IId) displaying 70.68% identity among all the members. We also confirmed the conservancy of each group and subgroup with the WRKY members belonging to *A. thaliana* and *G. uralensis* (Figs. 4 and 5). The MSA further proves the dynamic nature of GgWRKYs.

### Promoter analysis

**Figure 6:**
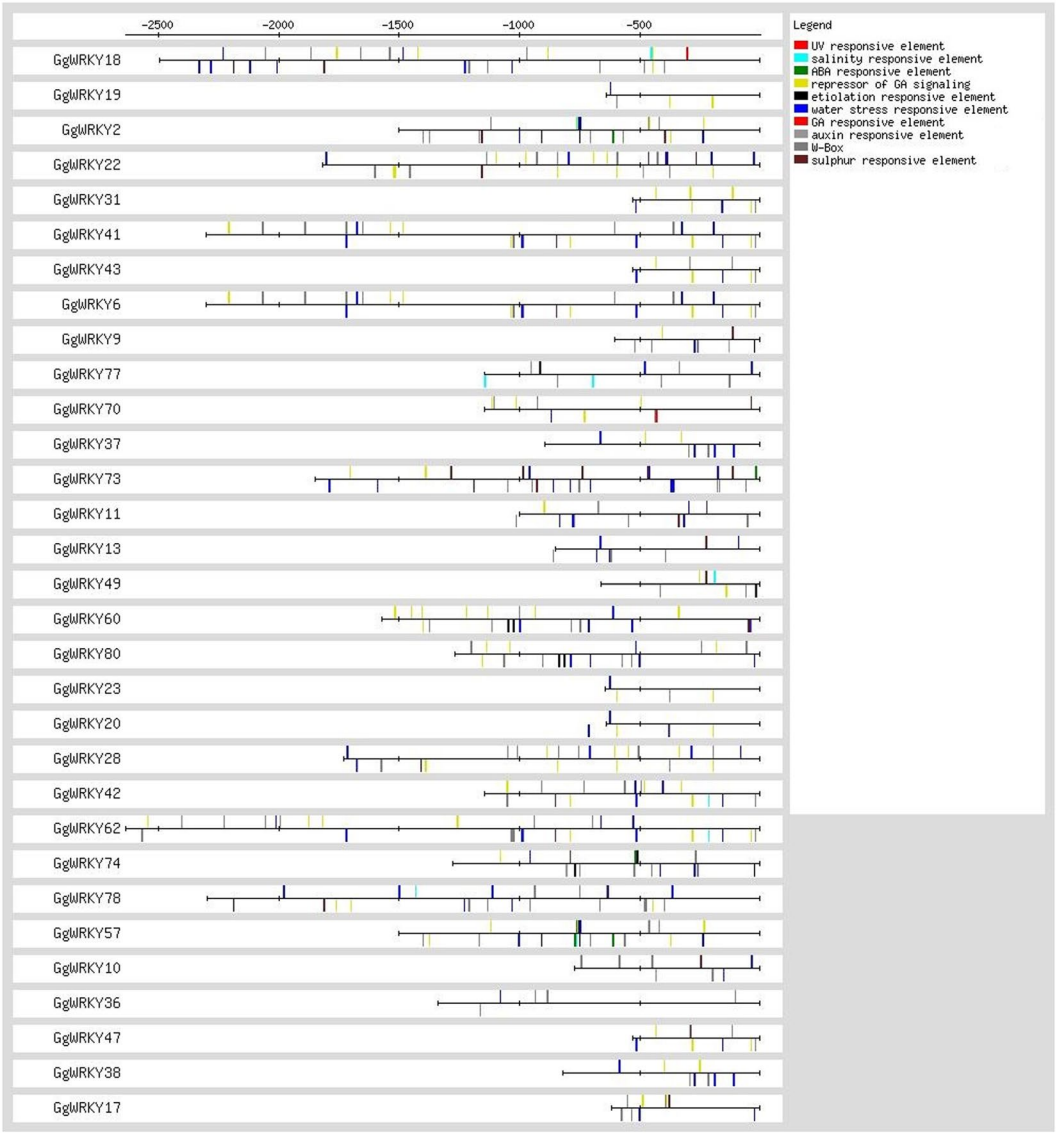
Analysis of cis-regulatory elements (CREs) in GgWRKY promoter region. Total ten stress responsive elements were mapped on sense and anti-sense strand using RSAT tool.

The upstream region of 31 *GgWRKY* genes was examined for the presence of *Cis*-regulatory elements. Several stress-responsive elements like UV, salinity, ABA, GA signalling, etiolation, water stress, auxin and sulphur responsive elements were identified (Fig. 6). Also, several copies of *WRKY* binding motifs were identified in the promoter region of *GgWRKY* genes. The DNA binding WRKY motifs in the promoter region ranged from 1 (*GgWRKY*20, 23) to 11 (*GgWRKY*s 18 &62). Overall, twenty-seven *GgWRKY*s had three or more W-boxes in their promoter region. Observation revealed presence of multiple W-box elements mostly in the stress-related genes, which is following the earlier studies^6,12^. Additionally promoters of several glycyrrhizin biosynthesis genes (CYP88D6, CYP72A154 & squalene epoxidase) contained W boxes (unpublished data) suggesting regulatory role of *WRKY* in glycyrrhizin biosynthesis, thereby providing a platform to understand its regulation.

### Protein-protein interaction

**Figure 7:**
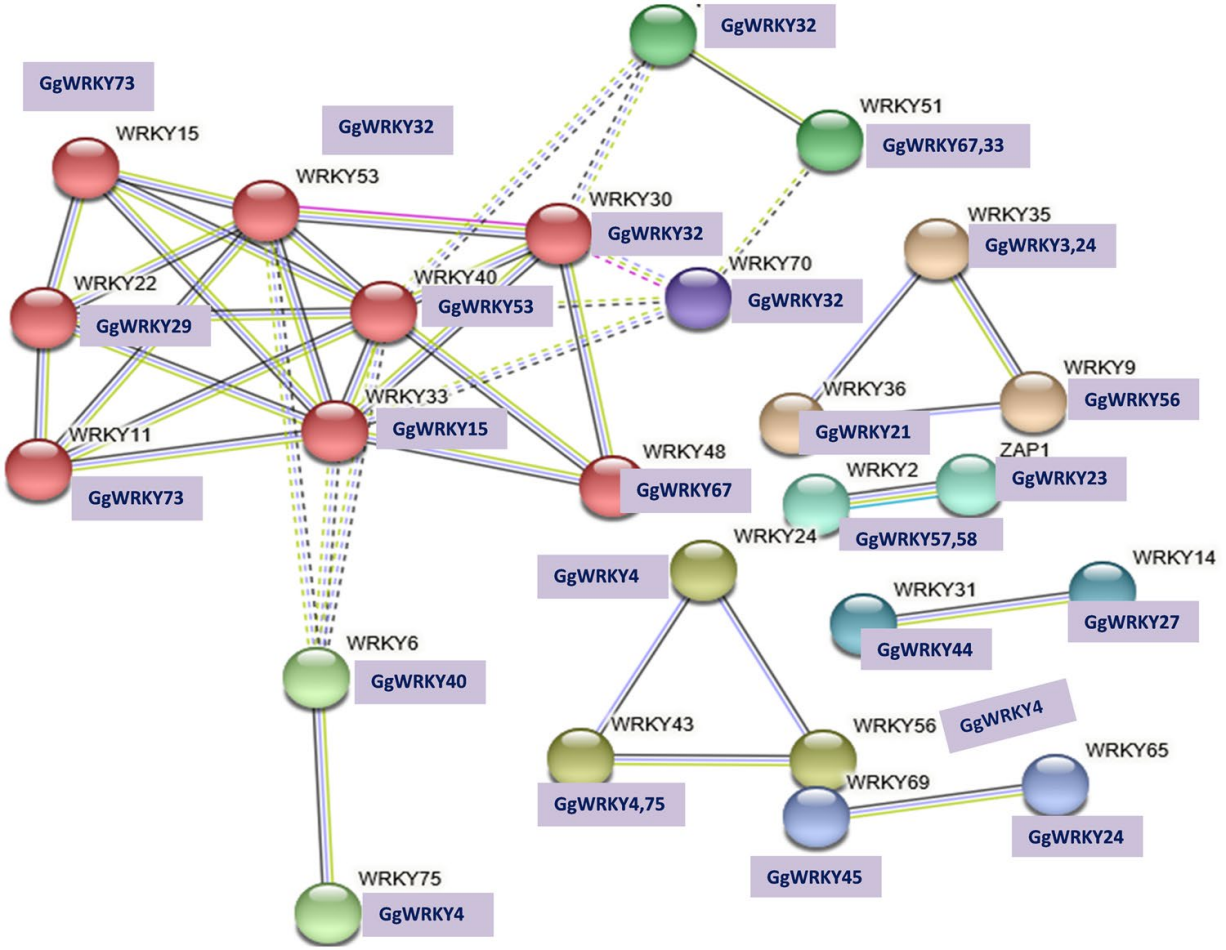
Protein-Protein interaction of GgWRKYs transcription factor based on AtWRKYs orthologs as predicted by STRING search tool.

The protein-protein interaction of GgWRKYs was predicted by STRING^43^ with *A. thaliana* (taxonomic ID 3702) as a model using Markov clustering (MCL) having inflation factor of 8.5. The STRING software is a prediction pipeline for deducing protein-protein associations from co-expression data and interaction conservation (Fig. S2; Supplementary File 3). It predicts interaction between orthologs in taxonomically different organism. The corresponding GgWRKY orthologs selected had more than 60% protein sequence homology having one WRKY domain (PF03106) as predicted by Pfam and three domains (IPR003657, IPR003657 and IPR017412) as analysed by INTERPRO. The analysis revealed 74, 08 and 03 GO term significantly enriched in biological processes, molecular function and cellular components, respectively (Supplementary File 4). The MCL clustering displayed 8 distinct groups, largest being associated with 8 WRKY proteins (red) showing strong interaction (AtWRKYs 15,22,11,33,40,53,30 &48) corresponding to predicted orthologs GgWRKYs 73, 29, 73, 15, 53, 32, 32 & 67, respectively (Fig. 7; Supplementary File 5). These specific associations indicated that these proteins jointly contributed to a shared function of *cis* or trans in nature as inferred from curated databases or experimentally determined data available in public domains^43^. The AtWRKYs and corresponding GgWRKYs were shown to be involved in various biological processes including ROS induced modulation, plant growth and osmotic stress (AtWRKY15/GgWRKY73), development (AtWRKY22/GgWRKY29), Jasmonic acid-induced response (AtWRKY11/GgWRKY73), wound-induced response and positive regulator of stress (AtWRKY33/GgWRKY15), senescence (AtWRKY40/GgWRKY53), leaf development and senescence (AtWRKY53/GgWRKY32), abiotic stress and senescence (AtWRKY30/GgWRKY32), and hormonal signal response and defense (AtWRKY48/GgWRKY67). Strong association between 8 AtWRKYs and corresponding ortholog GgWRKYs indicated co-regulation of several biological processes related to senescence, Jasmonate response, hormonal signaling and wound induced response (Supplementary File 5).

The associated proteins need not physically connect in a protein-protein interaction of a specific step instead, they may form functional protein linkages especially in transcriptional or post-transcriptional regulation of a process. Also, it has been observed that evolutionarily related proteins usually maintain their three-dimensional structure, even when they have diverged^43,44^. This interaction between orthologs is expected to display high degree of interaction conservation more so in indirect or transient types of protein-protein associations. Based on protein conservancy of GgWRKYs with AtWRKYs, we assessed the putative functions of GgWRKYs and verified the expression profile of few of the predicted functions of GgWRKYs experimentally in Lab (Table 5) under abiotic stress.

**Table 5:** AtWRKYs, their induction factor and experimentally verified responses in GgWRKYs.

S.N	GgWRKYs	Orthologs AtWRKYs	Orthologs (% Identity)	Reported Induction factor (AtWRKYs)	Experimentally verified Induction factor (GgWRKYs)
1	*GgWRKY2*	*AtWRKY4*	*5*3*%*	*P. syringae, Salicyclic acid (SA), Jasmonic acid (JA), sucrose, senescence, cold, salinity*	*Senescence, Carbon starvation, NAA, GA*_*3*_
2	*GgWRKY4*	*AtWRKY24*	*74%*	*unknown*	*salinity, Carbon starvation*
3	*GgWRKY5*	*AtWRKY4*	*58%*	*P. syringae, SA, JA, sucrose, senescence, cold, salinity*	*Senescence, Salinity, dark, GA*_3_
4	*GgWRKY8*	*AtWRKY*3*3*	*61%*	*Salinity, mannitol, cold, heat*, *H2O2, ozone, UV, chitin, B. cinerea, P. syringae, A. brassiciola*	*Senescence, Salinity, Carbon starvation, NAA, GA*_*3*_
5	*GgWRKY14*	*AtWRKY20*	*5*3*%*	*unknown*	*Heat, cold, Salinity. UV, GA*_*3*_
6	*GgWRKY15*	*AtWRKY*3*3*	*62%*	*Salinity, mannitol, cold, heat*, *H2O2, ozone, UV, chitin, B. cinerea, P. syringae, A. brassiciola*	*Senescence, cold, Carbon starvation, NAA, GA*_*3*_
7	*GgWRKY20*	*AtWRKY61*	*51%*	*unknown*	*GA*_3_
8	*GgWRKY24*	*AtWRKY65*	*52%*	*Fe starvation*	*Dark, heat, UV, Salinity*
9	*GgWRKY29*	*AtWRKY22*	*64%*	*H2O2, dark, chitin, flagellin*	*Senescence, cold, Nwrky*
10	*GgWRKY36*	*AtWRKY28*	*77%*	*Salinity, mannitol, H2O2*	*Salinity, heat, UV, dark, Carbon starvation*
11	*GgWRKY*3*8*	*AtWRKY61*	*77%*	*unknown*	*Senescence, dark, NAA, GA*_*3*_
12	*GgWRKY40*	*AtWRKY6*	*60%*	*H2O2, methyl viologen*, *Pi and B starvation*	*Cold, dark*
13	*GgWRKY44*	*AtWRKY*3*1*	*67%*	*unknown*	*GA*_*3*_
14	*GgWRKY45*	*AtWRKY69*	*64%*	*unknown*	*Senescence, dark, Carbon starvation, GA*_3_
15	*GgWRKY51*	*AtWRKY71*	*75%*	*unknown*	*Dark, Carbon starvation, GA*_3_
16	*GgWRKY53*	*AtWRKY40*	*55%*	*ABA signaling, SA, chitin, wounding*	*Wounding, cold, dark*
17	*GgWRKY54*	*AtWRKY40*	*58%*	*ABA signaling, SA, chitin, wounding*	*Senescence, wounding, cold, dark, NAA, GA*_3_
18	*GgWRKY55*	*AtWRKY40*	*4*3*%*	*ABA signaling, SA, chitin, wounding*	*Senescence, UV, cold, NAA, GA*_*3*_
19	*GgWRKY56*	*AtWRKY9*	*51%*	*unknown*	*Cold, dark, NAA*
20	*GgWRKY57*	*AtWRKY2*	*78%*	*Salinity,manitol,ABA*	*Salinity, cold, dark, GA*_3_
21	*GgWRKY58*	*AtWRKY2*	*64%*	*Salinity, manitol, ABA*	*Cold, Carbon starvation, GA*_3_
22	*GgWRKY59*	*AtWRKY20*	*54%*	*unknown*	*Cold, GA*_3_
23	*GgWRKY62*	*AtWRKY18*	*69%*	*ABA, SA*	*Salinity, heat, cold*
24	*GgWRKY69*	*AtWRKY 21*	*65%*	*unknown*	*Cold, dark*
25	*GgWRKY70*	*AtWRKY 21*	*63%*	*unknown*	*Salinity, cold, dark*

### Real-time expression analysis

**Figure 8:**
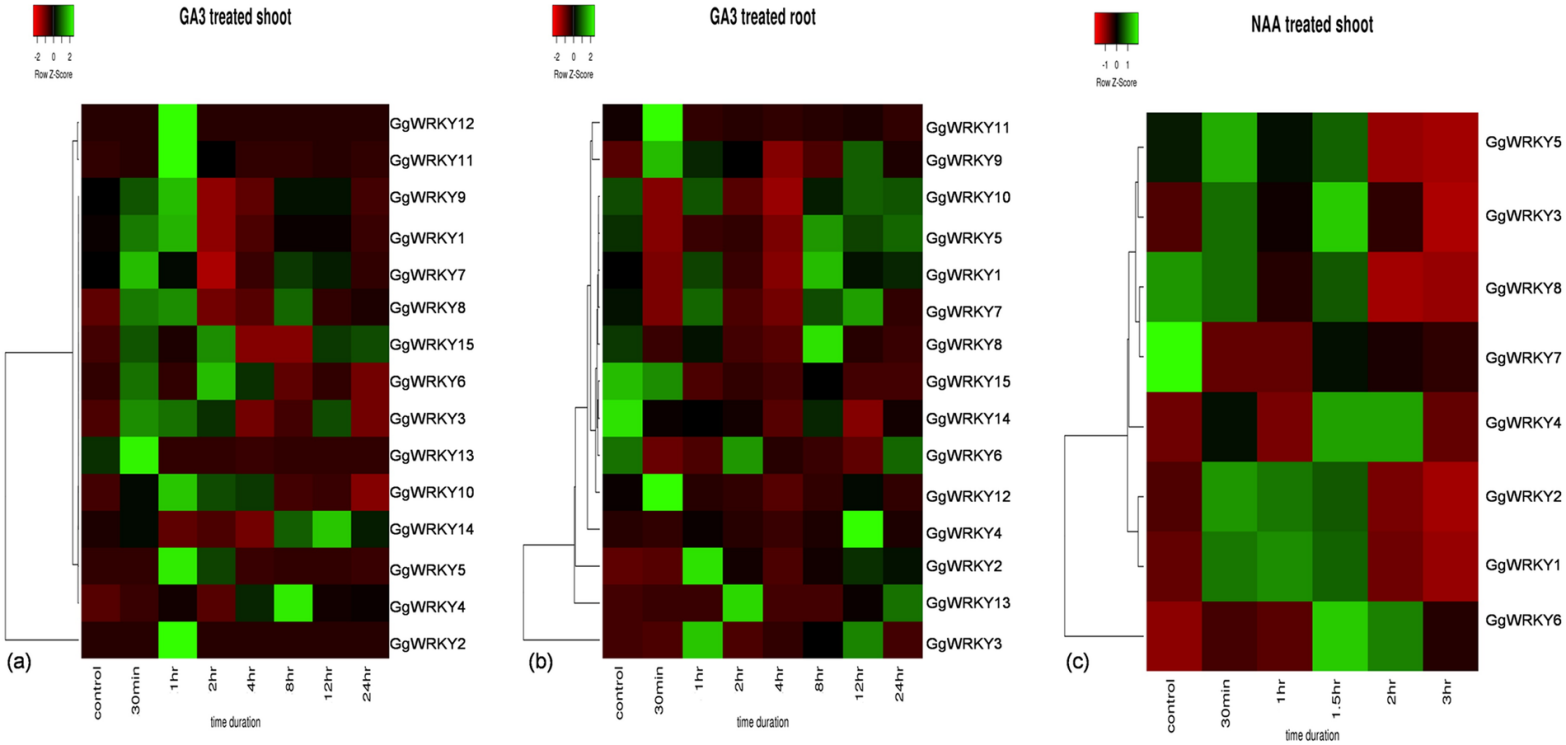
GgWRKY genes are represented as rows and treatment time duration as columns in the matrix. Expression analysis of selected *GgWRKY* genes displaying differential expression pattern in shoot and roots under various hormonal stress. Heat map showing- Cluster analysis of *GgWRKY* genes according to their expression profiles in (**a**) shoots and (**b**) roots after GA_3_ treatment for 0.5, 1, 2, 4, 8, 12 and 24 h time interval; **(c**) Cluster analysis of *GgWRKY* genes according to their expression profiles in shoots after NAA treatment for 0.5, 1, 1.5, 2 and 3 h time interval.

**Figure 9:**
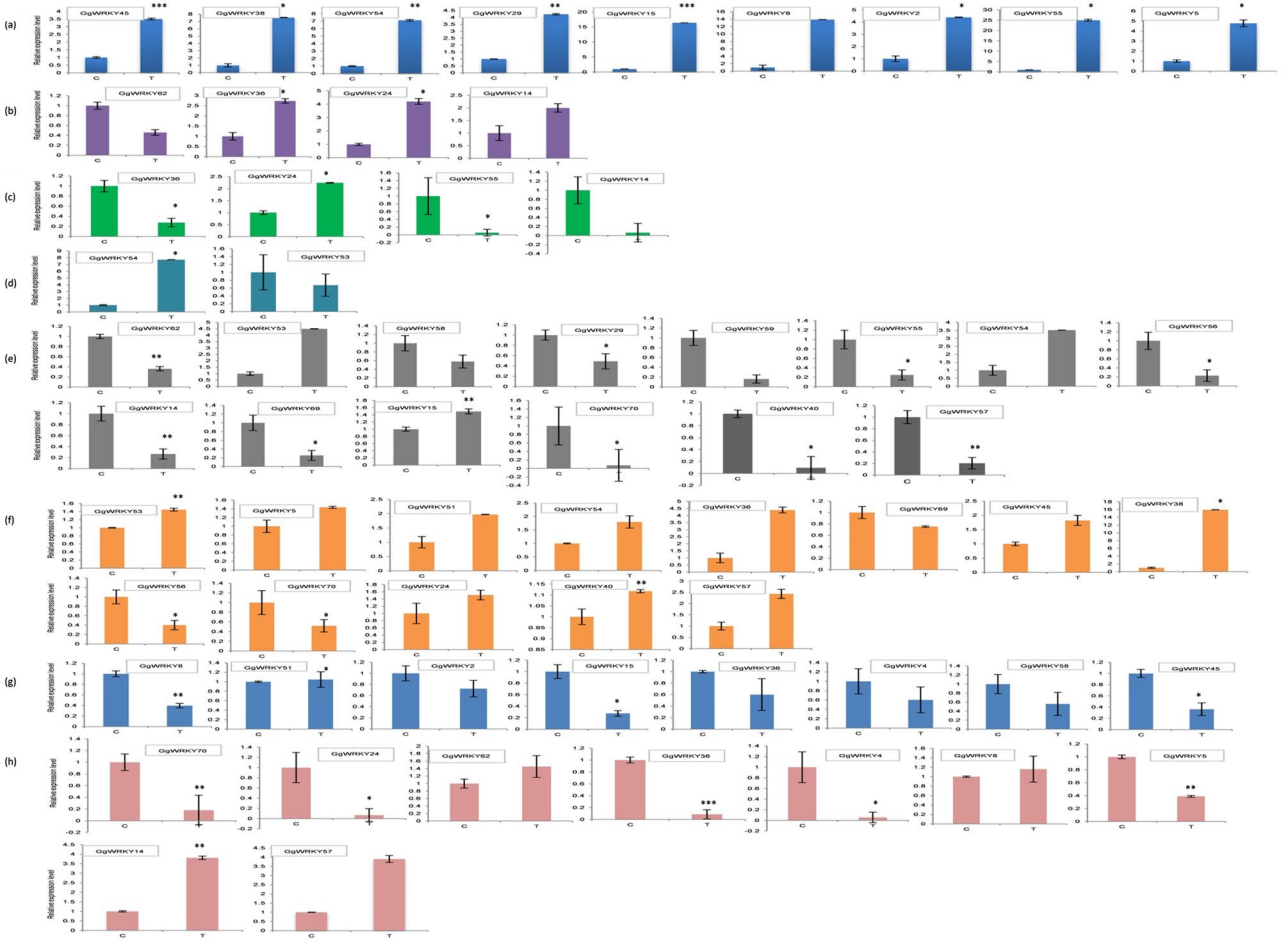
Expression profiles of selected GgWRKY genes under eight different stresses. The Y-axis indicates relative expression level and X-axis indicates control shoot tissues (C) and treated shoot tissues (T). (**a**) expression patterns under etiolated conditions; (**b–h**) expression profiles under heat, UV, wounding, cold, dark, carbon starvation and salinity, respectively. Actin was used as internal reference. Three biological replicates were used to calculate error bars using standard deviation. Asterisks indicate that the corresponding gene was significantly up- or down regulated in a given treatment (*P ≤ 0.05; **P ≤ 0.01; ***P ≤ 0.001).

The *A. thaliana* based protein conservancy for the functional prediction of putative orthologs in *G. glabra* was experimentally performed. The expression profile of twenty-five *GgWRKY* genes was investigated post-hormonal treatments (NAA & GA_3_) and under eight abiotic stress treatments including carbon starvation, salinity, heat, cold, dark, UV, senescence and wounding administered to the aerial tissues of the *in-vitro* cultured *G. glabra* plant. Out of the 25 *GgWRKY*s examined, eight *GgWRKY*s responded to the NAA treatment (Fig. 8). As can be seen from the heat map, transcripts of *GgWRKY*s 8, 15 & 29 accumulated maximum (4.1, 3.3 &1.6 folds, respectively) between 0.5 to 1.00 hrs, *GgWRKY*55 took longer (1.30 hrs) to display its maxima (17.5 folds). *GgWRKY*s 54 & 56 were mostly up-regulated all the time, while *GgWRKY*s 4 & 38 were down-regulated in the specified time of study. It seems *GgWRKY*56 & *GgWRKY* 38 had definite positive (3.3 folds) & negative regulatory (0.001folds) effects, respectively on the aerial tissues of the plant treated with auxin. GA_3_ treatment, on the other hand (Fig. 8), revealed *GgWRKY* 58 was highly up-regulated (257.3 folds) in the aerial tissues of the plant, while *GgWRKY*15 was up-regulated (43.6 fold) in the underground tissues of the plant as compared to the control. Most of the *GgWRKY*s responded within 1.5 hrs of GA_3_ treatment, except *GgWRKY* 20 which took longer to show their maxima (2.0 hrs) in the root tissues. The results inferred from the present study were compared with the earlier published reports on the functions of At*WRKY*s^45^ which are presented in Table 5. Of the 25 *GgWRKY*s assessed for abiotic stress treatment, maximum showed response to post-cold treatment (17), followed by dark (13). Nine *GgWRKY*s responded to senescence and salinity, while eight triggered a response on carbon starvation. Maximum number of *GgWRKY*s were up-regulated (10) after dark treatment followed by senescence (9). Darkness induced up-regulation of *GgWRKY*s 5, 24, 36, 38, 40, 45, 51, 53, 54 and 57, while *GgWRKY*s 56, 69 & 70 were down-regulated. Nine *GgWRKY*s 2, 5, 8, 15, 29, 38, 45, 54 & 55 were up-regulated during senescence, *GgWRKY*s 5, 14, 24 & 54 were down-regulated under saline conditions. The transcript levels of *GgWRKY*s 14, 24 & 36 were more under heat stress, while *GgWRKY* 24 was up-regulated on UV treatment. The injured plant showed up-regulated transcripts of *GgWRKY* 54, while *GgWRKY*53 was found to be down-regulated. Cold treated samples showed higher transcript levels of *GgWRKY*s 15, 53 & 54, while *GgWRKY*s14, 40, 55, 56, 57, 58, 59, 62, 69 &70 were down-regulated. The Carbon starved plants showed up-regulation of only *GgWRKY*51 while *GgWRKY*s 2, 4, 8, 15, 36, 45 & 58 were found to be down-regulated (Fig. 9). Significantly up-regulated (P ≤ 0.001) Gg*WRKY*s were observed only in senescence (*GgWRKYs* 45&15), while in salinity, *GgWRKY*36 was significantly down-regulated. Out of the ten different treatments performed to assess the role of GgWRKYs in abiotic stress, predicted by STRING based on AtWRKY protein conservancy, the response of 11 GgWRKYs corroborated very well with 15 AtWRKYs whose functions were reported in literature (Table 5). Among the 25 GgWRKYs examined, 23 responded to abiotic stress, 17 were induced by hormone while 13 were common to both, suggesting role of hormone under stress conditions. Further study on these functionally assigned GgWRKYs will throw light on their role in underlying molecular mechanism. On comparing the experimental data with the STRING predicted data, it was found that our results corroborated well with the earlier reports on the induction of AtWRKY4 on senescence, AtWRKY40 on wounding, AtWRKYs 2, 28 &33 during salinity and AtWRKY33 under cold treatments. Few AtWRKYs whose functions were not assigned, like AtWRKYs 21, 24, 31, 38, 61, 69 and 71, were also designated putative function based on identity percentage.

In any biological process, understanding the role of transcription factor provides an insight into its regulatory mechanism. WRKY transcriptional factors have been extensively studied in a plant for plant growth, development, and response to biotic and abiotic stresses. However, *WRKY* genes present in Glycyrrhiza species have not been elucidated. In conclusion, we identified and characterized 147 full length putative *WRKY* genes in the genus *Glycyrrhiza*. These putative genes were grouped based on the number of WRKY domains & zinc finger pattern and further analysed for various properties like molecular weight, iso-electric point, instability index, sub-cellular location. The phylogenetic analysis categorised more than one group/sub-group together, indicating their multiple origins. The present paper highlights several findings not reported earlier, like the novel Zn finger motif of C-X_4_-CX_22_-HXH type (sub-group IIf). Also these group-II members shared homology with group IN WRKY members, unlike the other members of group II. This paper also reports several additional domains (DivIVA, SerS, Coat, Exo70 exo cyst complex subunit, Flac-arch super, PAT1and SGNH_hydrolase) apart from the conserved WRKY domain in the WRKY proteins. MSA based 60 amino acid signature sequence of group IId showed very low sequence identity (32%), however when its length was increased to 110 amino acid the identity increased to 70.7%. A closer look at the subgroup IId showed presence of 50 amino acid plant Zn cluster domain upstream to the WRKY domain in four members. However, characteristic Zn finger motif was absent in these members.

Additionally, putative functions were assigned to the identified GgWRKYs, based on STRING database which comprised of both theoretically reported and experimentally verified data. Verification of the data in the Lab displayed 11 out of 15 functions as assigned. The study provides significant evidence to further investigate and validate the role of WRKYs in Glycyrrhiza species in growth, under stress condition and in secondary metabolite biosynthesis.

## Materials and Methods

### Identification and sequence annotation of *WRKY* genes

Transcriptome-wide identification of *WRKY* genes in *G. glabra* and *G. uralensis* transcriptome data was done by local similarity (tblastn) search and HMM profile methods. Initially, seventy AtWRKY proteins were downloaded from Arabidopsis Information Resource (TAIR; http://www.Arabidopsis.org/), and HMM profile of WRKY family with accession number PF03106 was retrieved from the Pfam protein family database (https://pfam.xfam.org/). The *A. thaliana* (AtWRKYs) and PF03106 profile were used as a query sequence to search against the transcriptome data of *G. glabra* and *G. uralensis*. An e-value cut off of 1e^−50^ was applied for the homologue recognition. Parsing the BLAST data from *G. glabra*, a total of 125 contig hits were found. All these contigs were further analysed in ORF finder (https://www.ncbi.nlm.nih.gov/orffinder/) to get the full-length CDS of 87 *GgWRKY* sequences. Publicly available transcriptome database of *G. uralensis* (http://ngs-data-archive.psc.riken.jp/Gur-genome/download.pl.) was used to get 60 *GuWRKYs*. The retrieved coding sequences (CDSs) were then translated by ExPASy translate (https://web.expasy.org/translate/) tool and validated using the Uniprot protein database (https://www.uniprot.org/), conserved domain database (https://www.ncbi.nlm.nih.gov/cdd/) and HMMScan (https://www.ebi.ac.uk/Tools/hmmer/search/hmmscan). The molecular weight (MW), Theoretical isoelectric point (pI), instability index, aliphatic index, Grand average of hydropathicity (GRAVY) of GgWRKY proteins were predicted via the ProtParam (http://web.expasy.org/protparam/). Additionally, subcellular localisation was also predicted by an advanced protein subcellular localisation prediction tool WoLFPSORT (https://wolfpsort.hgc.jp/).

### Multiple sequence alignment, phylogenetic analysis and classification

The multiple sequence alignment (MSA) of 245 WRKY proteins was performed using 82 WRKY proteins of *G. glabra (*GgWRKY), 54 WRKY proteins from *G. uralensis* (GuWRKY), 70 from *A. thaliana (*AtWRKYs), 37 from *P. patens (*PpWRKYs) and one each from Human FLYWCH CRAa and GCMa. The protein sequences of *Arabidopsis* were downloaded from TAIR (http://www.Arabidopsis.org/), GuWRKYs from (http://ngs-data-archive.psc.riken.jp/Gur-genome/download.pl.), PpWRKYs were obtained from *P. patens* v3.3 (https://phytozome.jgi.doe.gov/pz/portal.html#!info?alias = Org_Ppatens), Human FLYWCH CRAa (EAW85440) and GCMa (BAA13651) were retrieved from NCBI (http://www.ncbi.nlm.nih.gov/protein/). The conserved regions of 60 amino acids for the WRKY proteins were searched using HMMScan and aligned using CLUSTALW for the construction of the phylogenetic tree. For the GgWRKY based phylogenetic tree, complete protein sequences were used. The tree was constructed using MEGA 7.0 with neighbor-joining method using JTT substitution model and pair-wise deletion method with 1000 bootstrap value. The 60 amino acid conserved region of MSA of *G. glabra* was visualised using DNAMAN. The MSA included conserved region of WRKY members representing each group and subgroup from *A. thaliana* and *G. uralensis* as reference.

### Protein-protein interaction analysis and motif detection

The conserved motifs of GgWRKY proteins were analyzed using Multiple Expectation Maximization for Motif Elicitation (MEME: http://meme-suite.org/tools/meme) with the following parameters: minimum and maximum motif widths 6 and 50, respectively and the maximum number of motifs 15. Protein-protein interactions were predicted by STRING^43^ with *A. thaliana* as model using Markov clustering with inflation factor of 8.5.

### Analysis of cis-regulatory elements in Gg*WRKY*s promoter regions

Promoter sequences of 31 Gg*WRKY*s of up to 2.5 kb (kilobase) upstream to the transcription start site were retrieved manually (Supplementary File 6). These promoter sequences were used as queries to scan the presence of various *Cis*-regulatory elements in Plant *Cis*-acting Regulatory DNA Elements (PLACE, http://www.dna.affrc.go.jp/PLACE/)^46^. The position of identified CREs (biotic and abiotic stress-responsive elements) was mapped on both sense and anti-sense strand using RSAT^47^ (http://rsat.sb-roscoff.fr/feature-map_form.cgi) drawing tool.

### Plant material and treatments

Five months old *in-vitro* cultured plants grown in SPB medium^48^ under controlled conditions of 25 °C (±1.5) temperature and a 16 h light/8 h dark cycle (light intensity of 200mmol m–2 s–1), were exposed to various treatments including hormone, temperature, salinity, senescence and wounding. The plantlets, grown in liquid SPB medium were individually subjected to 50 µM auxin (NAA) for 0.5, 1.0, 1.5, 2.0 & 3.0 hrs, and 10 µM of gibberellin (GA_3_) treatments for 0.5, 1.0, 2.0, 4.0, 8.0, 12 & 24 hrs. Controls were sprayed with water. Different sets of plants were independently subjected to different abiotic treatments like NaCl (500 mM) for 72 hrs, dark, cold (4 °C) and heat (55 °C) treatments for 48, 24 and 8 hrs, respectively. For the Ultra-violet treatment, plants were kept under UV-C for 30 minutes. Mechanically injured plants were examined after 8 hrs of injury. Yellow aerial tissues of plants were used for senescence study, and Carbon starvation was given to the plant for 48 hrs in SPB medium having no sugar. All the respective controls were kept under culture conditions. The control and treated plants were harvested at the appropriate times as indicated, frozen in liquid nitrogen and stored at −80 °C for RNA extraction. Each treatment was used in triplicate and was repeated at least twice.

### RNA extraction and quantitative real-time reverse transcription PCR (qRT-PCR)

Total RNA of control and treated shoots and root tissues were extracted using the Pure Link RNA Mini Kit (Invitrogen, US). RNA integrity was analysed on a 1.5% agarose gel and quantity was determined using a NanoDrop 2000C spectrophotometer (Thermo Scientific, USA). cDNA synthesis was carried out using SuperScript™ VILO™ cDNA Synthesis Kit (Thermo Scientific, USA). qRT-PCRs were performed using the SYBR Green PCR Master Mix (Takara, Japan) and carried out in triplicate for each tissue sample. Gene-specific RT primers were designed manually (Supplementary File 7). The amplicons size ranged between 200 to 250 bp. A*ctin gene* was selected as an internal reference gene. The amplification was done in a ten μl reaction volume, which contained 5.0 μl of SYBR Green PCR Master Mix, 0.2 μl of each primer (10 pc), 0.2 μl of ROX, 1.0 μl cDNA template (150 ng/μl), and 3.4 μl ddH_2_O. PCRs with no-template controls were also performed for each primer pair. The real-time PCRs were performed employing7500 Fast Real-Time PCR System and software (Applied Biosystems, USA). All the PCRs were performed under following conditions: 30 sec at 95 °C, 3 sec at 95 °C, respective optimized Tm for 1 min (40 cycles) followed by 95 °C (15 seconds), 60 °C (30 sec) and 95 °C (15 sec) in MicroAmp fast reaction tubes (Applied Biosystems, USA). The specificity of amplicons was verified by melting curve analysis (55 to 95 °C) after 40 cycles.

## Supplementary information

Supplementary information.
