# Supplementary material for: A Comprehensive Transcriptome-Wide Identification and Screening of WRKY Gene Family Engaged in Abiotic Stress in Glycyrrhiza glabra
Source: Sci Rep. 2020 Jan 15;10:373. doi: 10.1038/s41598-019-57232-x (PMC6962277; doi:10.1038/s41598-019-57232-x)
Supplement: Supplementary file 1 — Supplementary information. [file 41598_2019_57232_MOESM1_ESM.docx]

**A Comprehensive Transcriptome-Wide Identification and Screening of *WRKY* Gene Family Engaged in Abiotic Stress *in* *Glycyrrhiza glabra***

**Pooja Goyal^a^, Malik Muzafar Manzoor^a^, Ram A Vishwakarma^a^, Deepak Sharma^b^, Manoj K Dhar^b^ & Suphla Gupta^a*^**

^a^Plant Biotechnology Department, Council for Scientific Research-Indian Institute of Integrative Medicine, Canal Road, Jammu

^b^Genome Research Laboratory, School of Biotechnology, University of Jammu, Jammu, India

***Corresponding authors Email ID: suphlabg@gmail.com**

**CSIR-IIIM-communication No.CSIR-IIIM/IPR/00103**

**Table S1 Protein sequence information of WRKYs in *G.glabra***

| Name | Gene ID | Protein Sequence |
| --- | --- | --- |
| GgWRKY1 | MK511239 | MSTTSAGVDAVMAAAPARPTITLPPRPTAEAFFAGGGGVSPGPMTLVSSFFANDSSATSFSQLLAGAMASPLSFAAAGDNNSGREDDGTHKGVGFKQSRPMNLVIARSPVFTIPPGLSPSGFLNSPGFFSPQSPFGMSHQQALAQVTAQAVLAQSHMHMQADYHQSSVTAPTEPVVEQPSFTLNEASEQQVVASAVSEPRNAQLETSELAHADKKYQPSSVAIDKPADDGYNWRKYGQKQVKGSEYPRSYYKCTHLNCPVKKKVERAPDGHITEIIYKGQHNHEKPQANRRVKDNTELNGNANIQSKSDSNSQSWIGNQNKVSESMPDCSMPESDQTSNQGAPRPLPGSSESEEVGDVDNREEGDDGEPNPKRRNTDVVVTEVPPSQKTVTEPKIIVQTRSEVDLLDDGYRWRKYGQKVVKGNPHPRSYYKCTSAGCNVRKHVERASTDPKAVITTYEGKHNHDVPAARNSSHNTASSNSMPLKPQAAVPEKHPLLKDMDFGNNDQRPVHLRLKEEQIIV |
| GgWRKY2 | MK511240 | MSHQQALAQVTAQAVLAQSHMHMQADYHQSSVTAPTEPVVEQPSFTLNEASEQQVVASAVSEPRNAQLETSELAHADKKYQPSSVAIDKPADDGYNWRKYGQKQVKGSEYPRSYYKCTHLNCPVKKKVERAPDGHITEIIYKGQHNHEKPQANRRVKDNTELNGNANIQSKSDSNSQSWIGNQNKVSESMPDCSMPESDQTSNQGAPRPLPGSSESEEVGDVDNREEGDDGEPNPKRRNTDVVVTEVPPSQKTVTEPKIIVQTRSEVDLLDDGYRWRKYGQKVVKGNPHPRSYYKCTSAGCNVRKHVERASTDPKAVITTYEGKHNHDVPAARNSSHNTASSNSMPLKPQAAVPEKHPLLKDMDFGNNDQRPVHLRLKEEQIIV |
| GgWRKY3 | MK511241 | MDSNASPQLESEEASSELKSETQASKKRKMVQKTVVAVRIGENVSKMKNEGLPSDFWSWRKYGQKPIKGSPYPRGYYRCSTSKGCSAKKQVERCRTDASMLIITYTSTHNHPGPTSLSTTNLSQQPKESETEETTEDPTVTSKEEDQQEQIEEEEMNKKHNHNVSSDEGINEENFHYLQSPIRCSENIIIIDQEDPFKLNTEKSHDRIDNLVLEEEPLCYAQLKNLSDSKSEELDFFDELEELPMPSSFLQFTRSIFSDERIPVAPS |
| GgWRKY4 | MK511242 | MYTCTYGCQTSSTPPSFFPFINFNPIEHSHFLSSISNMGDPMESQDPPPPPILMMTPQNNNNPFLFTPPTSMLQNPLDPHQGLLVADDNIDWGNLFCGQNNSNNNLLLVGDNIDDGNKGIAMDQCASSSSSLMMVNNEINGTSTDTHIHHRDQYQEEEEEKGNNNKKKEVERRVVKSGRVKKATRVPRFAFQTRSVDDILDDGYRWRKYGQKAVKNSTYPRSYYRCTHHTCNVKKQVQRLSKDTSIVVTTYEGIHNHPCEKLMETLTPLLKQIQFLASL |
| GgWRKY5 | MK511243 | MSVSSVLPATSLTQVPAATSTYTTQPQMPPSMEGSQAAMTESTNYSLPEQRLQQSSSLNVDKPADDGYNWRKYGQKQVKGSEFPRSYYKCTRPNCLVKKKVERSLEGHVTAIIYKGEHNHQRPHPNKRSKDAVTSNDHSNMQGIADSSYQGITTNSMSKMDPESSQATAEHLSGTSDSEEVGDHETEVEEKSGEPDPKRRNTEVTHSDPASSHRTVTEPRIVVQTTSEVDLLDDGYRWRKYGQKVVKGNPYPR |
| GgWRKY6 | MK511244 | MSVSSVLPATSLTQVPAATSTYTTQPQMPPSMEGSQAAMTESTNYSLPEQRLQQSSSLNVDKPADDGYNWRKYGQKQVKGSEFPRSYYKCTRPNCLVKKKVERSLEGHVTAIIYKGEHNHQRPHPNKRSKDAVTSNDHSNMQGIADSSYQGITTNSMSKMDPESSQATAEHLSGTSDSEEVGDHETEVEEKSGEPDPKRR |
| GgWRKY7 | MK511245 | MASPSPTINSNPRSFSSLPPQFMKTSSFTNNNNNSSNMMTMDDDHHQNNWTTFEHNHQQVGLDVPKFKSVQPPSLPLSPSPISPSSYFTSFSSGFSPTEFFNSPLFLSSPNAFASPTSEAFAGQSFNWKNSSGEDQQQGDKEDEKNYSDFSFQTQTKPTPVFQSSSSMSQVQEPIKKQDIWKSNEPTKQTDYSSERVAAKSDQYPTSQSISSEMAPSKPETHSNSVPRSGYLNYTASASLSVREQRRSEDGFNWRKYGQKQVKGSENPRSYYKCTHPSCSMKKKVERSLDGQITEIVYKGNHNHPKPQSTRRTSSQPSSSCTNSGISDQSVVTLGNPQMEPVSIQEDSSASVGEEDFEQTSQTSYSGGDEDALGPEAKRWKGDNENDGYSGLGSRTVREPRVVVQTTSEIDILDDGYRWRKYGQKVVKGNPNARSYYKCTAPGCSVRKHVERAANDIKAVITTYEGKHNHDVPSARGSATASYNMNRNSLNNSSVPAPIRPSAVNCYSSSSSFTNSLNNKTTRLPTNGNQGSFPLDMLQGPGSFGYSALGRSMDSFTNHGQCSDAAYSKAKDETKDDSFLQSFLSKDF |
| GgWRKY8 | MK511246 | MAPSKPETHSNSVPRSGYLNYTASASLSVREQRRSEDGFNWRKYGQKQVKGSENPRSYYKCTHPSCSMKKKVERSLDGQITEIVYKGNHNHPKPQSTRRTSSQPSSSCTNSGISDQSVVTLGNPQMEPVSIQEDSSASVGEEDFEQTSQTSYSGGDEDALGPEAKRWKGDNENDGYSGLGSRTVREPRVVVQTTSEIDILDDGYRWRKYGQKVVKGNPNARSYYKCTAPGCSVRKHVERAANDIKAVITTYEGKHNHDVPSARGSATASYNMNRNSLNNSSVPAPIRPSAVNCYSSSSSFTNSLNNKTTRLPTNGNQGSFPLDMLQGPGSFGYSALGRSMDSFTNHGQCSDAAYSKAKDETKDDSFLQSFLSKDF |
| GgWRKY9 | MK511247 | MMDIEEAERVVVAKPVASRPTCSTFKSFSELLAGAINASPPIAPSHQTTVSAIRPKTVRFKPAAAMNRPPAGSVSSQADIFGAALSNSSDMSPKPDTKQSLIYKPMAKLVSKTTVSLLANMGNGSTSQQQPRQSMEANLQHPNHEKFGTNMSSSLHQSIPRHAETYMATESCKVVQQNLEEDQKALTSSNTTNGDRPYDDGYNWRKYGQKQVKGSEFPRSYYKCTHPNCPVKKKVERSFDGQIAEIVYKGEHNHSKPQLPKRNTGSGTQQGSDGMVQDIWSNNNQSETNEGRIENNQNDTTGLSVRSAYHVKAPQPNDSALIGGAINAGGGSLENSCGLSGECEEGSKGFEAEEDEPRCKRRKNENQSNEAAASEEGLVEPRVVMQSSMDSEILGDGFRWRKYGQKVVKGNPYPRSYYRCTNVKCNVRKHVERAMDDPRSFVTTYEGKHNHEMPLIKNTGSSVASEKDSQASLSKDKPC |
| GgWRKY10 | MK511248 | MTDKNPKPPDSPDNEFTNQWPMDLSEYLKFDDDQWPEDDIETLVSGHVPNQDNQANEVGDFGGSGSGSHLEGSSSSRDVNSEREKKEARDRVAFKTKTEVEILDDGFKWRKYGKKMVKNSPNPRNYYRCSVEGCPVKKRVERDRDDPRYVITTYEGNHTHPVLARTQN |
| GgWRKY11 | MK511249 | MGPINPHVDMQVDNPEHVEQQNGVDGGDMGWANAQKGNITAGAVNRKPDNFEATSSASVGPEYCNQSTNLQAQNGTYFDSGDAVDGSSTFSNEEEEDDQGTHGSVSLGYDGEGDESESKRRKLESYAPELSGATRSIREPRVVVQTTSEVDILDDGYRWRKYGQKVVKGNPNPRSYYKCTNAGCTVRKHVERASHDLKSVITTYEGKHNHDVPAARASSHVNANASNGVPGQASGLVQNHVHRPEPSSQVHNGMGRPSLGSFNLPGRQQLGPSHGFSFGMNQPPMLSNLAMSGLGPAGHGPGPGPSQSKQLPVMPMHPFLAAQQQQRPSNEMGFMLPKGEPNVENMPPSGSSVYQEMMSRMPLGPHM |
| GgWRKY12 | MK511250 | MAGIDDNIALTGDWGFPSPSPRTLFSRMLEEDNNVTRPISEHPGNDSRTEDLFLGAHEPCETGRNGNTKDRAQDGDSGTQFSDVGFRTEQRSSSRGGLVERMAARAGFNAPRLNTESIRATDLSLNSDVQSPYLTIPPGLSPTTLLDSPVFLMNSLAQPSPTTGKFPFVSNGNIRCSELSSDAPEKCKDNNFDDIYASSFAFKPTNDSGSSFYHGAGRKINPTTLPQQSLPGIEASVQSENSFQSQSAEAIKVQTENKSGLHLQADFTELPPQKDNGIKTYLADQRAFDTVGGSIEHSTPLEVQADEEGDQRVNGDSLAAGAGGTPSEDGYNWRKYGQKQVKGSEFPRSYYKCTHPNCSVKKKVERSHEGHITEIIYKGAHNHPKPPPSRRSGMGPINPHVDMQVDNPEHVEQQNGVDGGDMGWANAQKGNITAGAVNRKPDNFEATSSASVGPEYCNQSTNLQAQNGTYFDSGDAVDGSSTFSNEEEEDDQGTHGSVSLGYDGEGDESESKRRKLESYAPELSGATRSIREPRVVVQTTSEVDILDDGYRWRKYGQKVVKGNPNPRSYYKCTNAGCTVRKHVERASHDLKSVITTYEGKHNHDVPAARASSHVNANASNGVPGQASGLVQNHVHRPEPSSQVHNGMGRPSLGSFNLPGRQQLGPSHGFSFGMNQPPMLSNLAMSGLGPAGHGPGPGPSQSKQLPVMPMHPFLAAQQQQRPSNEMGFMLPKGEPNVENMPPSGSSVYQEMMSRMPLGPHM |
| GgWRKY13 | MK511251 | MGPINPHVDMQVDNPEHVEQQNGVDGGDMGWANAQKGNITAGAVNRKPDNFEATSSASVGPEYCNQSTNLQAQNGTYFDSGDAVDGSSTFSNEEEEDDQGTHGSVSLGYDGEGDESESKRRKLESYAPELSGATRSIREPRVVVQTTSEVDILDDGYRWRKYGQKVVKGNPNPRSYYKCTNAGCTVRKHVERASHDLKSVITTYEGKHNHDVPAARASSHVNANASNGVPGQASGLVQNHVHRPEPSSQVHNGMGRPSLGSFNLPGRQQLGPSHGFSFGMNQPPMLSNLAMSGLGPAGHGPGPGPSQSKQLPVMPMHPFLAAQQQQRPSNEMGFMLPKGEPNVENMPPSGSSVYQEMMSRMPLGPHM |
| GgWRKY14 | MK511252 | MDAGEAHSGSELRRRGPGEAESEHEDPNRTGGYRPDSGADGAAASGTTVGARYKLMSPAKLPISRSPVLTIPPGLSPTAFLESPVLLSNMKVEPSPTTGSLPRLQQTAHGSVTSATSAAFPVTTVCFNTNTLDDGKSSFFEFKPHSRSNMVPAELDNRACEKSTQIDGQGKAQSFASSPLVKSEIAGPSNEISLSSPVQMVSSGASAHVEVDLDKLNSGGNIATGLQVSQVEGRGSGLSVAAERSSDDGYNWRKYGQKHVKGSEFPRSYYKCTHPNCEVKKLFERSHDGQITEIVYKGTHDHPKPQPNRRYSGGNIMSMQEERSDKASLTSRDDRGYNNYGQPSHTAEPDGTPELSPVATNDDSLEDAGFLSNRNNDEVDEDDPFSKRRFDNLGQSYSFCCLYLLADYKFSVEQKNGSWKC |
| GgWRKY15 | MK511253 | MASPSPTINSNPRSFSSLPPQFMKTSSFTNNNNNSSNMMTMDDDHHQNNWTTFEHNHQQVGLDVPKFKSVQPPSLPLSPSPISPSSYFTSFSSGFSPTEFFNSPLFLSSPNAFASPTSEAFAGQSFNWKNSSGEDQQQGDKEDEKNYSDFSFQTQTKPTPVFQSSSSMSQVQEPIKKQDIWKSNEPTKQTDYSSERVAAKSDQYPTSQSISSEMAPSKPETHSNSVPRSGYLNYTASASLSVREQRRSEDGFNWRKYGQKQVKGSENPRSYYKCTHPSCSMKKKVERSLDGQITEIVYKGNHNHPKPQSTRRTSSQPSSSCTNSGISDQSVVTLGNPQMEPVSIQEDSSASVGEEDFEQTSQTSYSGGDEDALGPEAKRWKGDNENDGYSGLGSRTVREPRVVVQTTSEIDILDDGYRWRKYGQKVVKGNPNAR |
| GgWRKY16 | MK511254 | MVSSGASAPVDADLDEFNHKGNTATGPQTSHVEVRGSGLSVAAEKVSNDGYNWRKYGQKLVKGSEFPRSYYKCTYPNCEVKKLFERSHDGQITEIIYKGTHDHPKPQPSNRYSVGAVMSMQGERSDKAFLAGRDDKASNMYGQDSTPELSPGATNDDNIEGAGFVSNRTNDEIDDDDPFSKRRRMELGNADFIPVVKPIREPRVVVQTLSEVDILDDGYRWRKYGQKVVRGNPNPRSYYKCTNVGCPVRKHVERASHDLKAVITTYEGKHNHDVPAARSSSHDMVGPSAASGSHTRIKLEESDTISLDLGMGLSSNRSNGQGKMLLSEYGDSQTHTSSSNSSFKFVNHTTNPSAPVYYGVLSNGSNPYGTSRENSRSDGRPSLNHSSYPCSQNMGRILMGP |
| GgWRKY17 | MK511255 | MVSSGASAHVEVDLDKLNSGGNIATGLQVSQVEGRGSGLSVAAERSSDDGYNWRKYGQKHVKGSEFPRSYYKCTHPNCEVKKLFERSHDGQITEIVYKGTHDHPKPQPNRRYSGGNIMSMQEERSDKASLTSRDDRGYNNYGQPSHTAEPDGTPELSPVATNDDSLEDAGFLSNRNNDEVDEDDPFSKRRFDNLGQSYSFCCLYLLADYKFSVEQKNGSWKC |
| GgWRKY18 | MK511256 | MPISRSEEASDESTLPENAIHGDDIGQQLVLEGEQKETSHATGVGRTSEDGYNWRKYGQKQVKGSEFPRSYYKCTQPNCPVKKKVERSHDGQITEIIYKSAHNHAKPHPNRRAPAPAPAPSTDEMSEIAEAGETYDKVDADSVWGNIQSAVKDTKHSLEWKADGQERTSSASVVTELSDPISMKRGRSLCMFESEDTPELSSTLASHDGDEDGATQAVVSVEDDAEDVESESKRRKKENYTVESILPPRNVREPRVVVQIESDVDILDDGYRWRKYGQKVVKGNPNPRSYYKCTSAGCTVRKHVERASHNLKYVVTTYEGKHNHEVPTARNSNQISSGDGGLPPSGANGQVALTLPGSASILKPETHQTLAPHFDRKPEFSNDFLRSSLLGGFSNDMKFGPSSISQMKYSSLNNTIPYGSYGLNPDRCTAPQAGSIASMFPDFPMPLPLNLPSSGSFSLAGINFNSVKPMASVQSFLSGQQVKEIDTGFLRLKQEQKDDTIYGTCIPSLEHSSSSLTPSSASPSIYQRVMQNFPS |
| GgWRKY19 | MK511257 | MVSSEESADHNVSSDKVQQRVSPQRDITLSQGNHDTEIHLSNPEGARSIPSIVAKNEVKDSDATACALQSDQEGRAHSLPREKHLQIPDSLSHELPPSQSGQDSPSIIREKVSKDGYNWRKYGQKHVKGNEFIRSYYKCTHPNCQAKKQLQQSHNGQITDSICIGRHSHPRPQSNTIVPDDRVLPVVEKEPHKPSLANVEDKSSVDHGCMPQQIKPLNSLPPSKVSGADKLKAAHVQLTAKYEVHNLEDPESKRLKKDSSNVDVTGVDMSTCESRVAVHTSSEVDFVNDGYRWRKYGQKMVKGNANPRSYYRCSNPGCPVKKHVERASHDSKIVITTYEGQHDHEIPSGRSVTHNAATNTHTMDIDGKLGTKSGGNTVCVDTGERNCLDSKSRLNKRPNEESITNSEAGDMAEFRVISLRNEGPENKLSQPEQQNEDSDMKDDSVSNDTVCHSSSKVPCRSNEHAKDEVKTTSEGSKDCLNVVAGHDTPSTISEFSKQSTSDAEPVQS |
| GgWRKY20 | MK511258 | MELVSERSRHEAGGKGAKEGKRSESSGDDENLPEQENIVAQEPPMTNIERSSVDAGPNASSPKKEEVDELEKTKVEMGGVMEENERLKMRLNRILNEYRTLQMQFNNIVEQETKNSSDKLNNIIEESDELVSLSLGRLPSNPIAKVSNKQTLKEEEEHEKEGLALGLDCKFETSKSGSTTEHLPATNNPSPTNSSELVPKEEAGETWPPSKTTNKAAIRSVAAEDEVISQQNPAKKARVCVRARCDTPTLNDGCQWRKYGQKISKGNPCPRAYYRCTVATSCPVRKQVQRCAQDMSILITTYEGTHNHPLPLSATAMASTTSAAASMLLSGSSTSSSHPGLTTIPSTYTTTTASAGLQYGMNFSLSDASINKSKQFYHLSHPSLSSSANSHPTITLDLTSNPPSSSSSPFARFTSDYNNPSRYPSSTTSLNFSSSESNNAVSWSNGMNGFLSYSAAPQQPYNNRNNILSTININPVRQQPMENNNILSTININPVRQQPMENNNIYSSYMQRNNSNIPIQSQQQSLPDSTIAAATKAITADPSFQSALAAALTSIIGTKSTTATTTQGNHHQQGSTSVGENLGQKMKWGELFSSSSALPAASTASKVSSGCASSFLNKAPSAATTQTGSLMCLQQPLPFPGPKSASASPGDNRDTTN |
| GgWRKY21 | MK511259 | MEAVSNMSSTSCGPEIEEKRITSIFCEDDFRTQKVRKEDRVKSAKAEMGEVKEENERLKMMLERVEKDYHSLQLRFFDILHKDVSKKGVAADSSTTSHDETEEPAELVSLCLGRSPKESNKKDAARTGDSSNKPKEKVEDMEVNLTLGLDSKYLLSMELLSDLSPMNSSELEQKEAEPEGTTTLSTNKSAKVINVNDESSDQMPAKRARVSVRAKCETPTMNDGCQWRKYGQKIAKGNPCPRAYYRCTVAPACPVRKQVQRCAEDLSILITTYEGTHNHPLQVSATAMASVTSAAASMMLSGSSTSQHYAGHHSSASFGTNSPTVLNGLSFSRQYDQEYSRAKELFLPPPNHASHLFPIVTLDLTSSASFSSSQTHVHNNLPSNIASSTRFSPPSLSFCSPPEPNFTPSIWAKGFPNKTQTRPIIQGNHFQEHLQCMMTNQTPPSREALAETITKAISTDPSLRSVIAAAVSSIVGTGSTSGDQAHEEIRENNNILGPGGLNLRLGEHIPQLVSSSNQNGKGQCLTGYFKRLSPTSSQARNFMLLQPSSLPFSLSKSSTSKPPSIVNHFNHYDPDMNTHH |
| GgWRKY22 | MK511260 | MVSSEESADHNVSSDKVQQRVSPQRDITLSQGNHDTEIHLSNPEGARSIPSIVAKNEVKDSDATACALQSDQEGRAHSLPREKHLQIPDSLSHELPPSQSGQDSPSIIREKVSKDGYNWRKYGQKHVKGNEFIRSYYKCTHPNCQAKKQLQQSHNGQITDSICIGRHSHPRPQSNTIVPDDRVLPVVEKEPHKPSLANVEDKSSVDHGCMPQQIKPLNSLPPSKVSGADKLKAAHVQLTAKYEVHNLEDPESKRLKKDSSNVDVTGVDMSTCESRVAVHTSSEVDFVNDGYRWRKYGQKMVKGNANPRYMLAFHFKGSRIFISTHVVLECVHIGPDYLILFHMG |
| GgWRKY23 | MK511261 | MSTCESRVAVHTSSEVDFVNDGYRWRKYGQKMVKGNANPRSYYRCSNPGCPVKKHVERASHDSKIVITTYEGQHDHEIPSGRSVTHNAATNTHTMDIDGKLGTKSGGNTVCVDTGERNCLDSKSRLNKRPNEESITNSEAGDMAEFRVISLRNEGPENKLSQPEQQNEDSDMKDDSVSNDTVCHSSSKVPCRSNEHAKDEVKTTSEGSKDCLNVVAGHDTPSTISEFSKQSTSDAEPVQS |
| GgWRKY24 | MK511262 | MKSKFSNPYINELQEDTDVSQENIAESPPNSASTAFNIDGMVTSPSTSSSSAKRSRRAIQKRVVQIPIKEIEGPRLKGESNTPPSDSWAWRKYGQKPIKGSPYPRGYYRCSSSKGCPARKQVERSRVDPTMLVVTYSSDHNHPWPLSRNHHGSARPTTTVKKPEPDPVEPDEKFSELCGDVELGWFGEMETASSAILESPIMAAEFDADVASVLLPMGEEEESLFADLGELPECSAVFRHGLLDDRRRFTPPWCGTTT |
| GgWRKY25 | MK511263 | MDTSVTSSAKRRGIKRRVVEIPMKEVESSNTPPSDSWAWRKYGQKPIKGSPYPRGYYRCSSSKSCPARKQVERSHVDPTVLLVTYSSDHNHPWPLSRNHHGSARPTTTVKKPEPDPVEPDEKFSELCGDVELGWFGEMETASSAILESPIMAAEFDADVASVLLPMGEEEESLFADLGELPECSAVFRHGLLDDRRRFTPPWCGTTT |
| GgWRKY26 | MK511264 | MGFVICVMIIRRGIKRRVVEIPMKEVESSNTPPSDSWAWRKYGQKPIKGSPYPRGYYRCSSSKSCPARKQVERSHVDPTVLLVTYSSDHNHPWPLSRNHHGSARPTTTVKKPEPDPVEPDEKFSELCGDVELGWFGEMETASSAILESPIMAAEFDADVASVLLPMGEEEESLFADLGELPECSAVFRHGLLDDRRRFTPPWCGTTT |
| GgWRKY27 | MK511265 | MCSIFVPLITNMDNYQGDLTDIIRATATATAGTSSSPSEQQPPAHAAPPQQQHHHHDIDHWHHHHHHHQFSSHDPMMSGSSFNPFGDPFSTMRDPFLQELDIMPPITATTVSSSNSYFNNNTTSTTSTNNNPTSSSCSVFAHNKVVVDHSSRPPCKNIFSNMIQISPNNAKLLPVLPYHDSSSSSSPAVAMAPSPRAIKPSAVVSPNMTINASSKDSLLVQQDNTCTAAGGGGGGVQISSPRNPGLKRRKNQAKKVVCIPAPAAANSRQTGEVVPSDLWAWRKYGQKPIKGSPYPRGYYRCSSSKGCSARKQVERSRTDPNMLVITYTSEHNHPWPTQRNALAGSTRSQPSKNNNNNDNASSSSKNSETSQKGTTVTTTKPKEEDQQQQQEISNNSTDIIGCSNINSPLVNNSSSANNSVKEEMEEDIEKVQLEMEEGISEFSDGVVLPYKPSMMNMDQNSNIINQSHEDFFAELGEIETDPLNLLFTQDHFADPNNPQRESNVKALDDPFHLFDWSGEDHTNNTNNSFEEPNIKRRL |
| GgWRKY28 | MK511266 | MVSSEESADHNVSSDKVQQRVSPQRDITLSQGNHDTEIHLSNPEGARSIPSIVAKNEVKDSDATACALQSDQEGRAHSLPREKHLQIPDSLSHELPPSQSGQDSPSIIREKVSKDGYNWRKYGQKHVKGNEFIRSYYKCTHPNCQAKKQLQQSHNGQITDSICIGRHSHPRPQSNTIVPDDRVLPVVEKEPHKPSLANVEGEK |
| GgWRKY29 | MK511267 | MDKYQGDLTDIIRASGAYGSCSSSTVTSSPEAASFLSLHHWQWQLSSVPEDPSGNFGDPFSNMRDPFLVPEQLDMPAVGSAAYLINSASTTTSAEIISSSGGLEEAAACFGGSSSSNNNTCVLAQKKINLLDQGRPNCNSSILPNMMQISSINNDSAAYNKLPISPGDDHMASLSSRVVKPSAKMIHDEKASKDHCLVTNTREGVLQISSPVIRNPGLKRRKSQGKKSICIPAPADLNSRQSGEIVPSDLWAWRKYGQKPIKGSPYPRGYYRCSRGYYRCSSSKGCPARKQVERSMTDPKMLVITYSSDHNHQWPTQRNALAGSTRSSQPSKTADSKKSETTDHIQPSSTTKPEEEEQQQEISNSDSNVYPPCSCEGRDH |
| GgWRKY30 | MK511268 | MEETSSKRKHVSLINELIQGKELAKQLSDHLVTSYDPSISHETNELLIEKILSTYEKALTMLNCGSNVGDTKTISGNMMMDSHCSSTNESPRSEVMDLEFKHKALFKKRKTMPRWTEQVKICSETGIEGSLDDGYSWRKYGQKGILGAKFPRGYYRCTHRNAQGCLATKQVQRSDEDPTIIEVTYRGRHTCMQAKHLNKESPSKTKMGLEENQHHNDQKNQLVQQEKMEQTPEAIFKFKAELEVKTEDLETKEDIFPWFCFPSPSIGSENDFMESFSPAFISPATSESNLFCLSAYPLGSTGLCQNPQTSESDITDTVSAPTSVTNSPLLDLDILLNKGDFDTDFPFNTPEYFSS |
| GgWRKY31 | MK511269 | MAEVEEHNNKRTFEAEQNEEQRNENAEVEAEAPHRVTDSAQLNSETLVVSSPLPCHHTAHLQESSTAHKGGKDESKEPAGPPDKETIVEVAVEGPQMQTKNQFQVSVCSPPLSELSPTSVTQSLSSAPSPTVPEHRLSPPKAISEHVLEVDKRTPGGKTLSSVSVARASDGYNWRKYGQKQVKSPTGSRSYYRCTHSDCSAKKIECCDHSGHVMEIVYKSQHSHDPPHKTNSTRESKSLPSNEPNVENTIPNQSTRVLNDSDPSPSPKNPLQEEPCSADKKRQSSPNGENGKVNLKEEHDEPEPKRRMKKGDLTSLDSPGKPGKKQKFVVHAAGDVGISGDGYRWRKYGQKMVKGNSHPRNYYRCTSAGCPVRKHIETAVDNSDAVIITYKGVHDHDMPVPKKRHGPPSAPLVAAAAPASMNNLQVKKPDSPQNQKTPTQWSVDTEGELTGEALDLGGEKAIESARTLLSIGFEIKPC |
| GgWRKY32 | MK511270 | MSKWTEQVKVCSRTALEGSLDDGYSWRKYGQKEILGAKFPRGYYRCSHRNVQGCLATKQVQKSDEDPMIYVITYTGRHTCTQASHYLNKAIQSKTKLCLGENKRQKNQPQEEKIEQPQDKIFSFGSGPEVEVENLDNKEDIFPSFIFPSPSTGSENEDDDNNNNN |
| GgWRKY33 | MK511271 | MTPYSSSEFILSDYLMLDDNCVDHHQESDQSQSTESSSVTFNDASHGFDGAISSNNNMLVKCKNGNKRNKTELERPRVTFRTRSELEIMDDGYKWRKYGKKAVKNSPNLRNYYKCSSEGCSVKKRVERDRDDSSYVITTYDGVHNHESPFTSHYSQISFVQPDAA |
| GgWRKY34 | MK511272 | MENLAYGSRKRVIEELAKGREIAIQLRRVLNEGGGDNNNNNNSNGSSSSLVAVAAATPFAEKLVKEVLMSFTNSLSFLNDPACESHEVVSDLQQIRDTNSKSEDSLESNCKSSIVSSKERRGCYKRRKTSQTWEKESEFPVEDGHQWRKYGQKMILNAQYPRSYYRCTHKYDQGCRATKQVQRIQEDPPLHKTTYCGHHSCRNLQNPEIIVDSVSPSHDHSSMFLSFDNSLPTPSKQDCPFLSLSSSSVKRECKEEEEIAIPDLPPSSSSLNNDYLIPSELTFDDDSQRHHALLSTTLDDVSLYDSAELDDVLGGFLNEIR |
| GgWRKY35 | MK511273 | MANEKDRYYNPFYDHNHHDDELNNNNHSNNIPFFTSQQNFEGLLMDPSHTTSFSDYLHGSSMDYNTLSRAFDLSCSSSSHEVKPSNNSTTAAAGDSPLVGRNINSENQSTTLNSSVSSSSNEEAEEEDSITKSNKKDKQQPTKGCTEDGDHDDQKSKKENNKAKNKEKKPREPRFAFLTKSEIDNLEDGYRWRKYGQKAVKNSPYPRSYYRCTSQKCGVKKRVERSYQDPSIVMTTYEGQHNHHCPATLRGNAMLMSSPIPSLFAGSTSSSMGPTSLVPQDFLSQLLIPSYNSSTQNDHQIMLHQNLIPQQQQQQQFQLHHHDYGLLQDLLPSSFPAKNNDP |
| GgWRKY36 | MK511274 | MFVVLERFRNNKAKKKEKKPREPRFAFLTKSEIDNLEDGYRWRKYGQKAVKNSPYPRSYYRCTSQKCGVKKRVERSYQDPSIVMTTYEGQHNHHCPATLRGNAMLMSSPIPSLFAGSTSSSMGPTSLVPQDFLSQLLIPSYNSSTQNDHQIMLHQNLIPQQQQQQQFQLHHHDYGLLQDLLPSSFPAKNNDP |
| GgWRKY37 | MK511275 | MLEVLSSKCTKLESHLQEINKKAEQKGILSNQVGSVPSLDASKRARLEFPTAKKPLQIFFRTHPNDDSLIIKDGYQWRKYGQKVTKDNDSPRAYFRCSMAPSCPAKKKVQKCIHDRSIIVATYDGEHNHSVPHEFGPSSSTPKGSSIIANELPPTPNEKEAINIDSDIPGWAQTSTEICENVMQQCGYGRHINIEEYVGSLIKDPDFTTALAEAVARTITDQHKQQQDLNLNLDLPEE |
| GgWRKY38 | MK511276 | MDLGLATNASDIDEPSLSSSVGRSTQDRSKSPANNIEVGSKMARKNGNGGDELAPVFDHDHKKDRGIEREDSPQDQVLASANNNVPKFSPPRNIDQAAEATMRKARVSVRARSEAPMITDGCQWRKYGQKMAKGNPCPRAYYRCTMAAGCPVRKQVQRCAEDRTILITTYEGNHNHPLPPAAMAMAQTTSSAARMLLSGSMSSADGLMNANFLTRTLLPCSSSMATISASAPFPTVTLDLTQSPNPLQFPKPQTQFQIPFSQNFANSTASSLLPQIFGQALYNQSKFSGLQMSQDVAADPSQLGNQSHQQAQPQQLADTVSAATAAIAADPNFTAALAAAITSIIGAAQTNNNNSSSSNNNGNTTANNSSNGNITSSNNNSNLKQ |
| GgWRKY39 | MK511277 | MVRESGLSMDSESDPIGSSSSSLHNPIVLNSFTQDINNHPKWKLSPNYNNNNMEVTVSTTTKGSSSSPTRTTTMPFQVNLSCSTDNNHTSHDTRTEMDFFKDKNINDVDDHKVVASASVPDNNDHSPTPPMLEFRVNTGLNLLTTNTSSDKSMVDDEISPNSEEKRTKNELAILKSELERMKTENHQLREMLEQVNSNYNALQMHLASLMQDQKAEESEEQQVFDGKKRSENGGAVLVPRQFMDLGLATNAETDEPSSSSMVRSQDPSGSPGGNNNNMEVASKELVKNGNVVSDEGLVYDEEKKKEFGRGGINNEREDSPSGHALAADKVPRFSPPKNVDQAEATMRKARVSVRARSEAPMITDGCQWRKYGQKMAKGNPCPRAYYRCTMAAGCPVRKQVQRCAEDRTVLITTYEGNHNHPLPPAAMAMAQTTSSAARMLLSGSMSSTDGLMNANFLTRTLLPCSSSMATISASAPFPTVTLDLTQSPNPLQFPRPPNQLQIPFPAGIPQNFANSLMPQIFGQALYNQSKFSGLQMSQDAVTDPSQLGNHSQQAQPNIADTVSAAIAADPNFTAALAAAITSIIGGAQPNNNSATNNNNGNMTANNSNGNGNGNVTSSNNNSNGKQ |
| GgWRKY40 | MK511278 | MDEKVETLQKELQHVRKENNTLRLMLEVLSSKCTKLESHLQEINKKAEQKGILSNQVGSVPSLDASKRARLEFPTAKKPLQIFFRTHPNDDSLIIKDGYQWRKYGQKVTKDNDSPRAYFRCSMAPSCPAKKKVQKCIHDRSIIVATYDGEHNHSVPHEFGPSSSTPKGSSIIANELPPTPNEKEAINIDSDIPGWAQTSTEICENVMQQCGYGRHINIEEYVGSLIKDPDFTTALAEAVARTITDQHKQQQDLNLNLDLPEE |
| GgWRKY41 | MK511279 | MAEHSSEALPAGATELHRKGNDSESNDGNQEEKEKAEEIKERVGESPSATELQRGDLSSSNEHTMVPNSETLGAVPSSVVQHRSSDLQGSPTALNSDGRAESKETVGPPEKEIIEREAAEAPPTQTGNQLQVSVCSTPFSELTPNSVTQSLSSVASPTIRKQKMSPPKTNNMHLEEVDRKKPSGGKALSPVSVARTSAPDGYNWRKYGQKQVKSPTGSRSYYRCTHSNCCAKKVECCDHSGHVIEIVYKSEHSHDPPQKTNSIRESKHVSSNEPTAEKSVSEKPIRVVKDPDPSISSKPLQEAPCSADKKRQNSSNISDNGKVILKEEHLDEPEPKRRKEKGDLTDSDSLVKLEKKPKLVVHAAGDVGISGDGYRWRKYGQKMVKGNPHPRCIPIYSLLSFFLFVLNMNLIIFPW |
| GgWRKY42 | MK511280 | MAEHSSEALPAGATELHRKGNDSESNDGNQEEKEKAEEIKERVGESPSATELQRGDLSSSNEHTMVPNSETLGAVPSSVVQHRSSDLQGSPTALNSDGRAESKETVGPPEKEIIEREAAEAPPTQTGNQLQVSVCSTPFSELTPNSVTQSLSSVASPTIRKQKMSPPKTNNMHLEEVDRKKPSGGKALSPVSVARTSAPDGYNWRKYGQKQVKSPTGSRSYYRCTHSNCCAKKVECCDHSGHVIEIVYKSEHSHDPPQKTNSIRESKHVSSNEPTAEKSVSEKPIRVVKDPDPSISSKPLQEAPCSADKKRQNSSNISDNGKVILKEEHLDEPEPKRRQVRI |
| GgWRKY43 | MK511281 | MQTKNQFQVSVCSPPLSELSPTSVTQSLSSAPSPTVPEHRLSPPKAISEHVLEVDKRTPGGKTLSSVSVARASDGYNWRKYGQKQVKSPTGSRSYYRCTHSDCSAKKIECCDHSGHVMEIVYKSQHSHDPPHKTNSTRESKSLPSNEPNVENTIPNQSTRVLNDSDPSPSPKNPLQEEPCSADKKRQSSPNGENGKVNLKEEHDEPEPKRRMKKGDLTSLDSPGKPGKKQKFVVHAAGDVGISGDGYRWRKYGQKMVKGNSHPRNYYRCTSAGCPVRKHIETAVDNSDAVIITYKGVHDHDMPVPKKRHGPPSAPLVAAAAPASMNNLQVKKPDSPQNQKTPTQWSVDTEGELTGEALDLGGEKAIESARTLLSIGFEIKPC |
| GgWRKY44 | MK511282 | MEGKVALKKLLQVQERPYDNNSDALKGTEEQSIKHTSGSTDHLLSLSLNNSELPQKKQEQPLTQLGWLQTKFEEVKKENQILRSMLNQITDHYAVLQSRLLLAMQQQQLPSSPTNNDDLQKDNMRPEDMEKPVLPATWLQRLNSGDPSPADGSSTTKAFAYAENIEKNIDRNLTSCAYKAVEGKINKQTIISQQEDGASEAASCRKARVSIRAPSDFSLMGDGCRWRKYGQKIAKGNPCPRAYYRCNMGTECPVRKQVQRCAVDETVIIITYEGNHNHSLPPAARSMASTTSAALSMFLSGSTTSTSTLSNYSGLFSSSSTPPGLATFYPSASCPTVTLDLTQPSIDLLKFQRAISSNHCHHFPTLPLHGYNSTQQYSDSKPSSTMIPSEKTSLALVDVVSAAIIKDPSIKAALDAAVSSLITGDTLNINN |
| GgWRKY45 | MK511283 | MYHRSRFNTQPLYVADPDEPEPISEPGPASPSSGEDTKTEAPSPQKRREMKKRVVTIPIGDVDGSKSKGETYPPSDSWAWRKYGQKPIKGSPYPRGYYRCSSSKGCPARKQVERSRVDPTKLIVTYAYEHNHSLPLPKSHSSAASAATGDGAAASPDESAARFQPEELTVFATQSDLELAGDSAVLLCHHHHADFGWFDDVASTGVLESPICGEVEDVTMPMREEDESLFADLGELPECSVVFRRRNIPSASAIQCGGITG |
| GgWRKY46 | MK511284 | MAEHSSEALPAGATELHRKGNDSESNDGNQEEKEKAEEIKERVGESPSATELQRGDLSSSNEHTMVPNSETLGAVPSSVVQHRSSGTLRFQWSVLVCVCVLFHPSDFFYFNFLVNCVVDVTDLQGSPTALNSDGRAESKETVGPPEKEIIEREAAEAPPTQTGNQLQVSVCSTPFSELTPNSVTQSLSSVASPTIRKQKMSPPKTNNMHLEEVDRKKPSGGKALSPVSVARTSAPDGYNWRKYGQKQVKSPTGSRSYYRCTHSNCCAKKVECCDHSGHVIEIVYKSEHSHDPPQKTNSIRESKHVSSNEPTAEKSVSEKPIRVVKDPDPSISSKPLQEAPCSADKKRQNSSNISDNGKVILKEEHLDEPEPKRRKEKGDLTDSDSLVKLEKKPKLVVHAAGDVGISGDGYRWRKYGQKMVKGNPHPRCIPIYSLLSFFLFVLNMNLIIFPW |
| GgWRKY47 | MK511285 | MAEHSSEALPAGATELHRKGNDSESNDGNQEEKEKAEEIKERVGESPSATELQRGDLSSSNEHTMVPNSETLGAVPSSVVQHRSSDLQGSPTALNSDGRAESKETVGPPEKEIIEREAAEAPPTQTGNQLQVSVCSTPFSELTPNSVTQSLSSVASPTIRKQKMSPPKTNNMHLEEVDRKKPSGGKALSPVSVARTSAPDGYNWRKYGQKQVKSPTGSRSYYRCTHSNCCAKKVECCDHSGHVIEIVYKSEHSHDPPQKTNSIRESKHVSSNEPTAEKSVSEKPIRVVKDPDPSISSKPLQEAPCSADKKRQNSSNISDNGKVILKEEHLDEPEPKRRKEKGDLTDSDSLVKLEKKPKLVVHAAGDVGISGDGYRWRKYGQKMVKGNPHPRNYYRCTSAGCPVRKHIETAVDNSDAVIITYKGVHDHDMPVPKKRHGPPSAPLVAAAAPASMNNLQVKKPDSPQNQKTPTQWSVDTEGELTGEALDLGGEKAIESARTLLSIGFEIKPC |
| GgWRKY48 | MK511286 | MELGLSEVDILDDGYRWRKYGQKVVRGNPNPRSYYKCTNVGCPVRKHVERASHDLKAVITTYEGKHNHDVPAARSSSHDMVGPSAASGSHTRIKLEESDTISLDLGMGLSSNRSNGQGKMLLSEYGDSQTHTSSSNSSFKFVNHTTNPSAPVYYGVLSNGSNPYGTSRENSRSDGRPSLNHSSYPCSQNMGRILMGP |
| GgWRKY49 | MK511287 | MYGQDSTPELSPGATNDDNIEGAGFVSNRTNDEIDDDDPFSKRRRMELGNADFIPVVKPIREPRVVVQTLSEVDILDDGYRWRKYGQKVVRGNPNPRSYYKCTNVGCPVRKHVERASHDLKAVITTYEGKHNHDVPAARSSSHDMVGPSAASGSHTRIKLEESDTISLDLGMGLSSNRSNGQGKMLLSEYGDSQTHTSSSNSSFKFVNHTTNPSAPVYYGVLSNGSNPYGTSRENSRSDGRPSLNHSSYPCSQNMGRILMGP |
| GgWRKY50 | MK511288 | MVSSGASAPVDADLDEFNHKGNTATGPQTSHVEVRGSGLSVAAEKVSNDGYNWRKYGQKLVKGSEFPRSYYKCTYPNCEVKKLFERSHDGQITEIIYKGTHDHPKPQPSNRYSVGAVMSMQGERSDKAFLAGRDGNTSQMLLEFSLLPFYYYLLQ |
| GgWRKY51 | MK511289 | MEDKHRAAHDPATAGEFAGESSWTLNADYSDAAYYFSGERERSMFGDFGWNHVPDRIAATDEGLWPSMTGSIEPPATAAASSYRSNNQSVSSSSSEDPPEKSTVSDEKPPEIPSKSKKKGQKRIRQPRFAFMTKSEVDHLEDGYRWRKYGQKAVKNSPFPRSYYRCTNSKCTVKKRVERSSEDPTIVITTYEGRHCHHTVGFPRGGIISHEAAAFTTGQLAPTSMSQFYYPIIQSPRSDINTCSSNIISQPCQAAQDEAGRSSRSTTTPASDAPPPQPAPTDEGLLGDIVPLGMRNR |
| GgWRKY52 | MK511290 | MLTEMCENYNTLRNNLMEYMRKNPDNKELSPSRKRKSESSNNNNSNVIGVNGNNSESSSSDEESYKKVPREETIKAKVSKVYFRTEASDSSLIVKDGYQWRKYGQKVTRDNPCPRAYFKCSLAPSCPVKKKVQRSVDDQSVLVATYEGEHNHPLPSQMEATQGSNRSVTLGSAPLSSSAPTVTLDWTKSKPTNESKNMIINPKIDSPEVPQILVEHMATSLTKDPNFRAALVAAISGKVLHKS |
| GgWRKY53 | MK511291 | MDCSSWINTSLDLNINPHRVHEEVPKMEVESKLFSLGMTKFSVKEESTDELEEELKRVTAENKKLVEMLSVVCENYNTLRSHLMEYMKRNPEKELSPSSKKRKSESSNNNNSNLMGINNGNSESSSTDEEESCKKPREETIKAKISRVYVRTEASDTGLIVKDGYQWRKYGQKVTRDNPCPRAYFKCSFAPSCPVKKKVQRSVDDQSVLVATYEGEHNHPQPSRMEATSGSGRGMSQGSSVPCSASLISSAPTVVTLDLTNSKSSKGSKNTEPRKDSPKVPQNLVEQMATSLTTDPNFRAALVAAISGRLMHKN |
| GgWRKY54 | MK511292 | MEVESKLFSLGMTKFSVKEESTDELEEELKRVTAENKKLVEMLSVVCENYNTLRSHLMEYMKRNPEKELSPSSKKRKSESSNNNNSNLMGINNGNSESSSTDEEESCKKPREETIKAKISRVYVRTEASDTGLIVKDGYQWRKYGQKVTRDNPCPRAYFKCSFAPSCPVKKKVQRSVDDQSVLVATYEGEHNHPQPSRMEATSGSGRGMSQGSSVPCSASLISSAPTVVTLDLTNSKSSKGSKNTEPRKDSPKVPQNLVEQMATSLTTDPNFRAALVAAISGRLMHKN |
| GgWRKY55 | MK511293 | MESTCVDTSLNLNIVPSLHMDEVLVEELHRLSCENKRLNETLNHMCENYQAMQNQLSQLMNKNFEHPTTQQSRKRKADQGEIGCINKFGGIRGNVVNTVDQCSTITEEESLIKRPMDIISPKVYKVLVKTEATNNSLYVMDGYQWRKYGQKVTRDNPSPRAYFRCSYAPSCPVKKKVQKSVEDPTILVATYEGEHNHGLQKGEMSLVSRQSDQESPVGFVPPISSPTPIIQSTSPTVTLDLVEENINGQKSSIQQFLVQQMATSLTRDPNFTAALATAISGRILDQSSNKAKW |
| GgWRKY56 | MK511294 | MCENYQAMQNQLSQLMNKNFEHPTTQQSRKRKADQGEIGCINKFGGIRGNVVNTVDQCSTITEEESLIKRPMDIISPKVYKVLVKTEATNNSLYVMDGYQWRKYGQKVTRDNPSPRAYFRCSYAPSCPVKKKVQKSVEDPTILVATYEGEHNHGLQKGEMSLVSRQSDQESPVGFVPPISSPTPIIQSTSPTVTLDLVEENINGQKSSIQQFLVQQMATSLTRDPNFTAALATAISGRILDQSSNKAKW |
| GgWRKY57 | MK511295 | MSHQQALAQVTAQAVLAQSHMHMQADYHQSSVTAPTEPVVEQPSFTLNEASEQQVVASAVSEPRNAQLETSELAHADKKYQPSSVAIDKPADDGYNWRKYGQKQVKGSEYPRSYYKCTHLNCPVKKKVERAPDGHITEIIYKGQHNHEKPQANRRVKDNTELNGNANIQSKSDSNSQSWIGNQNKVSESMPDCSMPESDQTSNQGAPRPLPGSSESEEVGDVDNREEGDDGEPNPKRRQVFLHVNPSPYSYAFFFMIYLC |
| GgWRKY58 | MK511296 | MHADKSSVDHGCMPQQIKPLNSLPPSKVSGADKLKAAHVQLTAKYEVHNLEDPESKRLKKDSSNVDVTGVDMSTCESRVAVHTSSEVDFVNDGYRWRKYGQKMVKGNANPRSYYRCSNPGCPVKKHVERASHDSKIVITTYEGQHDHEIPSGRSVTHNAATNTHTMDIDGKLGTKSGGNTVCVDTGERNCLDSKSRLNKRPNEESITNSEAGDMAEFRVISLRNEGPENKLSQPEQQNEDSDMKDDSVSNDTVCHSSSKVPCRSNEHAKDEVKTTSEGSKDCLNVVAGHDTPSTISEFSKQSTSDAEPVQS |
| GgWRKY59 | MK511297 | MDAGEAHSGSELRRRGPGEAESEHEDPNRTGGYRPDSGADGAAASGTTVGARYKLMSPAKLPISRSPVLTIPPGLSPTAFLESPVLLSNMKVEPSPTTGSLPRLQQTAHGSVTSATSAAFPVTTVCFNTNTLDDGKSSFFEFKPHSRSNMVPAELDNRACEKSTQIDGQGKAQSFASSPLVKSEIAGPSNEISLSSPVQMVSSGASAHVEVDLDKLNSGGNIATGLQVSQVEGRGSGLSVAAERSSDDGYNWRKYGQKHVKGSEFPRSYYKCTHPNCEVKKLFERSHDGQITEIVYKGTHDHPKPQPNRRYSGGNIMSMQEERSDKASLTSRDGNAR |
| GgWRKY60 | MK511298 | MDLGNADITPVVKPIREPRVVVQTLSEVDILDDGYRWRKYGQKVVRGNPNPRSYYKCTNAGCPVRKHVERASHDPKAVITTYEGKHNHDVPTARNSSHDMAGPAAAGGQTRIRQEESDTISLDLGMGLSSNRSNGQGKMLLSEYGDSQTHTSSSNSSFKFVNHTTNPSAPVYYGVLSNGSNPYGTSRENSRSDGRPSLNHSSYPCSQNMGRILMGP |
| GgWRKY61 | MK511299 | MLTEMCENYNTLRNNLMEYMRKNPDNKELSPSRKRKSESSNNNNSNVIGVNGNNSESSSSDEESYKKVPREETIKAKVSKVYFRTEASDSSLIVKDGYQWRKYGQKVTRDNPCPRAYFKCSLAPSCPVKKKVQRSVDDQSVLVATYEGEHNHPLPSQMEATQGSNRSVTLGSAPLSSSAPTVTLDWTKSKPTNESKNMIINPKIDSPEVPQILVEHMATSLTKDPNFRAALVAAISGKVLHKS |
| GgWRKY62 | MK511300 | MDEKVETLQKELQHVRKENNTLRLMLEVLSSKCTKLESHLQEINKKAEQKGILSNQVGSVPSLDASKRARLEFPTAKKPLQIFFRTHPNDDSLIIKDGYQWRKYGQKVTKDNDSPRAYFRCSMAPSCPAKKKTQQVDIRLHN |
| GgWRKY63 | MK511301 | MEEVEEANRAAVESCHRVISMLCHPQDQVLQFRNLMVETGEAVVRFKKVVSLLSSNGLGHARVRKLNNKLKTPFSQSILLDNPNCRTNHHQSTNMQFPRTSFPDNSIQELASSGIRNSLFLGNPSLELSSNGKSPLHLTQEPSSKTHYNILQQQQQIQQQQRLLLQQQQHQQMKHQAEMMFRRNNNNSVINLNFDSSSCTPSISSTRSFISSLSIDGSVANLDGGSGFRLVGGAAAHSSDQNSQQHKRKCSARGDEGSVKCGSSSARCHCSKKRKHRVKRSIKVPAISNKLADIPPDDYSWRKYGQKPIKGSPHPRGYYKCSSMRGCPARKHVERCLEEPTMLIVTYEGDHNHPKLPTQSANA |
| GgWRKY64 | MK511302 | MAVELMMGGYSRNESSSFTATAEENAVQEAASGLESVEKLIRLLSKARQHYNNNNHHSSSPSSSAASPSNPNPPPSSMEIERDCRAVADVAVSKFKRVISLLDRTRTGHARFRRAPLPQPPQQNQTPQQTQQPYSEPIVFKATPLQQIPPTLHHNHHHNHTIERKDSSKTLNFSYSSAANSFISSLTGDADAKQPSSPPLPLPAGAFQITNLPQASSAGKPPLSSSSLKRKCSSETLGSGKCGSSSSRCHCSKKRKLRLKRVVRVPAISLKMADIPPDDYSWRKYGQKPIKGSPHPRGYYKCSSVRGCPARKHVERALDDPSMLVVTYEGEHNHSLSAADASNLILESS |
| GgWRKY65 | MK511303 | MEEDWDLHAVVRGCSAVTSTTAVPSVSSSSSGFGTSCSLLQPEASSSCGFSIFDGEQQKGQIFSPSAAYPFEARGSIQELHELCKPFFPKSHPLPLQTSSLSSSFSYSSVAAPPKSAQTQEKQQQQQRIKQPHQHQAGSVTTPRSKRRKNQLKKVCQVPVESLSSDIWAWRKYGQKPIKGSPYPRGYYRCSSSKGCLARKQVERNRSDPTMFIVTYTAEHNHPAPTHRNSLAGSTRQKPLTPQNATTTTGSDSDKALTKPSSPANSGAEEDQEVPTQGEKSDQSREEEKEDDEELLGEDEFGGLSDMVLSDDFFESFDELSQLSAVVTGGECFVDPFSAIAIPAWSAATAASGS |
| GgWRKY66 | MK511304 | MEEVEQANRAAVESCHRVLSMLSQPRDQVQHRNLMVETGEAVMRFKKVVSLLHNGLGHARVRKFKKIQQTPFSQTIFLDNPICKTNHHYSKNLQFPQTSYPENSVQEVGSTIKNSLSLGNPSLELSSSGKSPLQLAQQASSTHYHFFQQQQQQRLLLQQQQQQMKHQAEMMFRRNNSGINLNFDSTSCTPTMSSTRSFISSLSIDGSVANLDGSSFHLIGAPHSSDQNSQQHKRKCSARGDEGSLKCGSSARCHCSKKRKHRVKRSIKVPAISNKLADIPPDDYSWRKYGQKPIKGSPHPRGYYKCSSMRGCPARKHVERCLEEPTMLIVTYEGEHNHPKLPTQSANA |
| GgWRKY67 | MK511305 | MFFFSFRKCKNGNKRNKTELERPRVTFRTRSELEIMDDGYKWRKYGKKAVKNSPNLRNYYKCSSEGCSVKKRVERDRDDSSYVITTYDGVHNHESPFTSHYSQISFVQPDAA |
| GgWRKY68 | MK511306 | MDYYFGNPNPPNPYYGHQYSAVNIAPSSDNFMVSDYLMLDDDDVVNVDHHHHQDSNWSQSTETESSENKAGSSDANIHGFGDETSPNNNNIKRKKGIMKSTAEVSPRRITFRTRSQLEIMDDGYKWRKYGKKAVKNSPNPRNYYKCSGEGCSVKKRVERDREDSSYVLTTYDGVHNHESPCTAYYSPMSLVGALQ |
| GgWRKY69 | MK511307 | MEEVEQANRAAVESCHRVLSMLSQPRDQVQHRNLMVETGEAVMRFKKVVSLLHNGLGHARVRKFKKIQQTPFSQTIFLDNPICKTNHHYSKNLQFPQTSYPENSVQEVGSTIKNSLSLGNPSLELSSSGKSPLQLAQQASSTHYHFFQQQQQQRLLLQQQQQQMKHQAEMMFRRNNSGINLNFDSTSCTPTMSSTRSFISSLSIDGSVANLDGSSFHLIGAPHSSDQNSQQHKRKCSARGDEGSLKCGSSARCHCSKKRKHRVKRSIKVPAISNKLADIPPDDYSWRKYGQKPIKGSPHPRYVQALLLEI |
| GgWRKY70 | MK511308 | MEEVEEANRAAVESCHRVISMLCHPQDQVLQFRNLMVETGEAVVRFKKVVSLLSSNGLGHARVRKLNNKLKTPFSQSILLDNPNCRTNHHQSTNMQFPRTSFPDNSIQELASSGIRNSLFLGNPSLELSSNGKSPLHLTQEPSSKTHYNILQQQQQIQQQQRLLLQQQQHQQMKHQAEMMFRRNNNNSVINLNFDSSSCTPSISSTRSFISSLSIDGSVANLDGGSGFRLVGGAAAHSSDQNSQQHKRKCSARGDEGSVKCGSSSARCHCSKKRKHRVKRSIKVPAISNKLADIPPDDYSWRKYGQKPIKGSPHPRYAGKFLHSFC |
| GgWRKY71 | MK511309 | MAVELITGYGGENDHNRFATTTSTSDDDNAIAVQEAASAGIHSVEKLMNMISQQNHHHQQQEEEANNNVNDGCELIGAVADVAVNRFREVIALLDRPRTGHARFRRAPPTLPQPVPLLQLQQTVESNSNSSKPIVPSSSSFVVQPQQLKTKTEQVSGSAFKVYCPPPPPPPQNNNELAAHGSIKFSVSAANSSFVSTLTGDSENLQQRPCRSSSGFQISHVSLQGSSYMRNKPPLSSNSMKRKCNSVDFLGIKCGSSSSAAQCHCSKKRKLKLKRVIRVPAISAKTADIPADEYSWRKYGQKPIKGSPHPRGYYKCTSVRGCPARKHVERAVDDPNMLVVTYEGDHIHNHSHTTAS |
| GgWRKY72 | MK511310 | MRRNMVVDPVVFPKMRVEEQVAIQEAASAGLKSMEHLIRVLASQASSSCSSSSANQRNRLDLNNLDCTEITDFTVSKFKQVINLLNRTGHARFRRAPSHHPSSPSPVSSQPQPQTQSMTLDFAKPLPLKSNPNPNPNPSTDLSVSQYSMSNKLDTYSISTTTSSFMSSITGDGSVSDGKIGPSILAAGKPPLASSHRKRCHDATLSGKVSSSGHCHCSKRRKSRVKRIIRVAAISSKIADIPADEYSWRKYGQKPIKGSPYPRGYYKCSSVRGCPARKHVERAQDDPNMLIVTYEGEHRHPQTTGATAAAAAFTAQPV |
| GgWRKY73 | MK511311 | MRNKPPLSSNSMKRKCNSVDFLGIKCGSSSSAAQCHCSKKRKLKLKRVIRVPAISAKTADIPADEYSWRKYGQKPIKGSPHPRGYYKCTSVRGCPARKHVERAVDDPNMLVVTYEGDHIHNHSHTTAS |
| GgWRKY74 | MK511312 | MMDIEEAERVVVAKPVASRPTCSTFKSFSELLAGAINASPPIAPSHQTTVSAIRPKTVRFKPAAAMNRPPAGSVSSQADIFGAALSNSSDMSPKPDTKQSLIYKPMAKLVSKTTVSLLANMGNGSTSQQQPRQSMEANLQHPNHEKFGTNMSSSLHQSIPRHAETYMATESCKVVQQNLEEDQKALTSSNTTNGDRPYDDGYNWRKYGQKQVKGSEFPRSYYKCTHPNCPVKKKVERSFDGQIAEIVYKGEHNHSKPQLPKRNTGSGTQQGSDGMVQDIWSNNNQSETNEGRIENNQNDTTGLSVRSAYHVKAPQPNDSALIGGAINAGGGSLENSCGLSGECEEGSKGFEAEEDEPRCKRR |
| GgWRKY75 | MK511313 | MEGERDVNPANYDQLQVSFTNTPQAIHEMGFVQFEENQVLSFLAPASSQSQPISQPQLCQSLNAGAGTTATATAAAATTTGFTHNDLVTRTSWNNELVRTLDPKGVNDENCSGNTSDGNNTWWRSGGTEKSKVKVRRKLREPRFCFQTRSDVDVLDDGYKWRKYGQKVVKNSLHPRSYYRCTHNNCRVKKRVERLSEDCRMVITTYEGRHNHSPCDDSNSSEHECFTSF |
| GgWRKY76 | MK511314 | MEKKEMGVSVSVKTEDVVGGGSSSFPGYSFSSVFDFSEVEKSSLGFMELLGVQDYSPLLDLPQLSTMSVPHHHSTTTGKECSSEVLNQQQPATPNSSSISSASSEAVNDEQNKTSVDQPEEGDEDDEEEKQKTNKQLKAKKTNQKRQREPRFAFMTKSEVDHLEDGYRWRKYGQKAVKNSPFPRSYYRCTSASCNVKKRVERSYTDPSIVVTTYEGQHTHPSPVMHRSGLAGAPIPPGAVSAAGFATNNFGNSVFPGNYLSQYHQHHHHHHQQQLLVNTLSSLGFPNYNDSSSKNAAFTHERQQQLCNNPGATAFLKDHGLLQDVVPSHMLKEE |
| GgWRKY77 | MK511315 | MENEWSWEHSTLINELIQGMEVARRLKEDLRTPYPADTRDLQVQMILSSYEKALQILKWNESTSKLQTMNRAVTLLPESPPLSDDVDGGIQDHQEIKHDSKKRKIVPKWTDQVRVGFESGLEGSHDDGYNWRKYGQKDILGAKYPRSYYRCTFRNTQNCWATKQVQRSDEDPTIFDITYKGKHTCSQGSNAVLPLKSPGKQEKPDRHNNDIRHAQPSQESFTKFRNTLTVRADNLGNEEMACPFTFPSTSFGCTTTQENHSLIPSAFENDSFLSSLFQTQYVSPTTPESNYFSSPTFHMNGFDGIYNKPRSESDITEIISTNTSATNSPIPEFNFSLDPVEIDPNFPFNTPGFFS |
| GgWRKY78 | MK511316 | MPISRSEEASDESTLPENAIHGDDIGQQLVLEGEQKETSHATGVGRTSEDGYNWRKYGQKQVKGSEFPRSYYKCTQPNCPVKKKVERSHDGQITEIIYKSAHNHAKPHPNRRAPAPAPAPSTDEMSEIAEAGETYDKVDADSVWGNIQSAVKDTKHSLEWKADGQERTSSASVVTELSDPISMKRGRSLCMFESEDTPELSSTLASHDGDEDGATQAVVSVEDDAEDVESESKRRYHYSI |
| GgWRKY79 | MK511317 | MSTNQSFEEQDQIPTQMEFIPFPPMAMGFHQSLKAISAITPSLSSEAIDFAETLLATAVQKPREDLTSSLVGAGGGGQQFSLNRSRVINPWAWGEVTADCLMGKRNGGGDDDHDHHHLGVSAIKMKKMKGRRKVREPRFCFKTMSDVDVLDDGYKWRKYGQKVVKNTQHPRSYYRCTQDNCRVKKRVERLAEDPRMVITTYEGRHVHSPSNDLDDSDQSHSQILNNFLW |
| GgWRKY80 | MK511318 | MDLGNADITPVVKPIREPRVVVQTLSEVDILDDGYRWRKYGQKVVRGNPNPRSYYKCTNAGCPVRKHVERASHDPKAVITTYEGKHNHDVPTARNSSHDMAGPAAAGGQTRIRQEESDTISLDLGMGLSSNRSNGQGKMLLSEYGDSQTHTSSSNSSFKFVNHTTNPSAPVYYGVLSNGSNPYGTSRENSRSDGRPSLNHSSYPCSQNMGRILMGP |
| GgWRKY81 | MN625734 | MAVELMMGGYSRNESSSFTATAEENAVQEAASGLESVEKLIRLLSKARQHYNNNNHHSSSPSSSAASPSNPNPPPSSMEIERDCRAVADVAVSKFKRVISLLDRTRTGHARFRRAPLPQPPQQNQTPQQTQQPYSEPIVFKATPLQQIPPTLHHNHHHNHTIERKDSSKTLNFSYSSAANSFISSLTGDADAKQPSSPPLPLPAGAFQITNLPQASSAGKPPLSSSSLKRKCSSETLGSGKCGSSSSRCHCSKKRKLRLKRVVRVPAISLKMADIPPDDYSWRKYGQKPIKGSPHPRYTIISY |
| GgWRKY82 | MN625735 | MRRNMVVDPVVFPKMRVEEQVAIQEAASAGLKSMEHLIRVLASQASSSCSSSSANQRNRLDLNNLDCTEITDFTVSKFKQVINLLNRTGHARFRRAPSHHPSSPSPVSSQPQPQTQSMTLDFAKPLPLKSNPNPNPNPSTDLSVSQYSMSNKLDTYSISTTTSSFMSSITGDGSVSDGKIGPSILAAGKPPLASSHRKRCHDAAPSSSSPCHCSKKRKTRVKRTIRVPAISSKIADIPPDEYTWRKYGQKPIKGSPYPR |
| GgWRKY83 | MK511319 | MSTNQSFEEQDQIPTQMEFIPFPPMAMGFHQSLKAISAITPSLSSEAIDFAETLLATAVQKPREDLTSSLVGAGGGGQQFSLNRSRVINPWAWGEVTADCLMGKRNGGGDDDHDHHHLGVSAIKMKKMKGRRKVREPRFCFKTMSDVDVLDDGYKWRKYGQKVVKNTQHPRYIYLFPGQFNWKACLFKFYTLSLSL |
| GgWRKY84 | MK511320 | MEGERDVNPANYDQLQVSFTNTPQAIHEMGFVQFEENQVLSFLAPASSQSQPISQPQLCQSLNAGAGTTATATAAAATTTGFTHNDLVTRTSWNNELVRTLDPKGVNDENCSGNTSDGNNTWWRSGGTEKSKVKVRRKLREPRFCFQTRSDVDVLDDGYKWRKYGQKVVKNSLHPR |
| GgWRKY85 | MN625736 | MSDEPKELYYQNFNQQGTEGSMYCDQKQLFSSLSAAYDSSQLAFDPSSYMSFTECLQGGGGGMDYNSLIATSFGLSPSSSEVFSSVEGNHHHHQKTPAEAEAGEEDHNMGDGGTTGGGGAAGSETLLNSSISSSSTEAGAEEDSGKRKKDGKVKEEQSSKNGSIKEKKKGEKKQKEPRFAFMTKSEVDHLEDGYRWRKYGQKAVKNSPYPRYKLTTF |
| GgWRKY86 | MN625737 | MIYVLSFNLITRKEKGDLTDSDSLVKLEKKPKLVVHAAGDVGISGDGYRWRKYGQKMVKGNPHPRNYYRCTSAGCPVRKHIETAVDNSDAVIITYKGVHDHDMPVPKKRHGPPSAPLVAAAAPASMNNLQVKKPDSPQNQKTPTQWSVDTEGELTGEALDLGGEKAIESARTLLSIGFEIKPC |
| GgWRKY87 | MN625738 | MDYYFGNPNPPNPYYGHQYSAVNIAPSSDNFMVSDYLMLDDDDVVNVDHHHHQDSNWSQSTETESSENKAGSSDANIHGFGDETSPNNNNIKRKKGIMKSTAEVSPRRITFRTRSQLEIMDDGYKWRKYGKKAVKNSPNPR |

**Table S2 Protein sequence information of *WRKYs* in *G.uralensis***

| **Name** | **Protein Sequence of *Glycyrrhiza uralensis*** |
| --- | --- |
| GuWRKY1 | MAVELMGFPKLDEQKAIQEAASEGLKGMEHLVRTLSHQPTHLNTHLTDLTVSKFKKLISLLNRTGHARFRRAPVHPSTPPVQVQPPPSSSSSLPPTQPQTSTVSPTAQFVSPAPVSVLHAPAIFVPAQSQSLTLDFTKPNDTVLGSNANKSMVELEFSKETTTFSVSSTSSFMSSAITGDGSVSNGKQGSSIFLTPAPAATPTVSGGKPPLSSAPLKKRCHDHLEHSDDVSGSGKCHCIKRRKNRMKRTVRVPAISSKIADIPPDEYSWRKYGQKPIKGSPYPRGYYKCSTVRGCPARKHVERAPDDPAMLIVTYEGEHRHALQAAMQENAAGVVGLVFEST |
| GuWRKY2 | MLVFSYFSHLIISLSLSLSTKSQRLLLSLSLGEAEGTEQNRTESQTHFLSFVSTVMEDDWDLHAVVRGCTMLTSSSSSSSSSTTTTTATASSVSSSGFGTCYQQQPSSASSYSSFSIFPAEQTGQILSLSNPPFEAARGXSIEELHELCKPFFLXSHPPSLHTSSSSFSYNISSAPKSPHTQQKQQHPNKQSHHAGSATTPRSKRRKNQVKKVCQVTAENLSSDIWAWRKYGQKPIKGSPYPRGYYRCSSSKGCLARKQVERNKSDPTMFIVTYTGEHSHPAPTHRNSLAGSTRQKPLTPQTVTDGDDSGNKSLTITKPVSPSTSGAEEEEVVAPLSAKSESIEDMEDLMNDDDDEEEENEFGSSDNMVVTDDFFEGLDELTGFATAATGDCFC |
| GuWRKY3 | METFEEFIAKGPFGCSAFALMSLCLSRQIDLGFETDLLNFFLGCLTVQLMKINLMLSIIAAIICYSLMVLRSKLDSQPEIGTLRMEDHEEEKEELMMTTEVIPNLIHYSHGHQQNQSLRRRVYNDIDGYKWKKYAEKQVEEVEIKKSTIMHEIQLSCEEKVERTMDGKVIETLYKGTHNHYKHMVTMERNSSSEYLYSLLPPSEPSPIDDMPAQLQSFASHDGSEQLDYDAAPESSSVSREKKNESVVVCGWRKVGGSGGERERERVVVGRGGCGERERERERKREERERERERVGW |
| GuWRKY4 | MASPSPTINSNPRSFSSLPPQFMKTSSFTNNNNNNNSSNMMTMDDDHHQNNWTTFEHNHQQVGLDVPKFKSVQPPSLPLSPSPISPSSYFASFSSGFSPTEFFNSPLFLSSPNAFASPTSEAFAGQSFNWKNSSGEDQQQGDKEDEKNYSDFSFQTQTKPTPVFQSSSSMSQVDTWKPILVLSALLNIIGHVFYGLWEQRRSEDGFNWRKYGQKQVKGSENPRSYYKRTSSQPSSSCTNSGISDQSVVTLGNPQMEPVSIQEDSSASVGEEDFEQTSQTSYSGGDEDALGPEAKRWKGDNENDGYSGLGSRTVREPRVVVQTTSEIDILDDGYRWRKYGQKVVKGNPNARSYYKCTAPGCSVRKHVERAANDIKAVITTYEGKHNHDVPSARGSATASYNMNRNSLNNSSVPAPIRPSAPEMRADTARQKKAKIDMLMAADEESVNMLSCPSLILFVIGIAEDADDIKRYAII |
| GuWRKY5 | MEGKVALKKLLQVQERPYDNNSDALKGTVEQSIKHTLGSTDLLSLSLNNSELPQKKQEQPLTQLGWLQTKFEEVKKENQILRSMLNQITDHYAVLQSRSSNYLHHLQIMXTCRQKDNMRQEDMEKSVLPATWLQRLNSGDPSPADGSSTTKAFAYAENIEKNIDRNLTSCAYKAVEGKINKQTIISQQEDGASEAASCRKARVSIRAPSDFSLMGDGCRWRKYGQKIAKGNPCPRAYYRCNMGTECPVRKQVQRCAVDEAVTIITYEGNHNHSLPPAARSMASTTSAALSMFLSGSTTSTSTLSNYSGLFSSSMSSPAGLATFYPSASCPTVTLDLTQPSIDLLKFQRAISSNHCQQFPTLPLHGYNRTQQYSDSKPSSXMIPASEKSLALVDVVSAAIIKDPSIKAALDAAVSSLITGDTLNINN |
| GuWRKY6 | MVGGGNGEGQQQEAPPWTAARPSISIPACAWMESSGVSVSPGPMTLLSSLFGDTDECNKSFSELLAGAAMVEHGEAAPPSGLMFTTLPPHQVSAQAVQNQAEHPFSVSLVPPTSLTQTPATTFTTGQQLVPPSESLDYSSHSEQRLQSSSSSSSSKQVKGCEFPRSYYKCTYPSCPVKKKVERSLEGHVTAIIYRGEHSHQRPHPSKLTKDNLVTSNENSDMQEGISSHPMSEIDPEPSQAMAEVHVSGTSDSEEVGDHEIEEEEKNDEPVPKRRTVSSEPRIIVQTTSEVDLLEDGYRWRKYGQKVVKGNPNPRVSTDPKAVMTTYEGKHNHEVPAAKTNSHTLANNSASQNVIPEMHNFNSRGVGGNEHIEQQPVAYEA |
| GuWRKY7 | MFCRLLLVASDHFVSFLGFNWKTNSGENQQNIKAEDESFSSFSFQTKPHPPLPPTAGTVQTGWSFQDGMSMSMVKTENSSSMQGFTPEKSHKSGLQSDYNNNYPTQAHQTLNRRSDDGYNWRKYGQKQVKGSENPRSYYKCTYPNCPTKKKVEKSLDGQVTEIVYKDGQMDSVATPDNSSISMGDDDFEQSSHKSKSGGGEEYDEDEPDAKKWKHVERASQDLRAVITTYEGKHNHDVPAPRGSSSHNSINRTLPTNIAIRPEGQQPLFNTEMMQNQLASYMNMNQQHDSVFSSRAKEEPADHDSFLESLLR |
| GuWRKY8 | MGDTMKCEQIGLIGELMQGKELAKQLCDHLISASSSSSSHLTNEELIEKILGSYEKALTMLNWKANVGQSKSNNGSPRSEVQDSDQFKHKAVFKKRKTMSKWTEQVKVCSRTALEGSLDDGYSWRKYGQKEILGAKFPRGYYRCSHRNVQGCLATKQVQKSDEDPMIYVITYTGRHTCTQASHYLNKAIQSKTKLCLGENKRQKNQPQEEKIEQPQEKIFSFGSGPEVKVENLDNKEDIFPSFIFPSPSTGSENEDDNNNNNNNNIFSETMIENNFMECFSPAFISPETSESNLFCLSPCHLGPSVQTSKSDFTEIVSDPTTSVANSPIDLDFLVDIDFDTXFLINTPELCY |
| GuWRKY9 | MSPRFRNLLTHPKFWRIVGFVSSLIGTLCYAQSLISFQHVGDEKNHXLKISLYGVVSFTIIFVMLFARKGRFSKSFPLKALVGVLALMLTSLYNFVSDKDNNNKGPNERKTDVFNXISNGSFALMLLSLSXQIDLGCEGELLSFYLGCLTIQLMKINMGFVLVGAAYCYLLNVLHSYADSNSDYNEASCHEGHVAIEIDNAKGEERSEQQLYSILGVEIKSDDERNVIYYYPEDLEVKLVEAMASGFEKVHCYNVFCRWRRQFLLDEELFLKQDLNFEKVQKLSLKYLKDEIENWNKASKVAFRTLFPYERQLCXRVFGFSHDSFADSSFMEICQEPALHLLGFAKSVAKSTTRSSPERLFKMLLVFETLNDVLPDLKSVFSKGXCVVLWNEAVRIWKRLKGTIRGVFMELENMILHQDPSKVVVFGGGLHPVNHYVMNFLFASCQFWKTLELAFEDDDEGPSSSSSTTTIPSACGLLLLSLQVATVLEVLERNLKASSKIYKDSCLGSVFMMNNWRYMVHKVKKCELGKVLGDEWVQRHVAEMGKYLVNYQXRSWDQVLGMLNSVDEDDCDVAAESSMEVLKMKLGEICEMQGTWVVIDEQLREVMKSSLKKILLPAVEKFVARIIQPTWTIVLPNTQDFYEWRKYDEKTILNSTYPRKYYRCIYKNDQGCRARKHVQQIQENPKMYTTSYSGFHTCSVLYIQALKLFARYSITISLS |
| GuWRKY10 | MTERVRERKREKNRTTNTKKSIPKKISQTDPEREKMDIHXCPESGSKKRVIVKELVEGQEAATQLKFLLENPFGGADADSSSLSLAELVANVLRSFTEALSIITSYQSNNNLLNSSGENGSPVTGDSTSDKRKRSMPPARKGGRGCYKRRKSAETWTTVSHTTHDNHAWRKYGQKEILNSEFPRSYFRCTYKYDEGCSARKQVQQTKENPDMYQTMYIGIKKSKATPKAKATTTHDHDLVKKTSSSTCWESYLETEVLPXKKIQDHNISSSTSLAIKQEYKKKEHTPNDHKPLKMESDIDKKMYNSSSTGSDKCLVMDFGKKTVHFCTDLFHFDESQLMGI |
| GuWRKY11 | MENLAYGSRKRVIEELAKGREIAIQLRRVLNEGGGDNNNNNNSNGSSSSLVAVAAATPFAEKLVKEVLMSFTNSLSFLNDPACESHEVVSDLQQIRDTNSKSEDSLESNCKSSIVSNKERRGCYKRRKTSQTWEKESEFPVEDGHQWRKYGQKMILNAQYPRSYYRCTHKYDQGCRATKQVQRIQEDPPLHKTTYCGHHSCRNLQNPEIIVDSVSPSHDHSSMFLSFDNSLPTPSKQDCPFLSLSSSSVKRECKEEEEIAIPDLPPSSSSLNNDYLIPSELTFDDDSQRHHALLSTTLDDVSLYDSAELDDVLGGLLNEIR |
| GuWRKY12 | MARGGGLSIDSDPTGSFFHHKPIVLNSFPEDNNNIISSNSHHPKWKLGHNMDGTVNKRSSNSSNTPNSTTIPFQVNLSCSHDSHASSPPSDHDHNNNNNRPLIDEMDFFSSHHNKNNDNFASASTSAPHTHDHHSPTPAILELKVNTGLNLLTTNTSSDQSMVEDDISSNSQDKKAKLELVVLKAELERMKVENHRLRNMLDEANTNYNALQMHLASLMQDRKAEEEGDIEEQQQQQSGNIGGGASLVPRQFMDLGLATNASDIDEPSLSSSVGRSTQDRSKSPANNIEVGSKMARKNGNGGDELAPVFDHDHKKDRGIEREDSPQDQVLASANNNVPKFSPPRNIDQAAEATMRKARVSVRARSEAPMITDGCQWRKYGQKMAKGNPCPRAYYRCTMAAGCPVQRCAEDRTILITTYEGNHNHPLPPAAMAMAQTTSSAARMLLSGSMSSADGLMNANFLTRTLLPCSSSMATISASAPFPTVTLDLTQSPNPLQFPKPQTQFQIPFSQNFANSTASSLLPQIFGQALYNQSKFSGLQMSQDVAADPSQLGNQSHQQAQPQQLADTVSAATAAIAADPNFTAALAAAITSIIGAAQPHNNNSSSSNNNGNTTANNSSNGNITSSNNNSNLKQ |
| GuWRKY13 | MEEVEQANRAAVESCHRVLSMLSQPRDQVQHRNLMVETGEAVMRFKKVVSLLXNGLGHARVRKFKKIQQTPFSQTIFLDNPICKTNHHYSKNLQFPQTSYPENSVQELGSTIKNSLSLGNPSLELSSSGKSPLQLAQQASSTHYHFFQQQQQQRLLLQQQQQQMKHQAEMMFRRNNSGINLNFDSTSCTPTMSSTRSFISSLSIDGSVANLDGSSFHLIGAPHSSDQNSQQHKRKCSARGDEGSLKCGSSARCHCSKKRKHRVKRSIKVPAISNKLADIPPDDYSWRKYGQKPIKGSPHPRGYYKCSSMRGCPARKHVERCLEEPTMLIVTYEGEHNHPKLPTQSANA |
| GuWRKY14 | MYHRSRFNTQPLYVADPDEPEPISEPGPASPSSGEDTKTEAPSPQKRREMKKRVVTIPIGDVDGSKSKGETYPPSDSWAWRKYGQKPIKGSPYPRGYYRCSSSKGCPARKQVERSRVDPTKLIVTYAYEHNHSLPLPKSHSSAASASTGDGDGAAASPEESAARFQPEELTVFATQSDLELAGDSAVLLCHHHHAEFGWFDDVASTGVLESPICGEVEDVTMPMREEDESLFADLGELPECSVVFRRRNIPSASAIQCGGITG |
| GuWRKY15 | MENNYSMLFPCPNSSSSSSYPNISTSGVGITSQHGFNGMSNLSGQSSNNAFLGLRPIINNNNDESGDFHHHERREEEGAIRDVNVSAGIKKKGEKKVRKPRYAFQTRSQVDILDDGYRWRKYGQKAVKNNKFPRSYYRCTHQGCNVKKQVQRLTKDEGVVVTTYEGRSAPPTHYLKINVRRRSVFYLTCILCSGACAYEEKRGMSSLLGQVGIGAIWRPKRVKHWGC |
| GuWRKY16 | MEGQRKTIIEELRRGREFANQLRQVLLIINGDDDELAITATPFAKHLSNNVLKSFTNTLFLLDKYPNKKXHDHEVLSSQILQQSWEFPFTSSPAKSEDSGRESCKISAAKEYCRRGIKSLYINVNLESLVCRKTTETLEKVSEIPTDDGHQWRKYGQKRILXTKYSRDYYRCTHKFDQICQATKQVQRIXEEPPLYKTTYYGHHSCKNFLNPEIMLDPSSPHHGTSILLSFNNTFPTPTKQDCPFLSSAVKEDYLLSPEPTSDNSTRHVTLSSTLESNQKXVMYGGLYDSVELYDVFQSNLLGFEDQLSMF |
| GuWRKY17 | MEETSSKRKHVSLINELIQGKELAKQLSDHLVTSYDPSISHETNELLIEKILSTYEKALTMLNCGSNVGDTKTISGNMMMDSHCSSTNESPRSEVMDLEFKHKALFKKRKTMPRWTEQVKICSETGIEGSLDDGYSWRKYGQKGIXGAKFPRGYYRCTHRNAQGCLATKQVQRSDEDPTIIEVTYRGRHTCTQAKHLNKESPSKTKMGLEENQHHNDQKNQLVQQEKMEQTPEAIFKFKAELEVKTXDLETKEDIFPWFCFPSPSIGSENDFMESFSPAFISPATSESNLFCLSAYPLGSTGXCQNPQTSESDITDTVSAPTSVTNSPLXDLDILLNKGDFDTDFPFNTPEYFSS |
| GuWRKY18 | MAIDAIGVPQIKMEEQMAISTIQEAASAGLKSMEHLLLLLSSSSSYNSSSSTHNNNNLDCSEITDFTVSKFKQVINLLNRTGHARFRRAPPPQNPKPQQQPQIHEFTLDFVKPNNKLDETDLSISTTNTNSSFLSSITADASVSDGKIGPFLTPSATAKLPLSSSHRKRCHDAAPSSSSPCHCSKKRKTRVKRTIRVPAISSKIADIPPDEYTWRKYGQKPIKGSPYPRGYYKCSTVRGCPARKHVERARDDPNMMIVTYEGDHRHSQPLITASAVGFALQSVESSNGGYGAVWKAHGGKR |
| GuWRKY19 | MEEVMNSIKRACELARNLEAELPNMVNHPDVLFISIDGVVKAFGDAKQKVMMVLSQQDMTTTTTTTLTTPSSFAPMLLPHHDDDASIXQDTTTQQTQTGATSTTQMQDLMNQLLLMQQPXDVRTLLENKMISTGGDLNQMLRSTRGTLRIGEXVGRDIVEGNSERSKGRKNDLEKRTVMFPAPQVGNTEMPPEDGFTWRKYGQKEILGRKHPKSYYRCTHQKLYACPAKKQVQRLDDNPYIFEVTYRGEHTCHMSSTAPSSYPPPQVLLGXMSTQTTNISPQLSPSSTSVSGWLSSVNLCLGAGGGGAGAEPSTSRYGPDNYPVADMADVMFNSGSSSGNSMESLFAPTEDKWEPGEKKS |
| GuWRKY20 | MDIHHHCPESVSKKRVIIKELVEGQEAATQLKFLLENPFGEADAAATDSSSLSSEELVASVLRSFTQALSIITSYQNNNNLLNSSGEXGLPVTGDSTSDKRKXSFPPAARKGATAGRGCYKRRVQRHGPQFPTTPHDNHAWRKSYFRCTHKYDEGCSARKQVQQTKENPDMYQTMYIGIHTCKATPKAKATTTHDHDLVTNSSSSTCWESYLETEVLPPSEVQDHNISSSTSLAIKQEYPKEEHTPNDHKPLKMESDIDAAVYNSSSTVGSDQCLLMDFGVESVHFCTDLFHFDENPEREKMDIHHCPESGSKKRVIVKELVEGQEAATQLKFLLENPFGGADADSSSLSLAELVANVLRSFTEALSIITSYQSNNNLLNSSGENGSPVTGDSTSDKRKRSMPPARKGGRGCYKRRKSAETWTTVSHTTHDNHAWRKYGQKEIXNSEFPRSYFRCTHKYDEGCSARKQVQQTKENPDMYQTMYIGIHTDRRS |
| GuWRKY21 | MTERNPARQRGAEAEYSSPDSDFSNLQWPLELSEYLKFDDDNNQWLHDDINPTTESFVSNQQVYNIQANQVGGGDFVEGGGSSHFEGGSREKEARERVAFKTKSEIEILDDGYKWRKYGKKMVKDSPNPREEAVRVEDGGGGGGGGERGERGCDIDVVGAK |
| GuWRKY22 | MVRESGLSMDSESDPIGSSSSSLHNPIVLNSFTQDINNHPKWKLSPNYNNNNNNNMEVTVSTTTKGSSSSPTRTTTMPFQVNLSCSTDNNHTSHDTRTEMDFFKDKNINDVDDHKVVASASVPDNNDHSPTPPMLEFRVNTGLNLLTTNTSSDKSMVDDEISPNSEEKRTKNELAILKSELERMKTENHQLREMLEQVNSNYNALQMHLASLMQDQKAEESEEQQVFDGKKRSENGGAVLVPRQFMDLGLATNAETDEPSSSSMVRSQDPSGSPGGNNNNMEVASKELVKNGNVVSDEGLVYDEEKKEFGRGGINNEREDSPSGHALAADKVPRFSPPKNVDQAEATMRKARVSVRARSEAPMITDGCQWRKYGQKMAKGNPCPRAYYXCTMAAGCPVRKQVQRCAEDRTVLITTYEGNHNHPLPPAAMAMAQTTSSAARMLLSGSMSSTDGLMNANFLTRTLLPCSSSMATISASAPFPTVTLDLTQSPNPLQFPRPPNQLQIPFPAGIPQNFANSLMPQIFGQALYNQSKFSGLQMSQDAVTDPSQLGNHSQQAQPNIADTVSAAIAADPNFTAALAAAITSIIGGAQPNNNSATNNNNGNMTANNSNGNGNGNVTSSNNNSNGKQ |
| GuWRKY23 | MDKGWGLTLDTSSSSSSPISLPFYPNSKVQPPAAAAASSSSVNPAFSRLSTHDRSNMFPILGFPVNLVSRNTNGAAXDGDGHHHDSRKVVGEVDFFSDRNKSSPSPSPSAARDEHQHVKPNNTITTTTTVKKEIIHTDEKPPKSNIHVNTGLQLANTGSDQSVVDDGVSSDAEDKRAKTTELAQLQVELQRMNAENKKLKEMLSHVSGNYTALQMHLVALMQKNQHTENTENEVVQAKAEEKNQGVGTGGMVPRQFLEIGPSGTTAEVDDQVSNSSSDERTRSNTPQVEAGTIRDHTRNNXNGKNGREDSPESESQGWGPNNKVQKLNNPSNSNNNNAMDQSTTDATMRKARVSVRARSEASMISDGCQWRKYGQKMAKGNPCPRAYYRCTMAVGCPVRKQVQRCAEDRTILVTTYEGTHNHPLPPAAMAMASTTAAAASMLLSGSMSSADHGNIMNPNLLARAILPCSNSNMATLSASAPFPTVTLXLTHNPNPLQFQTRPXAPMFQVPFHLPVQQQQPQSFGSGPTPFAQPQPQAATLYNQSKFSGLQLSQEMGASSSSSSSQLASATQAPTATQQQHSLADSVSAATAAITADPNFTAVLAAAISSIIGGGAHANNNNGHNNSSNNNNRTTISNFSGNL |
| GuWRKY24 | MEKSKQMNMEEYAAIGSSSSFPSCYSFPSVFDFSEDNKSSLGFMELLGVQQNYVPETGKQECSEVLTSQQQPATPNSSSISSASSEAVNDEQNKTVDQANQPHKLWAKKTNQKRQREPRFAFVTKSEVDHLEDGYRWRKYGQKAVKNSPFPSASCNVKKRVERSYTDPTVVVTTYEGQHTHPSPVMGRSGHAAGSPIFSSECTNYNFGSVVAAGNYNMSQYYQHRQQVLVNTLSSLGFPSSSSSSSSSSSSKNINNATFASQERPLCNPGATVLLRDHGLLQDVVPSHMLKEE |
| GuWRKY25 | MDSNASPQLESEEASSELKSETQASKKRKMVQKTVVAVRIGENVSKXKNEGLPSDFWSWRKYGQKPIKGSPYPRGYYRCSTSKGCSAKKQVERCRTDASMLIITYTSTHNHPGPTSLSTTNLPQQPKESETEETTEDPPVTSKEEXQQEQIEEEEMNKKHNHNVSSDEGINEENFHYLQSPICCSENIIIIDQEDPFKLNTEKSHDRIDNLVLEEEPLCYAQLKNLSDSKSEELDFFDELEELPMPSSFLQFTRSIFSDERIPVAPS |
| GuWRKY26 | MELVSERSRHEAGGKGAKEGKRSESSGDDENLPEQENIVAQVDELEKTKVEMGGVMEENERLKMRLNRILNEYRTLQMQFNNIVEQETKNSSDKLNNIIEESDELVSLSLGRLPSNPIAKVSNKQTLKEEEEHEKEGLALGLDCKFETSKSGSTTEHLPATNNPSPTNSSELVPKEEAGETWPPSKTTNKAAIRSVAAEDEVISQQNPAKKARVCVRARCDTPTLNDGCQWRKYGQKISKGNPCPRAYYRCTVATSCPVRKQVQRCAQDMSILITTYEGTHNHPLPLSATAMASTTSAAASMLLSGSSTSSSHPGLTTIPSTYTTTTASAGLQYGMNFSLSDASINKSKQFYHLSHPSLSSSANSHPTITLDLTSNPPSSSSSPFARFTSDYNNPSRYPSSTTSLNFSSSESNNAVSWSNGMNGFLSYSAAPQQPYNNRNNILSTININPVRQQPMENNNIYSSYMQRNNSNIPIQSQQQSLPDSTIAAATKAITADPSFQSALAAALTSIIGTKSTTATTTQGNHHQQGSTSVGENLGQKMKWGELFSSSSALPAASTASKVSSGCASSFLNKAPSAATTQTGSLMCLQQPLPFPGPKSASASPGDNRDTTN |
| GuWRKY27 | MENRCSNGRSGAMEQELIKGLDIANQLLEVLAHHHDQSNTQDNVVERLRSPFAEDLIRKILRSFTNTLSLLSTNNDGDVSDKEEVVPLTITNFSSSTILPKNEDTNDESCKSYSNAKKRRGSTAPTWEKDSSILIEDGYAWRKYGHKMIMNSKYPRSYYRCTHKCEQGCPAIKKVQRIQEDPPLYRTTYCGNHICKASLITDPETNLEPASSLGSAMFLSFNNNNFQSKEEYPFSSTLFASAKQEPMEVTPDNYDHINAGSIQLSSSDYLMLCDYELHCERHATIKAMEEELVKGRDTANQLLEVLVDHKLNTHHGDVEGLKSPFAENLVREPIMMSPMRRFCLSNIKDFSSENSPKPEDHMDKACKRFFNPKNRRGCYKRKLNALITWEKDSSILIEDGYGWRXYGQKTIMNSKYLRSYYRCSYKDDQXCPAIKQVQRIQEDPPLYRTTYYGHHNCKTFINPEMALEDENTSSGSSKFLSFNNSLPSKEQYSFSSSLFASTKQEPVDEVIPYYHIPHNQSITSSDYPPSCDYEHDFNYLRHATMLSSTESKNTSILVEDGHAWRKYGQKKIMNAKYLRSYYRCTHMYDQHCQFFFFFQRIREDPPLYRTMYYGHHTCKSSFNSDINLEPFLSSDSSILLSFSNNVIPSKQEYFFFFSLASTKGEPMEEIHEDFFFFYQLSSQENLLLCDFEVYFDYSGHGATTLSSTKSVEFENVYEQFGF |
| GuWRKY28 | MESTCVDTSLNLNIVPSLHMDEVLVEELHRLSCENKRLSETLNHMCENYQAMQNQLSQLMNKNFEHPTTQQSRKRKADQGEIGCINKFGIRGNVVNTVDQCSTITEEESLIKRPMDIISPKVYKVLVKTEATNNSLVQKSVEDPTILVATYEGEHNHGHQKGEMSLVSRQSDQESPVMCVPPISSPTPIIQSTSPTVTLDLVEENINVQKSSIQQFLVQQMATSLTRDPNFTAALATAISGRILDQTSNKAKCLLERLQACCIVETLQKELQHVRKENNTLRLMLEVLSSKCTKLESHLQEINNKAEQKGILSNQVGSVPSLDASKRARLEFPTAKKPLQIFFRTHPNNDSLIIKDGYQWRKYGQKVTKDNDSPRAYFRCSMAPSCPAKKKVQKCIHDRSIIVATYDGEHNHSVPHESCRPSSSTPKGSSIIANELPPTPNEKEAINTDSDIPGWAQTSTELCENVMQQCGYGRXINIEEYVGSLIKDPDFTTALAEAVARTITDQHKQQQDLNLNLDLPER |
| GuWRKY29 | MAVELMMGGYSRNESSSFTATAEENAVQEAASGLESVEKLIRLLSKARQHYNNNNHHSSSPSSSAASPSNPNPNPNPPPSSMEIERDCRAVADVAVSKFKRVISLLDRTRTGHARFRRAPLPQPPQQNQTPQQTQQPYSEPIVFKATPLQQIPPTLHHNHHHNHTERKDSSKTLNFSYSSAANSFISSLTGDADAKQPSSPPLPLPAGAFQIXNLPQASSAGKPPLSSSSLKRKCSSETLGSGKCGSSSSRCHCSKKRKLRLKRVVRVPAISLKMADIPPDDYSWRKYGQKPIKGSPHPRGYYKCSSVRGCPARKHVERALDDPSMLVVTYEGEHNHSLSAADASNLILESS |
| GuWRKY30 | MVLSQPMSKTMIMEEVMMGGANWSDIGSEDDDLLVRELLDDDGSPPLLMLQPEELTTDINQPNNNNYTREGSSRDQLASFNRLITNIYSGPTITDIENALSVTTNNQRDHFQQFSSARVSILERSLSKIENKYTLKIKCFGNGMGDDGYKWRKYGQKSIKNSPNPRSY |
| GuWRKY31 | MEEAMACNDIAMDNSSSGGVIAHSSSVXSFNGKRLVMDEMDFFAENDNKTISSPVNDHHQTVVHQMELHTSLDLLTKISPSNRSTLDVEDGASEAKSNKKNEFVALVAELHHMTAENQRLRELIDRANNDYNALHKHLMKLMQEQQHKQEITSGGTGEKSDGTMIPRPFLDIGFGEKDIETSQQSSEGKVVVGESKGSSMVHELECSNKIRKICSSGDVVHELDPQDHERPAAPNNNKAFXGWLSNQVPIRDNNVDDQASESMIKKARVSVRARSESSMISDGCQWRKYGQKMAKGNPCPRAYYRCTMGTGCPVRKQVQRCAEDRSVLVTTYEGQHNHALPPTAKAMASTTSAAASMLLSGSMPSSDGLMNPTILESAXLPHPVCSQNMATLSASAPFPTITLDLTQQNCAPNNNTNSSHQQQGQQLSQLLPPLLAHKFMSAAPXGFTADTVNNATSFADTVNAATAAITADPKFTAALVAAITSIMGSSRSPNNNGTNGSTTSGDQHCNNA |
| GuWRKY32 | MIYKQMNILQVEMDRVKEENKVMMKMMEQTLKDYYDLQMKFAATIQENNKKIDHHISLSLQDNTNSSSEGPPMIPEIFDNNTQASPSPRKTDDHILTESELSLSLRLQTTTTSHDDEKLDNKEELSSFAISEQNKKLRRTHHDLSGMSSTMHNAATSPPNRKTRISVRAXCEQAKMNDGCQWRKYGQKISKGNPCPRAYYRCTVAPGCPVRKQVQRCQDDRSILITTYEGTHNHPLPVGSTAMASSAANSSVLLDSGNPIAKYGTSSFTQPSINPYNTFHPLNPSFNIVRSNISPSCDPSKGINVLDDLTNNNNNNNNLNGSSLRFHAASSSNVTAAKQRFPWPVQNKHQSSGNAIAMNNFHXLMNERVRKAEEPAKPLADNINASAIANDPKFRYAVAAAITSLFNKEGHVN |
| GuWRKY33 | MGMRTARERKPEVGKTGKVSSGERGEDSRKKTMLTLAPHQQDKARDRLLHVPRTVSATVKCRCQSQTRVTLPSIIPKPKIGTEGSMYCDQKQLFSSLSAAYDSSQLAFDPSSYMSFTECLQGGGGGMDYNSLIATSFGLSPSSSEVFSSVEGNHHHHHHQKTPAEAEAEAEAEAGEEDHNMGDGGTTGGGGAAGSETLLNSSISSSSTEAGAEEDSGKRKKDGKVKEEQSSKNGSIKEKKKGEKKQKEPRFAFMTKSEVDHLEDGYRWRKYGQKAVKNSPYPRSYYRCTTQKCTVKKRVERSFEDPTTVITTYEGQHNHPVPTSLRGSNAAAGIMFTPAPLLSTPTPLSLSPSAGSNFPHHLLLHMHQHTNTNYNPHHHHHHHLTNTNTNTNTSYTPAPSASAASSSSFYSHNNNIHNSLLHHQLPPDQYGLLQDIVPSMFLNNTHHN |
| GuWRKY34 | MDTSVTSSAKRRGIKRRVVEIPMKEVESSNTPPSDSWAWRKYGQKPIKGSPYPRGYYRCSSSKSCPARKQVERSHVDPTVLLVTYSSDHNHPSPPPRNHRNSSGNAIISNSIAASKPDKESEPEPEERFADLGDGSLIAAADELGWLGEVEAMASPAVLGSPIFSEHEATVLGEEEESLFADLGELPECSVVFRRGVLXEREEEQRRRFACGTTG |
| GuWRKY35 | MRRNMVVDQVVFPKMRVEEQVAIQEAASAGLKSMEHLIRVLASQASSSCSSSSANQRNRLDLNNLDCTEITDFTVSKFKQVINLLNRTGHARFRRAPSHRPSSPSPVSSQPQPQPQTQSMTLDFAKPLPLKSNPNPNPNPNPSTDLSVSQYSMSNKVDTYSISTTTSSFMSSITGDGSVSDGKIGPSILAAGKPPLASSHRKRCHDATLSGKVSSSGHCHCSKRRKSRVKRIIRVAAISSKIADIPADEYSWRKYGQKPIKGSPYPRGYYKCSSVRGCPARKHVERAQDDPNMLIVTYEGEHRHPQTTGATAAAAAFTAQPV |
| GuWRKY36 | MDYYFGNPNPPNPYYGHQYSAVNIAPSSDNFMVSDYLMLDDDDVVNVDHHHHQDSNWSQSTETXSSENKAGSSDANIHGFDFNLLLIYIYISIIITYTILDSFDRKRKNGIXKSTAEVSPRRITFRTRSQLEIMDDGYKWRKYGKKSVKNSPNPRNYYKCSGEGCSVKKRVERXREDSSYVLTTYDGVHNHES |
| GuWRKY37 | MGNAREENERLKLSLSRVVKDHQTNFSSHEENEESELVSLSLGISSKGQPIMDEKKNNTNRDGEKIKEDGNLGEGLALGLKIKFDPLGNGDQGKEEELTERWPPSKVLKTTRTGDISEVSQHAQPKKARVCIRARCDTQTMNDGCHWRKYGQKMAKGNPCPRAYYRCTVSPSCPVRKQVQRCAKDMSILITTYEGTHDHPLPTSATAIAYTTSAAASMLQSPSLSSQQGLPNSNTTFPIVNSNVAYNLYALNYTSPTMINHQLTSRPQQLYLNNSSNXSSSSNYHPTVTLDLTTPQTNSSFHIGKFPPTPFXSTPKHSSTTNFNVSPTITPLQSSMLKSTWSPYSGCLNYGGLITAQNRNQYGSLMNNEKQPIFQGHLYQPNYIMSNHAISQQPFTNSIATTTKTITTNPKFQSALVSALTCVDGSATTCGGVKENHVVVESAPLSLKLGEDIPYTKDTV |
| GuWRKY38 | MEEVINSIRRVCELAKNLETELPNLANQPAMLSLSIDEIVGTLSCTKERLLSLSSFSDEMLQQLHETQQPQMDTTLLMQEWLRSSCAMTIVDDDQLFKMQQQLQASRSTTRPFEARSKGSQGEVQAIEASHSRSRRRRNIDLEKRKIIVPAPLFGNTEMPPEDGFTWRKYGXKEIIGSKYPRCTHQKLYECQARKQVQRQDQNPSMFEVTYRGNHICCHVSSTAPSLVPPPQLVVDVADVMFNSGGSSSANSMESLFPPAS |
| GuWRKY39 | MSVINFHEKESVIEWGNKRKRVIRELVKGREYATQLNHFLLHRQNPTAAAVGSPSAKELVTNVLRSFADTLSVLTSSEAGTGSNDNPRSKEDSRESRKRLLPAPTKDRRGSYKRRKTEQTWTKVSHTTTDDNHAWRKYGQKEILNSQFPRSYFRCTRKYEQGCRATRQVQRIQENPAKYQITYIGFHTCKPTTTTLEEAPQAQMVTYNSDTTSCWDSFDLVNSQPDESKITSPTESQDPPQSSLSIKQEYPNDDRTTSPSDLITDNLLDPNLWSDYLKDFELSKPPAINIRFKMASHDNIADTVYSSCCTDHDDHSQNLHEMDHFGVFSSDPHFTTDFIHF |
| GuWRKY40 | MEEKRXKKYSFINMANSVAFSDEIPNMSMSSSFNFPLTSSSNSSSIFDMMLPPPPPPPPSCGDDHDQKASSFGGYMDLLGAQDYFPPSLFDWFPSGTIPNTNTVDAAATSAAPAAAQINNHPLPSPAGSEVVNTPATPVSSSISSSSNEATVGNKHQLAEDDEEGDADAGREDNQNQDQTKKQVVKAKKKNLKKQKEPRYAFLTKSEVDHLDDGYRWRKYGQKAVKNSPHPRSYYRCTTAGCGVKKRVERSSEDTTIVVTTYEGQHTHPSPALSRAASLGFMQHHDNASSAQYFGPTSGGGAQYGSGSALGGFSNSRHHFVLSLQQFQHQQATAFCTTSPLSSGNYANASSFGGGFLRNQEHNLHGFVPSSRLMTNNNDIRANLLMDNNHNGLLQDIVVQPTHKEGSMK |
| GuWRKY41 | MFNGGNGDRPPWKEEAAAGGGGPPVRPTITLPPRTDTLFNGGLSPGPMTLLSSFFSDGGDDCKSFSQLLASSMMSPAAPPAGVLDSPGFFSPQVPAATSTYTTQPQMPPSMEGSQAAMTESTNYSLPEQRLQQSSSLNVDKPADDGYNWRKYGQKQVKGSEFPRSYYKCTCPNCPVKKKVERSLEGIADSSYQGITTNSMSKMDPESSQATAEHLSGTSDSEEVGDHETEVEEKNPASSHRTVTEPRIVVQTTSEVDLLDDGYRWRKYGQKELLQMYNPGASTDPKATNSHTIANNSASQLRSQNTIPPERHGFGSSSGVGGNEQQPVARLRLKEEQIT |
| GuWRKY42 | MSHEDWDLFAIVRSCKAATFTTPTTTTQTPPTNTRSPHQNTTKLEPLQPNIVQPKTNGFLELHQLLVNNFNPTTTTTTTTTIATTSTGGNCGINPNSTTFSDLTGVQHKMQQQGLTNFTSFGASTTSFDRFHHQQQQKPQPTEQHQQQQQEEAINQLPLQVIPQTNYMVLQNTQPQTPRSRKRKSQQKKMVCHVTADNLSADLWAWRKYGQKPIKARKQVERSTTDPNMFIVTYTGDHKHAKPVHRNSLAGSIRNKPXTTRLPAAPETGSPQNAGSSSSSVLRSGPPENGEPECPDPDLKPDDEDDDVLIPNMAVMSDASFLLGLNHFDSDAKPSDGSDPNSVPGFDPNILSGPGLQEV |
| GuWRKY43 | MSTLHGRDMNIPLSRLVIYTCTASHSSPIMSFRGKRPVVVDELEYFFAEKKKNSEEMMMIKYWTAEYTSLDLLKKNTEEDAASETMDDNRNEFVALVAELHELNAENQRLRELVDQVNDDYNALRMQLIDLMQKKQHNHGSNYRAMGEKGXREDVDPRPFLEMDNDEPSQQSSESTLRESTSMVGLMQCNKAQHXWTRKDKMDCGMESSKTNQALFGRPPSNRVPRLSSFRDSESDTMSMIKKARVSIRARSQASTIADGCQWRKYGQKIAKGNPCPRAYYRCTMSTSCPVRKHHNHPLPPTAQAMASTTSAAASMLLSGSXSSSSDGLIDHSLLESVALPSSQNMATLSASAPFPTITLDLTQSATKSSQLYNSTFGSD |
| GuWRKY44 | MEEDWDLHAVVRGCSAVTSTTAVPSVSSSSSGFGTSCSLLQPEASSSCGFSIFDGEQQKGQIFSPSAAYPFEARGSIQELHELCKPFFPKSHPLPLQTSPLSSSFSYSSVAAPPKSAQTQEKQQQQQRIKQPHQHQAGSVTTPRSKRRKNQLKKVCQVPVESLSSDIWAWRKYGQKPIKGSPYPRGYYRCSSSKGCLARKQVERNRSDPTMFIVTYTAEHNHPAPTHRNSLAGSTRQKPLTPQNATTTTGSDSDKALTKPSSPANSGAEEDQEVPTQGEKSDQSREEEKEDDEELLGEDEFGGLSDMVLSDDFFESFDELSQLSAVVTGGECFVDPFSAIAIPAWSAATAASGS |
| GuWRKY45 | MGDPMESQDPPPPPILMMTPXNNNNPFLFTPPTSMLQNPLDPHQGLLVADDNIDWGNLFCGQNNSNNNLLLVGDNIDDGNKGIAMDQCASSSSSLMMVNNEINGTSTDTHIHHRDQYQGEEEEEKGNNNKKKEVERRVVKSGRVKKATRVPRFAFQTRSVDDILDDGYRWRKYGQKAVKNSTYPSFSNTIYVK |
| GuWRKY46 | MKSKFSXPYINELQEDTDVSQENIAESPPNSASTAFNIDGMVTSPSTSSSSAKRSRRAIQKRVVQIPIKEIEGPRLKGESNTPPSDSWAWRKYGQKPIKGSPYPRGYYRCSSSKGCPARKQVERSRVDPTMLVVTYSSDHNHPWPLSRNHHGSRHTTTAKKPEPDPVEPDEKFSELCGDVELGWFGEMETASSAILESPIMAAEFDADVASVLLPMGEEEESLFADLGELPECSAVFRHGLLDDRRRFTPPWCGTTT |
| GuWRKY47 | MENHQHHQMQQHRRELTFLSSDNFLRGQNPDQMTGEHSNPSIKEVDFLSSSPSPPNYHTDNNYNNQEDDQHSPRKIAKESTDGLVNVSFQIKTQNIVLNLLPMINTALNLTCASAAGMSRAANDENPKAVLRLSTLRRESLRLQEENSRLRTMLDQITKNYSQLQLFIAFQKQRLYQQEVEVGTNGMNSGQHNFRDPRLCTKLDVNIIKPVSDGDHQEVLVSPSNQLDHHQHHPLTKLNNDGPGKQACPVLTHEDVLDHQSSPSKLEESIAGGHGELPFRKTRVSVRARSEAPMISDGCQWRKYGQKMAKGNPCPRAYYRCTMGVGCPVRKQVQRCTEDRAVLITTYEGNHNHRLPPQATAMANSTSAAAAMLLSGSARSNEXALTNSAGYFSSVSLSASAPFPTITLDMAHYPMQLHRDPFHXAPASNFPQLVFPQNKLLQPSSSMVETVTAAITSDPNLTAALAAAISSFVGVQPRSPTDDXIGNGTSVLPGVHAFMH |
| GuWRKY48 | MGRGGEEETTTTAKMEIDLSLKIDSDDEQQEEKDQEEAEIRAAKERVNSDKEEVPAEATSGEIEDDASMVEISLPDNTKTKELSALQMEMESMKEENKVLREVVEQTMKDYYDLQMKFSVIQENNKRKDHQLSLSLQDHNAITTCSGGEGPSRILEILNKKIQRAASPPPDHSTTDDDSISESELGLSLRLRTNTCQKETEGNNNNNYKEAEENKKEHELASPLSVQSNKSQRIHDNNNMPGITTHAASPPNRKARVSMNDGCQWRKYGQKIAKGNPCPRAYYRCTVAPGCPVRKQVQRCIDDMSILITTYEGTHNHPLPVGATAMASTASAAASFMLLDSSNIINNNTISADGTSNSFTTQPPSLIPNYNYHNNNSLFHPLNHPPQYSSNMIRSINPNDPSQGVVLDLTNNNQRFSAAGSSSSTEPPRFSWNNMQPNKYQTSGATAITVNNNNFHNNSXRVPILDHHDENVSAIASDPKFRVAVAAAITSLMNKESHTPTTTTTTTHPIGGTSSFGPYRMSTTGKNGSSSSTTTNNWGH |
| GuWRKY49 | MAVELMGFPKMDEQKAIQEAASEGLKGMEHLIRLLSHQPSHLQTDLTDVTVSKFKKLISLLNRTGHARFRRAPLPSPPXQPPANPSLLHHPPQPQSLTLDFTKPNALTTSNAKSLDLEFSKETFSVSSNSSFISSAITGDGSVSNGKQGSSIFVAPPLSAGKPPLSSAPFKKRCHDHSDNVSGSSKCHCTKKRKNRVKKTIRVPAISAKIADIPPDEYSWRKYGQKPIKGSPYPRGYYKCSTVRGCPARKHVERAPDDPAMLIVTYEGEHRHAIQAAMQENISGTMGLVFEST |
| GuWRKY50 | MMDIEEAERVVVAKPVASRPTCSTFKSFSELLAGAINASPPIASSHQTTVSAIRPKTVRFKPAAAMNRPPAGSVSSQADIFGAALSNSSDMSPKPDTKQSLIYKPMAKLVSKTTVSLLANMQNLEEDQKALTSSNTTNGDRPYDDGYNWRKYGQKQVKGSEFPRSYYKCTHPNCPVKKKVERSFDGQIAEIVYKGEHNHSKPQLPKRNTGSGTQQGSDGMVQDIWSNNNQSERNEGRIENNQNDTTGLSVRSAYHVKAPQPNDSALIGGAINAGGGSLENSCGLSGECEEGSKGFEAEEDEPRCKRRKNENQSNEAAASEEGLVEPRVVMQSSMDSEILGDGFRWRKYGQKVVKGNPYPRSYYRCTNVKCNVRKHVERAMDDPRSFVTTYEGKHNHEMPLIKNTGSSVASEKDSQASLSKDKPC |
| GuWRKY51 | MENEWSWEHSTLINELIQGMEVARRLKEDLRTPYPADTRDLQVQMILSSYEKALQILKWNESTSKLQTMNRAVTLLPESPPLSDDVDGGIQDHQEIKHDSKKRKIVPKWTDQVRVGFESGLEGSHDDGYNWRKYGQKDILGAKYPRSYYRCTFRNTQNCWATKQVQRSDEDPTIFDITYKGKHTCSQGSNAVLPLKSPGKQEKPDRHNNDIRHAQPSQESFTKFRNTLTVRADNLGNEEMACPFTFPSTSFGCTTTQENNSLIPSAFENDSFLSSLFQTQYVSPTTPESNYFSSPTFHMNGFDGIYNKPRSESDITEIISTNTSATNSPIPEFNFSLDPVEIDPNFPFNTPGFFS |
| GuWRKY52 | MEEDNNSNNNNESNNGGDEIFVPGFPEKRSIAERRGFNSNAARINTALFRASTTPLSSPAARSPRLTIPPGISPTALLDSPIMLPNSQALPSPTTGTFFMLPPLIDEGGSMLTSVTLEQRNVDALPTASDAAASFKFKPQSNLDPNPLPASLNQVPSNCHMVNGGNRDGQILVQGQPPLDFSFPGDFPKGHSARSSEIHLYHDVKMVNDAIVNANNNVEMPISRSEEASDESTLPENAIHEIAEAGETYDKVDAXSVWGNIQSGVKDTKHSLEWKADGQERTSSASVVTELSDPISMKRGRSLCMFESEDTPELSSTLASHDGDEDGATQAVVSVEDDAEDVESESKRRKKENYTVESILPPRNVREPRVVVQIESDVDILDDGYRWRKYGQKVVKGNPNPSASIPKPETHQTLAPHFDRKPEFSNDFLRSSLLGGFSNDMKFGPSSISQMKYSSLNNTIPYGSYGLNPDRCTAPQAGSIASMFPDFPMPLPLNLPSSGSFSLAGINFNSVKPMASVQSFLSGQQVKEIDTGFLRPKQEQKDDTIYGTCIPSLEHSSSSLTPSSASPSIYQRVMQNFPS |
| GuWRKY53 | MAEVEEHNNKRTFEAEQNEEQRNENAEVEAEAPHRVTDSAQLNSETLVVSSSLPCHHTAHLQGSSTAHKGGKDESKEPAGPPDKETIVEVAVEGPQMQTKNQFQVSVCSPPLSELSPTSVTQSLSSAPSPTVPEHRLSPPKAISEHVLEVDKRTPGGKTLPSVSVARASDGYNWRKYGQKQVKSPTGSRSYYRCTHSDCSAKKIXCCDHSGHVMEIVYKSQHSHDPPHKTNSTRESKSLPSNEPNVENTVPNQSTRVLNDSDPSPSPKNPLQEEPCSADKKRQSSPNGENGKVNLKEEHDEPEXKRRMKKGDLTSLDSPSKKTDSVHNQKTSTQWSVDTEGELTGDALDLGGEKAIESARTLLSIGFEIKPC |
| GuWRKY54 | MGNAREENERLKLSLSRVVKDHQTNFSSQLKKMKKMNDGCHWRKYGQKMAKGNPCPRAYYRCTVSPSCPVRKQVMEKEKNGMTMHYGITKCYSTH |
| GuWRKY55 | MVSSEESADHNVSSDKVQQRVSPQRDITLSQGNHDTEIHLSNPEGARSIPSIVAKNEVKDSDATACALQSDQEGRAHSLPREKHLQIPDSLLHELPPSQSGQDSPSIIREKVSKDGYNWRKYGQKHVKGNEFIRSYYKCTHPNCQAKKQLQQSHNGQITDSICIGRHNHPRPQSNTIVPDDRVLPVVEKEPHKPSLANVEAAHVQLTAKYEVHNLEDPESKRLKKDSSNVDVTGVDMSTCESRVVDFVNDGYRWRKYGQKMVKGNANPRSYYRCSNPGCPVKKHVERASHDSKIVITTYEGQHDHEIPSGRSVTHNAATNTHTMDIDGKLGTKSGGNTVCVDTGERNCLDSKSRLNKRPNEESITNSEAGDMAEFRVISLHDSVSNDTVCHSSSKVPCRSNEHAKDEVKTTSEGSKDCLNVVAGHDTPSTISEFSKQSTSDAEPVQS |
| GuWRKY56 | MEEVEEANRAAVESCHRVISMLCQPQDQVLQFRNLMVETGEAVVRFKKVVSLLSSNGLGHARVRKLNNKLKTPFSQSILLDNPNCRTNHHQSTNMQFPQTSFXDNSIQELXSSGIRNSLFLGNPSLELSSNGKSPLHLTQEPSSKTHYNILQQQQQIQQQQRLLLQQQQQHQQMKHQAGMMFRRNNNNSVINLNFDSSSCTPSISSTRSFISSLSIDGSVANLDGGSGFRLVGGAAAHSSDQNSQQNKRKCSARXDEGSVKCGSSSARCHCSKKRKHRVKRSIKVPAISNKLADIPPDDYSWRKYGQKPIKGSPHPRGYYKCSSMRGCPARKHVERCLEEPTMLIVTYEGDHNHPKLPTQSANA |
| GuWRKY57 | MASSSGTERTGSGVPKFKSTPPPSLPLSPPPIFSPSSYFAIPPASQISLSIPNNNKSFSPEIASVQNNNNNNGFQSDYSNNYQHPQQQAQTLSRRSDDGYNWRKYGQKQVKGSENPRSYYKCTYPNCPTKKKVERSLEGQITEIVYKGTHNHPKPQSTRRNSSSSSSLPIPPSNPIADISDQSYATHGSGQMDSVATPENSSISIGDDDFEHSSQKCRSGGDEFDEDEPDAKRWKIEGENEGISAPGSRTVREPRVVVQTTSDIDILDDGYRWRKYGQKVVKGNPNPRKHVERASHDLRAVITTYEGKHNHDVPAARGTTTTTAIRPSAVTHHNNHTTNTNSLQGSVRPQQAQPEGQSPFTLEMLQNPVGSFGFSGFGNNPMGAKEEPRDDMFLESLLC |
| GuWRKY58 | MEPTCVDTSLNLNVVSSRDTHAADEVMVEELQRLSRENKRLTERLNQMCENCVALQKQLSQFSPTNNNFDSELATTPISRKRKAEPESTAGTDDNCVNLTTTTTTTTTTEEETIFKRPKHNTSPKISKVLVRIDASDTSLWRKYGQKVTRDNPSPRAYFKCSFAPTCPVKKKVQRSVEDRSVLVATYEGEHNHAHHQAEIISLSNSSSGQISETAATGSVVPAISYSSPCSSSRTGPTSTALDLVQPSQKSSSIQQQFLLRQMATSLTRDPNFTAALAAAISGRFQF |
| GuWRKY59 | MDCYFVNPHPNYPNYAHPTAHQMTPYSSSEFILSDYLMLDDNCVDHHQESDQSQSTESSSVTFNDASHGFDGAISNNNNMLVKCKNGNKRNKTELERPRVTFRTRSELEIMDDGYKWRKNYYKCSSEGCSVKKRVERDRDDSSYVITTYDGVHNHESPFTSHYSQISFVQPDAA |
| GuWRKY60 | MKINLMLSIVAAIICYSLMVLRSKLDSQLETGTLRMEDHVAVEIDAAEEGERGADDDNRSDSQSGYYSHRHQQNQFLRRRVYNDIDGYKWKKYEEKQVRRSGNQRIYYKCTKSNCPVRKKVERTMDGKVIETLYKGTHNHCKPMVTMERSSSSEYLYSLLPPSETAPIDDMPAQLQSFASHDGSEQLDYDAAPESSSVSGGPDAEAPSSNQVGASRWMCGCNGPTAELYDLGCRKFAVTGLPSIGCIPLQITAKFASLKDRKCAEDENLDAKLYNRKLARRLLQTQAMLPGSRVVYTEIYDPLIGLINQPEKYEDENLDAKLYNRKLARRLLQTQAMLPGSRVVYTEIYDPLIGLINQPEKYVSSNLTSGLG |

**Table S3 Protein-protein interaction data file through experimentally determined interaction as shown in Figure 6. Analysis conducted by STRING**

| #node1 | node2 | node1_external_id | node2_external_id | homology | Co-expression | Combined-score |
| --- | --- | --- | --- | --- | --- | --- |
| WRKY33 | WRKY40 | 3702.AT2G38470.1 | 3702.AT1G80840.1 | 0.694 | 0.869 | 0.903 |
| WRKY70 | WRKY40 | 3702.AT3G56400.1 | 3702.AT1G80840.1 | 0 | 0.249 | 0.85 |
| WRKY53 | WRKY33 | 3702.AT4G23810.1 | 3702.AT2G38470.1 | 0.64 | 0.8 | 0.849 |
| WRKY2 | ZAP1 | 3702.AT5G56270.1 | 3702.AT2G04880.1 | 0.778 | 0.064 | 0.833 |
| WRKY30 | WRKY53 | 3702.AT5G24110.1 | 3702.AT4G23810.1 | 0.786 | 0.494 | 0.809 |
| WRKY33 | WRKY15 | 3702.AT2G38470.1 | 3702.AT2G23320.1 | 0.707 | 0.71 | 0.744 |
| WRKY53 | WRKY40 | 3702.AT4G23810.1 | 3702.AT1G80840.1 | 0.634 | 0.658 | 0.74 |
| WRKY51 | WRKY70 | 3702.AT5G64810.1 | 3702.AT3G56400.1 | 0 | 0.275 | 0.692 |
| WRKY22 | WRKY40 | 3702.AT4G01250.1 | 3702.AT1G80840.1 | 0.674 | 0.6 | 0.685 |
| WRKY30 | WRKY70 | 3702.AT5G24110.1 | 3702.AT3G56400.1 | 0.712 | 0 | 0.635 |
| WRKY11 | WRKY22 | 3702.AT4G31550.1 | 3702.AT4G01250.1 | 0.723 | 0.549 | 0.622 |
| WRKY15 | WRKY40 | 3702.AT2G23320.1 | 3702.AT1G80840.1 | 0.683 | 0.545 | 0.611 |
| WRKY30 | WRKY40 | 3702.AT5G24110.1 | 3702.AT1G80840.1 | 0.644 | 0.472 | 0.581 |
| WRKY30 | WRKY33 | 3702.AT5G24110.1 | 3702.AT2G38470.1 | 0.652 | 0.493 | 0.579 |
| WRKY33 | WRKY6 | 3702.AT2G38470.1 | 3702.AT1G62300.1 | 0.651 | 0.45 | 0.576 |
| WRKY22 | WRKY33 | 3702.AT4G01250.1 | 3702.AT2G38470.1 | 0.678 | 0.442 | 0.571 |
| WRKY11 | WRKY33 | 3702.AT4G31550.1 | 3702.AT2G38470.1 | 0.699 | 0.456 | 0.563 |
| WRKY48 | WRKY40 | 3702.AT5G49520.1 | 3702.AT1G80840.1 | 0.695 | 0.489 | 0.559 |
| WRKY48 | WRKY33 | 3702.AT5G49520.1 | 3702.AT2G38470.1 | 0.696 | 0.455 | 0.544 |
| WRKY70 | WRKY33 | 3702.AT3G56400.1 | 3702.AT2G38470.1 | 0.643 | 0.345 | 0.532 |
| WRKY35 | WRKY9 | 3702.AT2G34830.1 | 3702.AT1G68150.1 | 0.66 | 0.354 | 0.528 |
| WRKY51 | WRKY41 | 3702.AT5G64810.1 | 3702.AT4G11070.1 | 0 | 0.136 | 0.526 |
| WRKY53 | WRKY15 | 3702.AT4G23810.1 | 3702.AT2G23320.1 | 0.652 | 0.408 | 0.507 |
| WRKY22 | WRKY15 | 3702.AT4G01250.1 | 3702.AT2G23320.1 | 0.732 | 0.438 | 0.505 |
| WRKY41 | WRKY40 | 3702.AT4G11070.1 | 3702.AT1G80840.1 | 0.634 | 0.321 | 0.485 |
| WRKY75 | WRKY6 | 3702.AT5G13080.1 | 3702.AT1G62300.1 | 0.845 | 0.397 | 0.475 |
| WRKY11 | WRKY40 | 3702.AT4G31550.1 | 3702.AT1G80840.1 | 0.674 | 0.322 | 0.467 |
| WRKY69 | WRKY65 | 3702.AT3G58710.1 | 3702.AT1G29280.1 | 0.901 | 0.43 | 0.465 |
| WRKY11 | WRKY53 | 3702.AT4G31550.1 | 3702.AT4G23810.1 | 0.642 | 0.334 | 0.463 |
| WRKY40 | WRKY6 | 3702.AT1G80840.1 | 3702.AT1G62300.1 | 0.729 | 0.329 | 0.462 |
| WRKY53 | WRKY22 | 3702.AT4G23810.1 | 3702.AT4G01250.1 | 0.663 | 0.3 | 0.437 |
| WRKY24 | WRKY43 | 3702.AT5G41570.1 | 3702.AT2G46130.1 | 0.971 | 0.437 | 0.437 |
| WRKY53 | WRKY6 | 3702.AT4G23810.1 | 3702.AT1G62300.1 | 0.64 | 0.238 | 0.435 |
| WRKY43 | WRKY56 | 3702.AT2G46130.1 | 3702.AT1G64000.1 | 0.971 | 0.429 | 0.435 |
| WRKY36 | WRKY9 | 3702.AT1G69810.1 | 3702.AT1G68150.1 | 0.742 | 0.435 | 0.435 |
| WRKY35 | WRKY36 | 3702.AT2G34830.1 | 3702.AT1G69810.1 | 0.642 | 0.434 | 0.434 |
| WRKY30 | WRKY41 | 3702.AT5G24110.1 | 3702.AT4G11070.1 | 0.784 | 0.352 | 0.433 |
| WRKY24 | WRKY56 | 3702.AT5G41570.1 | 3702.AT1G64000.1 | 0.969 | 0.424 | 0.424 |
| WRKY48 | WRKY30 | 3702.AT5G49520.1 | 3702.AT5G24110.1 | 0.658 | 0.325 | 0.423 |
| WRKY31 | WRKY14 | 3702.AT4G22070.1 | 3702.AT1G30650.1 | 0.651 | 0.222 | 0.4 |

**Table S4 Similarity based association of AtWRKYs with GgWRKYs in putative pathways**

| Biological process GO | | | | | | |  |
| --- | --- | --- | --- | --- | --- | --- | --- |
| pathway ID | **pathway description** | **Observed gene count** | | **false discovery rate** | **matching proteins in network (IDs)** | |  |
| GO.0006351 | transcription, DNA-templated | 47 | | 2.29E-54 | AT1G13960.1,AT1G18860.1,AT1G29280.1,AT1G29860.1,AT1G30650.1,  AT1G62300.1,AT1G64000.1,AT1G68150.1,AT1G69310.1,AT1G69810.1,  AT1G80840.1,AT2G03340.1,AT2G04880.1,AT2G23320.1,AT2G30590.1,  AT2G34830.1,AT2G37260.1,AT2G38470.1,AT2G44745.1,AT2G46130.1,  AT2G47260.1,AT3G01080.1,AT3G56400.1,AT3G58710.1,AT4G01250.1,  AT4G04450.1,AT4G11070.1,AT4G18170.1,AT4G22070.1,AT4G23810.1,  AT4G24240.1,AT4G26440.1,AT4G26640.2,AT4G30935.1,AT4G31550.1,  AT4G39410.1,AT5G13080.1,AT5G15130.1,AT5G24110.1,AT5G26170.1,  AT5G41570.1,AT5G45050.1,AT5G46350.1,AT5G49520.1,AT5G52830.1,  AT5G56270.1,AT5G64810.1 | |  |
| GO.0006355 | regulation of transcription, DNA-templated | 47 | | 3.03E-53 | AT1G13960.1,AT1G18860.1,AT1G29280.1,AT1G29860.1,AT1G30650.1,  AT1G62300.1,AT1G64000.1,AT1G68150.1,AT1G69310.1,AT1G69810.1,  AT1G80840.1,AT2G03340.1,AT2G04880.1,AT2G23320.1,AT2G30590.1,  AT2G34830.1,AT2G37260.1,AT2G38470.1,AT2G44745.1,AT2G46130.1,  AT2G47260.1,AT3G01080.1,AT3G56400.1,AT3G58710.1,AT4G01250.1,  AT4G04450.1,AT4G11070.1,AT4G18170.1,AT4G22070.1,AT4G23810.1,  AT4G24240.1,AT4G26440.1,AT4G26640.2,AT4G30935.1,AT4G31550.1,  AT4G39410.1,AT5G13080.1,AT5G15130.1,AT5G24110.1,AT5G26170.1,  AT5G41570.1,AT5G45050.1,AT5G46350.1,AT5G49520.1,AT5G52830.1,  AT5G56270.1,AT5G64810.1 | |  |
| GO.0010467 | gene expression | 47 | | 3.86E-48 | AT1G13960.1,AT1G18860.1,AT1G29280.1,AT1G29860.1,AT1G30650.1,  AT1G62300.1,AT1G64000.1,AT1G68150.1,AT1G69310.1,AT1G69810.1,  AT1G80840.1,AT2G03340.1,AT2G04880.1,AT2G23320.1,AT2G30590.1,  AT2G34830.1,AT2G37260.1,AT2G38470.1,AT2G44745.1,AT2G46130.1,  AT2G47260.1,AT3G01080.1,AT3G56400.1,AT3G58710.1,AT4G01250.1,  AT4G04450.1,AT4G11070.1,AT4G18170.1,AT4G22070.1,AT4G23810.1,  AT4G24240.1,AT4G26440.1,AT4G26640.2,AT4G30935.1,AT4G31550.1,  AT4G39410.1,AT5G13080.1,AT5G15130.1,AT5G24110.1,AT5G26170.1,  AT5G41570.1,AT5G45050.1,AT5G46350.1,AT5G49520.1,AT5G52830.1,  AT5G56270.1,AT5G64810.1 | |  |
| GO.0050794 | regulation of cellular process | 46 | | 8.71E-39 | AT1G13960.1,AT1G18860.1,AT1G29280.1,AT1G29860.1,AT1G30650.1,  AT1G62300.1,AT1G64000.1,AT1G68150.1,AT1G69310.1,AT1G69810.1,  AT1G80840.1,AT2G03340.1,AT2G04880.1,AT2G23320.1,AT2G30590.1,  AT2G34830.1,AT2G37260.1,AT2G38470.1,AT2G44745.1,AT2G46130.1,  AT2G47260.1,AT3G01080.1,AT3G56400.1,AT3G58710.1,AT4G01250.1,  AT4G04450.1,AT4G11070.1,AT4G18170.1,AT4G22070.1,AT4G23810.1,  AT4G24240.1,AT4G26440.1,AT4G26640.2,AT4G30935.1,AT4G31550.1,  AT4G39410.1,AT5G13080.1,AT5G15130.1,AT5G24110.1,AT5G26170.1, | |  |
| GO.0010243 | response to organonitrogen compound | 10 | | 3.78E-12 | AT1G62300.1,AT1G80840.1,AT2G23320.1,AT2G38470.1,AT3G56400.1,  AT4G01250.1,AT4G23810.1,AT4G26640.2,AT4G31550.1,AT5G49520.1 | |  |
| GO.1901700 | response to oxygen-containing compound | 18 | | 7.47E-12 | AT1G13960.1,AT1G62300.1,AT1G69310.1,AT1G80840.1,AT2G04880.1,  AT2G23320.1,AT2G38470.1,AT3G56400.1,AT4G01250.1,AT4G23810.1,  AT4G26640.2,AT4G31550.1,AT5G24110.1,AT5G26170.1,AT5G46350.1,  AT5G49520.1,AT5G52830.1,AT5G64810.1 | |  |
| GO.0010200 | response to chitin | 9 | | 8.30E-12 | AT1G62300.1,AT1G80840.1,AT2G23320.1,AT2G38470.1,AT3G56400.1,  AT4G01250.1,AT4G23810.1,AT4G31550.1,AT5G49520.1 | |  |
| GO.0010033 | response to organic substance | 18 | | 4.33E-09 | AT1G13960.1,AT1G62300.1,AT1G80840.1,AT2G04880.1,AT2G23320.1,  AT2G38470.1,AT2G47260.1,AT3G56400.1,AT4G01250.1,AT4G23810.1,  AT4G26640.2,AT4G31550.1,AT5G24110.1,AT5G26170.1,AT5G46350.1,  AT5G49520.1,AT5G52830.1,AT5G64810.1 | |  |
| GO.0042742 | defense response to bacterium | 9 | | 2.55E-08 | AT1G80840.1,AT2G38470.1,AT3G56400.1,AT4G23810.1,AT4G31550.1,  AT5G46350.1,AT5G49520.1,AT5G52830.1,AT5G64810.1, | |  |
| GO.0006950 | response to stress | 20 | | 4.23E-08 | AT1G13960.1,AT1G62300.1,AT1G69310.1,AT1G80840.1,AT2G38470.1,  AT3G56400.1,AT4G01250.1,AT4G23810.1,AT4G26440.1,AT4G26640.2,  AT4G31550.1,AT5G13080.1,AT5G15130.1,AT5G24110.1,AT5G26170.1,  AT5G45050.1,AT5G46350.1,AT5G49520.1,AT5G52830.1,AT5G64810.1 | |  |
| GO.0042221 | response to chemical | 19 | | 7.74E-08 | AT1G13960.1,AT1G62300.1,AT1G69310.1,AT1G80840.1,AT2G04880.1,  AT2G23320.1,AT2G38470.1,AT2G47260.1,AT3G56400.1,AT4G01250.1,  AT4G23810.1,AT4G26640.2,AT4G31550.1,AT5G24110.1,AT5G26170.1,  AT5G46350.1,AT5G49520.1,AT5G52830.1,AT5G64810.1 | |  |
| GO.0001101 | response to acid chemical | 13 | | 8.56E-08 | AT1G13960.1,AT1G69310.1,AT1G80840.1,AT2G04880.1,AT2G38470.1,  AT3G56400.1,AT4G23810.1,AT4G26640.2,AT5G24110.1,AT5G26170.1,  AT5G46350.1,AT5G52830.1,AT5G64810.1 | |  |
| GO.0009719 | response to endogenous stimulus | 16 | | 8.56E-08 | AT1G13960.1,AT1G62300.1,AT1G80840.1,AT2G23320.1,AT2G38470.1,  AT2G47260.1,AT3G56400.1,AT4G01250.1,AT4G23810.1,AT4G26640.2,  AT4G31550.1,AT5G26170.1,AT5G46350.1,AT5G49520.1,AT5G52830.1,  AT5G64810.1 | |  |
| GO.0006952 | defense response | 14 | | 1.46E-07 | AT1G13960.1,AT1G80840.1,AT2G38470.1,AT3G56400.1,AT4G01250.1,  AT4G23810.1,AT4G31550.1,AT5G15130.1,AT5G26170.1,AT5G45050.1,  AT5G46350.1,AT5G49520.1,AT5G52830.1,AT5G64810.1 | |  |
| GO.0050896 | response to stimulus | 23 | | 3.13E-06 | AT1G13960.1,AT1G62300.1,AT1G69310.1,AT1G80840.1,AT2G04880.1,  AT2G23320.1,AT2G38470.1,AT2G47260.1,AT3G56400.1,AT4G01250.1,  AT4G23810.1,AT4G26440.1,AT4G26640.2,AT4G31550.1,AT5G13080.1,  AT5G15130.1,AT5G24110.1,AT5G26170.1,AT5G45050.1,AT5G46350.1,  AT5G49520.1,AT5G52830.1,AT5G64810.1 | |  |
| GO.0009751 | response to salicylic acid | 6 | | 4.71E-06 | AT1G13960.1,AT1G80840.1,AT2G04880.1,AT3G56400.1,AT4G23810.1,  AT5G24110.1 | |  |
| GO.0045892 | negative regulation of transcription, DNA-template | 6 | | 6.48E-06 | AT1G62300.1,AT1G80840.1,AT3G56400.1,AT4G04450.1,AT5G13080.1,  AT5G52830.1 | |  |
| GO.0051707 | response to other organism | 11 | | 2.25E-05 | AT1G80840.1,AT2G38470.1,AT2G47260.1,AT3G56400.1,AT4G23810.1,  AT4G31550.1,AT5G26170.1,AT5G46350.1,AT5G49520.1,AT5G52830.1,  AT5G64810.1 | |  |
| GO.0098542 | defense response to other organism | 10 | | 2.58E-05 | AT1G80840.1,AT2G38470.1,AT3G56400.1,AT4G23810.1,AT4G31550.1,  AT5G26170.1,AT5G46350.1,AT5G49520.1,AT5G52830.1,AT5G64810.1 | |  |
| GO.0051704 | multi-organism process | 12 | | 2.96E-05 | AT1G80840.1,AT2G38470.1,AT2G47260.1,AT3G56400.1,AT4G23810.1,  AT4G26440.1,AT4G31550.1,AT5G26170.1,AT5G46350.1,AT5G49520.1,  AT5G52830.1,AT5G64810.1 | |  |
| GO.0080134 | regulation of sress response | 6 | | 3.22E-05 | AT1G13960.1,AT1G80840.1,AT3G01080.1,AT3G56400.1,AT4G23810.1,  AT5G46350.1 | |  |
| GO.0009605 | response to external stimulus | 12 | | 3.25E-05 | AT1G62300.1,AT1G80840.1,AT2G38470.1,AT2G47260.1,AT3G56400.1,  AT4G23810.1,AT4G31550.1,AT5G26170.1,AT5G46350.1,AT5G49520.1,  AT5G52830.1,AT5G64810.1 | |  |
| GO.0031347 | regulation of defense response | 5 | | 0.000209 | AT1G13960.1,AT1G80840.1,AT3G01080.1,AT3G56400.1,AT4G23810.1 | |  |
| GO.0048583 | regulation of response to stimulus | 7 | | 0.000209 | AT1G13960.1,AT1G80840.1,AT3G01080.1,AT3G56400.1,AT4G23810.1,  AT5G13080.1,AT5G46350.1 | |  |
| GO.0009867 | jasmonic acid mediated signaling pathway | 3 | | 0.00212 | AT3G56400.1,AT5G26170.1,AT5G64810.1 | |  |
| GO.0071395 | cellular response to jasmonic acid stimulus | 3 | | 0.00261 | AT3G56400.1,AT5G26170.1,AT5G64810.1 | |  |
| GO.0031325 | positive regulation of cellular metabolic process | 5 | | 0.00267 | AT2G04880.1,AT2G38470.1,AT4G23810.1,AT4G26640.2,AT5G49520.1 | |  |
| GO.0048518 | positive regulation of biological process | 7 | | 0.00297 | AT2G04880.1,AT2G38470.1,AT3G56400.1,AT4G23810.1,AT4G26640.2,  AT5G46350.1,AT5G49520.1 | |  |
| GO.0048519 | negative regulation of biological process | 7 | | 0.00406 | AT1G13960.1,AT1G62300.1,AT1G80840.1,AT3G56400.1,AT4G04450.1,  AT5G13080.1,AT5G52830.1 | |  |
| GO.0042542 | response to H2O2 | 3 | | 0.00541 | AT4G23810.1,AT5G24110.1,AT5G46350.1 | |  |
| GO.0050832 | defense response to fungus | 6 | | 0.00542 | AT1G80840.1,AT2G38470.1,AT3G56400.1,AT5G26170.1,AT5G46350.1,  AT5G64810.1 | |  |
| GO.0009753 | response to jasmonic acid | 4 | | 0.00651 | AT1G13960.1,AT3G56400.1,AT5G26170.1,AT5G64810.1 | |  |
| GO.0045893 | positive regulation of transcription, DNA-templated | 4 | | 0.00848 | AT2G04880.1,AT4G23810.1,AT4G26640.2,AT5G49520.1 | |  |
| GO.0009863 | salicylic acid mediated signaling pathway | 2 | | 0.037 | AT2G04880.1,AT3G56400.1 | |  |
| GO.0010193 | response to ozone | 2 | | 0.0397 | AT4G23810.1,AT5G24110.1 | |  |
| GO.1901701 | cellular response to oxygen-containing compound | 5 | | 0.0412 | AT2G04880.1,AT3G56400.1,AT5G26170.1,AT5G46350.1,AT5G64810.1 | |  |
| GO.0051716 | cellular response to stimulus | 10 | | 0.0492 | AT1G62300.1,AT2G04880.1,AT2G38470.1,AT3G56400.1,AT5G13080.1,  AT5G26170.1,AT5G45050.1,AT5G46350.1,AT5G52830.1,AT5G64810.1 | |  |
| MOLECULAR FUNCTION | | | | | | |  |
| GO.0043565 | sequence-specific DNA binding | | 47 | 1.71E-88 | | AT1G13960.1,AT1G18860.1,AT1G29280.1,AT1G29860.1,AT1G30650.1,  AT1G62300.1,AT1G64000.1,AT1G68150.1,AT1G69310.1,AT1G69810.1,  AT1G80840.1,AT2G03340.1,AT2G04880.1,AT2G23320.1,AT2G30590.1,  AT2G34830.1,AT2G37260.1,AT2G38470.1,AT2G44745.1,AT2G46130.1,  AT2G47260.1,AT3G01080.1,AT3G56400.1,AT3G58710.1,AT4G01250.1,  AT4G04450.1,AT4G11070.1,AT4G18170.1,AT4G22070.1,AT4G23810.1,  AT4G24240.1,AT4G26440.1,AT4G26640.2,AT4G30935.1,AT4G31550.1,  AT4G39410.1,AT5G13080.1,AT5G15130.1,AT5G24110.1,AT5G26170.1,  AT5G41570.1,AT5G45050.1,AT5G46350.1,AT5G49520.1,AT5G52830.1,  AT5G56270.1,AT5G64810.1 | |
| GO.0003700 | transcription factor activity, sequence-specific DNA binding | | 47 | 9.14E-64 | | AT1G13960.1,AT1G18860.1,AT1G29280.1,AT1G29860.1,AT1G30650.1,  AT1G62300.1,AT1G64000.1,AT1G68150.1,AT1G69310.1,AT1G69810.1,  AT1G80840.1,AT2G03340.1,AT2G04880.1,AT2G23320.1,AT2G30590.1,  AT2G34830.1,AT2G37260.1,AT2G38470.1,AT2G44745.1,AT2G46130.1,  AT2G47260.1,AT3G01080.1,AT3G56400.1,AT3G58710.1,AT4G01250.1,  AT4G04450.1,AT4G11070.1,AT4G18170.1,AT4G22070.1,AT4G23810.1,  AT4G24240.1,AT4G26440.1,AT4G26640.2,AT4G30935.1,AT4G31550.1,  AT4G39410.1,AT5G13080.1,AT5G15130.1,AT5G24110.1,AT5G26170.1,  AT5G41570.1,AT5G45050.1,AT5G46350.1,AT5G49520.1,AT5G52830.1,  AT5G56270.1,AT5G64810.1 | |
| GO.0003677 | DNA binding | | 44 | 8.81E-48 | | AT1G13960.1,AT1G18860.1,AT1G29280.1,AT1G29860.1,AT1G30650.1,  AT1G64000.1,AT1G68150.1,AT1G69310.1,AT1G69810.1,AT1G80840.1,  AT2G03340.1,AT2G04880.1,AT2G23320.1,AT2G30590.1,AT2G34830.1,  AT2G37260.1,AT2G38470.1,AT2G44745.1,AT2G46130.1,AT2G47260.1,  AT3G01080.1,AT3G56400.1,AT3G58710.1,AT4G01250.1,AT4G04450.1,  AT4G11070.1,AT4G18170.1,AT4G22070.1,AT4G24240.1,AT4G26440.1,  AT4G26640.2,AT4G30935.1,AT4G31550.1,AT4G39410.1,AT5G13080.1,  AT5G15130.1,AT5G24110.1,AT5G26170.1,AT5G41570.1,AT5G45050.1,  AT5G46350.1,AT5G52830.1,AT5G56270.1,AT5G64810.1 | |
| GO.0044212 | transcription regulatory region DNA binding | | 12 | 2.92E-16 | | AT1G13960.1,AT1G62300.1,AT1G68150.1,AT1G69310.1,AT1G80840.1,  AT2G23320.1,AT2G38470.1,AT4G04450.1,AT4G39410.1,AT5G13080.1,  AT5G24110.1,AT5G52830.1 | |
| GO.0005488 | binding | | 40 | 3.72E-16 | | AT1G13960.1,AT1G18860.1,AT1G29280.1,AT1G29860.1,AT1G30650.1,  AT1G64000.1,AT1G68150.1,AT1G69310.1,AT1G69810.1,AT1G80840.1,  AT2G03340.1,AT2G23320.1,AT2G34830.1,AT2G37260.1,AT2G38470.1,  AT2G44745.1,AT2G46130.1,AT2G47260.1,AT3G01080.1,AT3G56400.1,  AT3G58710.1,AT4G01250.1,AT4G04450.1,AT4G11070.1,AT4G18170.1,  AT4G22070.1,AT4G26440.1,AT4G26640.2,AT4G30935.1,AT4G39410.1,  AT5G13080.1,AT5G15130.1,AT5G24110.1,AT5G26170.1,AT5G41570.1,  AT5G45050.1,AT5G46350.1,AT5G52830.1,AT5G56270.1,AT5G64810.1 | |
| GO.0005516 | calmodulin binding | | 4 | 0.00596 | | AT2G23320.1,AT2G30590.1,AT4G24240.1,AT4G31550.1 | |
| CELLULAR COMPONENT GO | | | | | |  |  |
| GO.0005634 | nucleus | | 47 | 2.42E-40 | | AT1G13960.1,AT1G18860.1,AT1G29280.1,AT1G29860.1,AT1G30650.1,  AT1G62300.1,AT1G64000.1,AT1G68150.1,AT1G69310.1,AT1G69810.1,  AT1G80840.1,AT2G03340.1,AT2G04880.1,AT2G23320.1,AT2G30590.1,  AT2G34830.1,AT2G37260.1,AT2G38470.1,AT2G44745.1,AT2G46130.1,  AT2G47260.1,AT3G01080.1,AT3G56400.1,AT3G58710.1,AT4G01250.1,  AT4G04450.1,AT4G11070.1,AT4G18170.1,AT4G22070.1,AT4G23810.1,  AT4G24240.1,AT4G26440.1,AT4G26640.2,AT4G30935.1,AT4G31550.1,  AT4G39410.1,AT5G13080.1,AT5G15130.1,AT5G24110.1,AT5G26170.1,  AT5G41570.1,AT5G45050.1,AT5G46350.1,AT5G49520.1,AT5G52830.1,  AT5G56270.1,AT5G64810.1 |  |
| GO.0005622 | intracellular | | 47 | 1.25E-22 | | AT1G13960.1,AT1G18860.1,AT1G29280.1,AT1G29860.1,AT1G30650.1,  AT1G62300.1,AT1G64000.1,AT1G68150.1,AT1G69310.1,AT1G69810.1,  AT1G80840.1,AT2G03340.1,AT2G04880.1,AT2G23320.1,AT2G30590.1,  AT2G34830.1,AT2G37260.1,AT2G38470.1,AT2G44745.1,AT2G46130.1,  AT2G47260.1,AT3G01080.1,AT3G56400.1,AT3G58710.1,AT4G01250.1,  AT4G04450.1,AT4G11070.1,AT4G18170.1,AT4G22070.1,AT4G23810.1,  AT4G24240.1,AT4G26440.1,AT4G26640.2,AT4G30935.1,AT4G31550.1,  AT4G39410.1,AT5G13080.1,AT5G15130.1,AT5G24110.1,AT5G26170.1,  AT5G41570.1,AT5G45050.1,AT5G46350.1,AT5G49520.1,AT5G52830.1,  AT5G56270.1,AT5G64810.1 |  |
| GO.0005623 | cell | | 47 | 4.21E-20 | | AT1G13960.1,AT1G18860.1,AT1G29280.1,AT1G29860.1,AT1G30650.1,  AT1G62300.1,AT1G64000.1,AT1G68150.1,AT1G69310.1,AT1G69810.1,  AT1G80840.1,AT2G03340.1,AT2G04880.1,AT2G23320.1,AT2G30590.1,  AT2G34830.1,AT2G37260.1,AT2G38470.1,AT2G44745.1,AT2G46130.1,  AT2G47260.1,AT3G01080.1,AT3G56400.1,AT3G58710.1,AT4G01250.1,  AT4G04450.1,AT4G11070.1,AT4G18170.1,AT4G22070.1,AT4G23810.1,  AT4G24240.1,AT4G26440.1,AT4G26640.2,AT4G30935.1,AT4G31550.1,  AT4G39410.1,AT5G13080.1,AT5G15130.1,AT5G24110.1,AT5G26170.1,  AT5G41570.1,AT5G45050.1,AT5G46350.1,AT5G49520.1,AT5G52830.1,  AT5G56270.1,AT5G64810.1 |  |
| pathway ID/ pfam domain ID PF03106 | WRKY DNA -binding domain | | 22 | 1.88E-52 | | AT1G29280.1,AT1G29860.1,AT1G30650.1,AT1G62300.1,AT1G64000.1,  AT1G68150.1,AT1G69310.1,AT1G69810.1,AT1G80840.1,AT2G03340.1,  AT2G34830.1,AT2G37260.1,AT2G38470.1,AT2G44745.1,AT4G04450.1,  AT4G26440.1,AT4G26640.2,AT4G39410.1,AT5G26170.1,AT5G41570.1,  AT5G52830.1,AT5G64810.1 |  |

**Table S5 The AtWRKYs association as predicted by STRING**

| Cluster number | Cluster color | Gene count | Protein name (AtWRKY) | Protein identifier |
| --- | --- | --- | --- | --- |
| 1 | Red | 8 | WRKY30 | 3702.AT5G24110.1 |
| 1 | Red | 8 | WRKY15 | 3702.AT2G23320.1 |
| 1 | Red | 8 | WRKY48 | 3702.AT5G49520.1 |
| 1 | Red | 8 | WRKY22 | 3702.AT4G01250.1 |
| 1 | Red | 8 | WRKY40 | 3702.AT1G80840.1 |
| 1 | Red | 8 | WRKY53 | 3702.AT4G23810.1 |
| 1 | Red | 8 | WRKY11 | 3702.AT4G31550.1 |
| 1 | Red | 8 | WRKY33 | 3702.AT2G38470.1 |
| 3 | Brown | 3 | WRKY24 | 3702.AT5G41570.1 |
| 3 | Brown | 3 | WRKY56 | 3702.AT1G64000.1 |
| 3 | Brown | 3 | WRKY43 | 3702.AT2G46130.1 |
| 2 | Sandy Brown | 3 | WRKY36 | 3702.AT1G69810.1 |
| 2 | Sandy Brown | 3 | WRKY35 | 3702.AT2G34830.1 |
| 2 | Sandy Brown | 3 | WRKY9 | 3702.AT1G68150.1 |
| 6 | Cyan | 2 | ZAP1 | 3702.AT2G04880.1 |
| 6 | Cyan | 2 | WRKY2 | 3702.AT5G56270.1 |
| 7 | Dark Cyan | 2 | WRKY31 | 3702.AT4G22070.1 |
| 7 | Dark Cyan | 2 | WRKY14 | 3702.AT1G30650.1 |
| 8 | Cornflower Blue | 2 | WRKY69 | 3702.AT3G58710.1 |
| 8 | Cornflower Blue | 2 | WRKY65 | 3702.AT1G29280.1 |
| 4 | Green Yellow | 2 | WRKY75 | 3702.AT5G13080.1 |
| 4 | Green Yellow | 2 | WRKY6 | 3702.AT1G62300.1 |
| 5 | Green | 2 | WRKY51 | 3702.AT5G64810.1 |
| 5 | Green | 2 | WRKY41 | 3702.AT4G11070.1 |
| 9 | Blue | 1 | WRKY70 | 3702.AT3G56400.1 |

Table S6 Promoter sequences of WRKYs in G.glabra

| Gene ID | Gene Promoter | Sequence Information |
| --- | --- | --- |
| MN625704 | GgWRKY18 | GCTCGTAAGCGCGACCCTCTGTTGGTGGTAACAAAAGAAATTTAGGGTTTTCTTCTGAGCCCCCCCTTAGGGTTCCCTTCTTCCCCTGCCCCCTTTTCTTAATACCCAAACCAAGATCAAACAAATCTCATACCCGTTCACCAATCGCATAACAAAAAAAGAGGGACGAACAAATTAATTTGCAAAAACAGAAAACAAAAGAAGAAAGGGGGAAAAACCTAAAACCAGCGATGACGGGGAAAGCGAAGCCGAAGAAGCACACGGCGAAGGAGATCCAGGCGAAGGTGGACGCTGCGACGACGAATCGCGGTGGCGGAAAAGCTGGTCTTGCAGATCGTTCTGGAATCGAGAAGGGTGGGCACGCGAAGTGGGAGTGCCCTCACTGCAAAGTCACAGCACCAGATGTGAAATCGATGCAGATTCACCACGACGCTCGTCACCCTAAGATCCCCTTTGAGGAGGCCAAGATCGTCAACCTCCACGCCACTGTCGCCGTCGCCGACACTTCCTCCAAGCCTCGCCCTGGTATTCGCGGCAGCCTCAAGAAGTGATTATCACAATCACTTTTATTCGCCCCTTCATACATGCCTTTCACTATCCGTTCTCGTTTGTAATTTTTCATCTTTTAAGTTTACGTTTTTAGAGGGTTATCGAGTCATTTGGGACCTGGGTACGCTGCTTAGTTACTATTCCTTTTTCTATGGTGTGAGTTTGTGATGCGTTGTATGAAGTGGTCGAGAAGAAGGGTATTTTTTGGTGGGAAAATGGGATTGCTATTCATTCGGCCCTTCTTTTTAAGCCTGGAATCTATATTGTTCCCATGATGATGGCAATTAACGTGATGTTATCGAGTTACTACTATTACCACTGCTGTTGCTACTGCTCTCTTGTTGAATATTCTTTCCCTCTCTTGATGTAATACATGTGCTACTACGCTGGTCAGACCAATTTCATATTTCATATCACAATATACAAGGAGAGCGTTGTTACATCTCTAAATTAGTTTTGGTGCAACAGAACTCGAAAGACCACTTTTCTGATAATTGACAATGGCAATATTTAGTTATGTCTTCCTGGGCTGAGATTTGCTACTGTCTAAAAAATTGAATGAGTCACTGCAATAAGCGAATTGGAATCGGTGGATGAAGATTAGTGAAGATTTTTTATAAGTATTTGTTTAGATGTTAGTCATTCCAATTTCATTCAACGGCTCTAAGTCTGCACTATTTTTTGACTGTGTAATAAATCGAGTACATAAGTTCATGAGTAGGTTGGGAACTTTTCAAACTCTAATTCTTTGTTTGTTGAGTAATTTTAGACTGTTTGGATGATGAAGTGACGCGGTTGAAGAAGGTTTTTATAGCTATGTTCCTATTTCTTATTAGGTGAATCTTTGTCTTGATGGTATTGATATTCGGAACTTTGTTTGGGTATGACTGTATAATACCTATCCTGATCAATCTGTTTGCAGCCAACGGGAATGAATTTGTTATAAGTGCTGTCGTTTTTGCTGGAATAGATTAGTGATCTATATACCTTTATGTAGGATCATATGACTTTTTTCTTAATCGAATGTTTATTTCTGCAAAAAATATATTGGAGTTGCACTGTTGCGATTGCTTTTTCCTGGTTGGAGTTTGCTTATAAGTTCAATGTATCATCTGGTTTCGTCATTTCAATGACTTGAGAAAATACCCTTTTCCGAAGGCATTTGTTGCTGGGTCTTGTTTCCTGTAATTTATCCTCGGGTTAAGTATTGGAATTTCTAGACCAAATATCAGTCTCACATATATGTATTATGTAGTGATTCCTGCGCGTATTTACCAGTTTGCTGAGCACATTAGATTTTGGGATCCCTTTTATATACTCAATCTGTTTCATAGCATACTTTTTTTTTTATTTCTGCCAGAGATTGCATGTGCAATTGCTTCTCTTTTTGTTGCTAAGAAGTAAGTAGATATTATTGAATAGTTAACTGCATTGAGTGGTTTAACTCTCAAACTCCTATCACAGATTTTAAAATTTTTAGGTCGTTGTTATTTCTGTTTTTGCCTTATGTGTAAGTTTTACTTAATCTGGATTCCAACAGTAGAAAAAACACGCAATTGCCACAGTCAAGCATTTTGGATCGTTGAATTGAGATCACAACTCACGTTCTAAGTCTGAATTCTAAACCCGATTTACAAAGTCCAAACATGAGCTGTCAGATCTCAATCCAATATTCTGAGATGATTAATTGCGGCAACTGTGTGTTTTTGTACAATGGGGGATCTGGATCTAGTTTTATCTACAACAGGGGATCTGGATCTAAGTTTTACAAGTACTGCATACTCTTAATATTCCACTGAAAAAGCCAACGAGCAAACCTTGAAGGCAATCTAAGTATCTAACTACTGGTTAAGTGTTGAGAATCAAATGGCTATAAAGCCACTATGAGCAGGATGAATTGGAGTAGTTTATGGGAAATCATTAGCTTCAAATATTGATGATATTATTGTGA |
| MN625705 | GgWRKY19 | TGAAATTAACTTTCCGCAGTTACTTCTTTCTTTCAGGCAGTTTAGCAACCAATATGAACCGCCTCAAGGCTCCAACACACAGAATAGAAGAGCCCTTTTTACTCTCCTTAGATTAGTAGAACAACACCAATTCAATATCCTGTTCTTATATTTCCCCAAGACTCAGAATCAGTTTATCATTGAGAATAACGTCAGAGAATGAACCTAAATCACAGCACCAGCAAACACACACTCGAACTTATGGCATAACTATTGTTTCTATTTAAAAAGCAATAGTTCTGCAAAACATTTTCAATATTCAAATTAAGACAACTTTCAAAAAGACAACTTAAAAGCTTTAGTAGGCACAAAACAAGAATTACATGAGTCACAATATGTTGGAATTTAGATACATGTGTACCAAAATATACATCTTCACTTATTTATCCCTAATAATAATGATACCCAATGTAGTATTAATTGTGATTTGCATATTTAGTCAAACCGCCAATAGACAAGAAAAGAGTGAGCCAGAAGAAGGGAAAAGTAAAAGTTAGAATACAAATAGGAATCTTAATCCCACAACATATCTGTATATCCTATGTCATTGCCAACTTGTATGCTCTTCAGTAACTAGCTGCAGCTTCGTAAAACAATGCAAT |
| MN625706 | GgWRKY2 | GCACAAAGGTCTTAATTCCATTTTTTTTCCTTTACACTCTTTAAATAAGAAATCCGAGTTTCCACTTTCTACGAAGTGGACTTTGCGCTGAGTTCAAGATAACACAGACACACACGAAACCAAACCCCCATCATTTCGGTGCCACTATCCGATTCATTCATTCCCATTATTATTATTTTTATATCATTATTATTAATATCATCGTTGTCCAAAAATCGAAACCAACCCACCAACCGCCGCAAAAAAAATGTCCACCACCAGTGCCGGCGTCGATGCCGTGATGGCGGCAGCCCCCGCGCGCCCCACCATCACTCTCCCGCCGCGTCCCACCGCCGAGGCCTTCTTCGCCGGCGGCGGCGGTGTCAGTCCCGGCCCCATGACCCTCGTCTCCAGCTTCTTCGCCAACGACTCCTCCGCCACCTCCTTCTCGCAGCTCCTCGCCGGAGCCATGGCCTCCCCACTCTCCTTCGCCGCCGCCGGCGACAACAATTCCGGCAGGGAAGACGATGGGACCCACAAGGGCGTGGGGTTCAAGCAGAGCAGGCCCATGAACTTGGTCATTGCTCGTTCCCCTGTCTTCACTATCCCACCCGGTTTGAGCCCTTCAGGTTTTCTCAACTCCCCCGGTTTTTTTTCCCCACAGGTAAAATATTTGTGGCTGAATTCGAAAAAGGGGTGTGATTTTATGTGTCAAAGTTTCAATTTTTGTTTTCAATTATTTCGAAAGAGTGACACGTGATCGGTTGTTGTGGATGAGGCAATTCAGGGTGTTTATCAGTGTTTTGTAGGAAATTACTCAGTAATATACTCTCAATAATAATTCATATTTGATCTAAATTTTATCGTGTTATATTTCATTTAAAATTTAGTACAATTATATTGTGTGGTAGACACGTATGATGTTTGGGGCGATCCAAATAAACAAAGTATTTTTGTGTTATAGAAGGGATTGATGAAGCGATAGAGTTTAGATCGCATTTTAACAGAAACCGTATGGATATTATATATATCATATAGTAAAATGACTTCTGGTTGCGTTGCCTAGATTTTCTTGTATGATAATTTTGACGGGGTGGTTTAGCAACAATGAAAAATGAAGACACAGTTTGCGGCTGAAATTTTTATTTGAAATACAGTCTCTGCAAGTCACTTCATAATGAAAAAGAAAAAGAAATGAAATGTTTTTCCAAGTTTATATTAGCTAAGCAACTCAATGTATGGTTGTGCTTTAGCATTCTACTTGTTCATGACGACATGCTAAATTTCATAAATATTATTGTAGATCTGTTTTCCTCTTGTACAAAAATAAGAAGATTGTAAAGTAGTTCTTGCCGGGTAATTGTGCAGGGTCATGCATTGTATTACATCATTTGTTGGTCATGTTGCTGGGCATTTTTCTCATTATTATTTATCAATCTGATCCATTTCCCTTGAGGATGGTGGATATACTCGTGTGAGGTTTTCACCCTCTCTTCAATTCAATGTATACAGAGCCCCTTCGGG |
| MN625707 | GgWRKY22 | TGAAATTAACTTTCCGCAGTTACTTCTTTCTTTCAGGCAGTTTAGCAACCAATATGAACCGCCTCAAGGCTCCAACACACAGAATAGAAGAGCCCTTTTTACTCTCCTTAGATTAGTAGAACAACACCAATTCAATATCCTGTTCTTATATTTCCCCAAGACTCAGAATCAGTTTATCATTGAGAATAACGTCAGAGAATGAACCTAAATCACAGCACCAGCAAACACACACTCGAACTTATGGCATAACTATTGTTTCTATTTAAAAAGCAATAGTTCTGCAAAACATTTTCAATATTCAAATTAAGACAACTTTCAAAAAGACAACTTAAAAGCTTTAGTAGGCACAAAACAAGAATTACATGAGTCACAATATGTTGGAATTTAGATACATGTGTACCAAAATATACATCTTCACTTATTTATCCCTAATAATAATGATACCCAATGTAGTATTAATTGTGATTTGCATATTTAGTCAAACCGCCAATAGACAAGAAAAGAGTGAGCCAGAAGAAGGGAAAAGTAAAAGTTAGAATACAAATAGGAATCTTAATCCCACAACATATCTGTATATCCTATGTCATTGCCAACTTGTATGCTCTTCAGTAACTAGCTGCAGCTTCGTAAAACAATGCAATTTAGCTTTGGACAGGTTCTGCATCAGATGTTGACTGCTTACTAAATTCACTTATTGTGCTAGGAGTATCATGACCAGCAACCACATTAAGGCAATCCTTACTTCCTTCTGATGTAGTTTTTACCTCATCTTTCGCATGTTCATTTGATCTACATGGAACTTTGGAACTTGAATGGCATACCGTGTCATTGCTGACAGAATCGTCTTTCATATCTGAGTCTTCATTTTGTTGCTCAGGCTGACTCAATTTATTTTCAGGGCCCTCATTACGAAGGCTGATCACCCGAAACTCAGCCATATCACCAGCCTCTGAGTTAGTAATTGACTCCTCATTTGGTCGCTTATTTAATCTGCTTTTTGAGTCCAAACAGTTGCGTTCCCCTGTATCAACACAAACAGTATTACCTCCCGATTTGGTTCCCAGCTTGCCATCAATGTCCATTGTGTGTGTGTTTGTAGCAGCATTATGAGTGACACTCCTTCCAGAGGGGATTTCATGATCATGTTGTCCCTCATAGGTAGTTATGACAATTTTTGAATCATGAGAGGCTCTTTCAACATGCTTTTTGACAGGGCATCCAGGATTTGAACATCGATAATAACTTCTGGAAGAGTGAAGAGAAAATGCAGTCTATTAAATTAAATCATACACGAATTCATGTATCCATGAAAGCTCAGAGCAGAGCGAAAAAGAGAGACAAAAAAAGAGTATCTTCTCAATAGATCGAAAACTTGACAAGAGTAACATGTACTTAAAAGAGAAGAGACTGCAGTTAGACCAAATGGATTGGTCATTAGCAGGATCCACAATGTACTAATTAAGAACAAAAGCATTATTGATACTATTGTCATTTGTCATCGCTTTCTTTGTATACCAAAGCAACAAAAGAGACTTCTAATAAAATTGTTCACTCAAGTGCAAACATTTGAGGTCAGCTGGCATTTCACAACCCGGTTAGGACTGCTCAAAGCCATAAAAAACTGTGGTAGCAAGATAGGCAGAGTGCAGAACCAACCACAAATTATAATCCTTCAAAAAGTGCATGCAGAACACAATTTGAAAGCAATATAAAACGAACAAATGTAAACCCTTCCTGTTTCTTTCAAGGAGGGGAAGAATAATTCAACAGATCTTTAAGACTAGAAGCTAAGGCAATCAATTTATAGTAGCGAGATTTTAGG |
| MN625708 | GgWRKY31 | TAAGAAAAGACTATGTCAAAAAAGGAATAGAATGTCATTTGATTAAACAAATAAAACCAGTAAGTATTTATTTATAATATAAAAAGGGGTATGTGACATTTATGCACATCTAGGAGGACGTAATTACAATTTCTCCTTGCACTTTTTTTTTAACAGGGTAGGTTATTAAAGTCCCTGATGCAAGTTTTGCAGGCTTGCAGCGCATCTGTCTAGTCTTTCCAAGAAGTAAGTCCCTGACCTTCAGCAGAATCAGCACAAGCAGCTGATAGAACTTGTCATTAGAGTACATGTTCTTCCAAATAAAATGATGCCAATCAATTCTATATTCATTCCACTACTAGCAAACTGTTTCAGACAAGTTGAACAGGTGCACAAGCAAATACACACGAGCTGTTTCGCTAAAAGACTGACTACGCGCGAAGAAAAACAAGAAACAAGAAGAAAAGGATAAACATGGGAAGCTGTGAAATTACAAGATACCTGCCAATTAATTTATGTAGTTACAACTGCATGGTTTCTGAAAGATGAAT |
| MN625709 | GgWRKY41 | TAAGAAAAGACTATGTCAAAAAAGGAATAGAATGTCATTTGATTAAACAAATAAAACCAGTAAGTATTTATTTATAATATAAAAAGGGGTATGTGACATTTATGCACATCTAGGAGGACGTAATTACAATTTCTCCTTGCACTTTTTTTTAACAGGGTAGGTTATTAAAGTCCCTGATGCAAGTTTTGCAGGCTTGCAGCGCATCTGTCTAGTCTTTCCAAGAAGTAAGTCCCTGACCTTCAGCAGAATCAGCACAAGCAGCTGATAGAACTTGTCATTAGAGTACATGTTCTTCCAAATAAAATGATGCCAATCAATTCTATATTCATTCCACTACTAGCAAACTGTTTCAGACAAGTTGAACAGGTGCACAAGCAAATACACACGAGCTGTTTCGCTAAAAGACTGACTACGCGCGAAGAAAAACAAGAAACAAGAAGAAAAGGATAAACATGGGAAGCTGTGAAATTACAAGATACCTGCCAATTAATTTATGTAGTTACAACTGCATGGTTTCTGAAAGATGAATTCAACAAGGCTTGATTTCAAAACCTATGCTCAGAAGCGTTCGAGCTGACTCTATCGCTTTCTCACCCCCAAGGTCCAAAGCTTCCCCAGTTAGTTCTCCTTCAGTGTCCACTGACCATTGAGTAGGAGTTTTCTGGTTTTGCGGCGAGTCGGGCTTCTTCACCTGCAAATTATTCATGGAGGCAGGTGCTGCAGCAGCCACAAGAGGAGCACTTGGTGGGCCATGACGTTTCTTGGGCACAGGCATGTCATGATCATGTACCCCCTTGTATGTTATGATGACAGCATCTGAGTTGTCTACAGCGGTCTCAATGTGCTTTCGGACAGGACATCCAGCTGAAGTGCATCTGTAGTAGTTTCTGAAAATTCAAGCAGTTCATCAGAAGAAAAATAAAAGATTTCAATTTCAATGGAAAATTGAAGAAAACAAAAAGGAAAGTCAACAGCCCATATAAAAGAGATAAGGTGGGTTTAAGGGTCAGTTTGGTCACAGTTCAATATAATTCATAAAGACAAAAAAACAGAAAAAACAGTTTGGCAAATTATTTGTTGTTTGGAGATTTTAAACCTAGTTTCCAGAAATCTTTTCTTAAATTAAGACGGGAAAAAAGAGAAAATTTGAAGCTATTTTTCTTTTTCAGTTTTTAAAAGAATTGATTTGTACTTTGGCATCATTGTAAATCATGGAAAACTTGTGATTTGGATTGTAAAGACATGTCTTCATCTTCTCTTCTCCAAGCGTGTTTCATTTTTCCTCTGCCTGCTGATCTGCTCTTCTTCCCAGGGTATGCTACCAAGCCCTCGATAGTTGGGATTTCTCCATGCCAGCAGTGACAGCTTTCCACACCACTGTCTTGGCCTGCCGTGGCAAGACCTCACCCTACATTCCATGGCCGGCTATCCAGCAGATGAGGAAGTGCCTCTCAATGGACGACCACCACAAACCATCTTCATTTAAGCCATCATGGACTCACTAAGAAAGTGGCATTGTCTAGCATAGTGATATCTACCGCTAAAAGCACTCAAAATCTTGTACTACGGCGCAGCCAAGTTAGGATGAAGATTTTAGGAGATGAACAGCAGAAAATTCATATAAGCACTTTTTTTAAAAAAGTGTTTAGGAGATGTTTATCCAAACAATTTACTGACTCTATTATTTCAACAGTTTATCATGAAACAAGTTGTGAAATTAAGGTTAAAATCAGACTTTAAAAACAGAAAATAGAAAAACAATTCAAAATAGGCCACAAATTGCTAGCAAATTTTACTTTAGAGAAATGCTGCTTCATCACTAAGATATCTGTCCTTACCCTTTTCCCCATAGGAAGCAGCTCTTTTCAGCAAAGCACAGAAGCAAAAGGACTACACCAACAACACCACAAATTTGACCTCAGAAGATAAGAAATCTTATCCTCCCTGTTGTGGAAGGGATAAACTGATTACTATTCCTACTGCAAATGATGCAAACACAAACTAAGCATGCAAAATATTTTGAGAGAATATTTGCATTAAGAGGGTTTTGGACAATAAAAATGCGGATATATGTCCCAGTTGCTGATATTGTTGATTTCTAGCACAGAAAAAAAAAAGCAAAAATGCATCCGCAAGCTTATATAAATAAGATGCCTCATTGTCTTTCTTCAAACTGCTTACAAGCAAATAAACGCAGCTTGCAACTTTTTACTGATTTTATATTTTGTTTTGTTTTTTAAATTTTAAACACTCATTGAACCCAAATACAACTCAACTTTCACCATCTTCAATTAAAGATACTGAT |
| MN625710 | GgWRKY43 | TAAGAAAAGACTATGTCAAAAAAGGAATAGAATGTCATTTGATTAAACAAATAAAACCAGTAAGTATTTATTTATAATATAAAAAGGGGTATGTGACATTTATGCACATCTAGGAGGACGTAATTACAATTTCTCCTTGCACTTTTTTTTAACAGGGTAGGTTATTAAAGTCCCTGATGCAAGTTTTGCAGGCTTGCAGCGCATCTGTCTAGTCTTTCCAAGAAGTAAGTCCCTGACCTTCAGCAGAATCAGCACAAGCAGCTGATAGAACTTGTCATTAGAGTACATGTTCTTCCAAATAAAATGATGCCAATCAATTCTATATTCATTCCACTACTAGCAAACTGTTTCAGACAAGTTGAACAGGTGCACAAGCAAATACACACGAGCTGTTTCGCTAAAAGACTGACTACGCGCGAAGAAAAACAAGAAACAAGAAGAAAAGGATAAACATGGGAAGCTGTGAAATTACAAGATACCTGCCAATTAATTTATGTAGTTACAACTGCATGGTTTCTGAAAGATGAAT |
| MN625711 | GgWRKY9 | ACGATAATCACAAATTTGCACGTTAAACTGAATGAAAAAAAAAAACTACCATGTCCATAGCTAACTAGTACTGATCAATAAGGTCTTTTCGGGTGCTTTATGGTGATGCCAATGATTTACAATAAAAAACACTAGCATATGATACACTCCAGATTAAAAGAAGGATGGAAAAATTAATGTATAATTGTACATAATGACAAAATATGGGAGCAGATAGATTTCGTATACAAAGGCAGCTAGCAGAGGTGGAAAGTCATTGAAGGACAACGGTCCCACAATACTACTGATTCCTGAATACAAATTACCACCACCAATACTGTTATTATCAGTAGTTGCTGCATTAATCGAAACTGCCATCATCTTCTGATGATGAACTTCACTTATTATATATGAGCATAATTTAGGATAACGGGAATAGAACATTTCAAATGATGGGTTGTGTCAAGCCTATCAAAATTTGTGAAAATCTATCAAAAGAGAGAGACACACACAATATGTTTTCACATGGCAAGGTCATGTGAAAATCTATCATAAGAGGATCTATTCCCATTTAGTTATATATATACTGTTCTTGTTAAGTACTAAATAATTTTGAAGTGTCAATAC |
| MN625712 | GgWRKY77 | ATCGGTTTCCGGGGTTTTTTTTTTTTTGCTTTATTTTGAACCGCACATTTGTGTAGTACCGGCTGGAATTTATGGTGAGTGAAGTTCACTTGCTGAAAATGAATTGAAGTTCAATTAGATAGGTCATAAGACATAAGCCACCACCAAATGGCCATGGGTATATTCTTACTTATGTTTTTGTTGCATGGTTTTGACAGCAAAGGTGCATTATGGAACTTCTTTGTCCAACGTGGACTGGTTACATGGCGGGGCTGAGTCCCTTCTCACTCTCACCAATCTGTTCATTGTGCTGGGTCTGAGAGAGGGACTCAGAAAATCTGGAAATTCAGAGAAAAACACACCCACTCCAAACCCGGGTTCGAAGGAGGAGAAGAAACGTGGGATGAAGGTTTGATATTTGGAGTCATTCTTTCTATTACCATTTAGCAGTGTGGTATTATCATTTGCTCTATCGGTTCGGCTCAGATGTTGCTCCAAATTTTGGCTGGGATTTATAATCATTGCCTTAAAAATCTCTAACTAATCGTTGCAGCCTGTGGGATATTTTGTAAATAAGATGGATATAAATGTTGGATTTTGTGTAATTTATCATTAATTGGAATGAAATCTTACAAAGCTTTGGTTAGTATATCCAATTTTGATCGCAATTTTTGGTCTGTTCCATTACATGTTACATGAATAACGACTTGCCCTTCAATAATGATATAAAAGCTATCTTTTAATATTGATCCTTTTTTGGATGATGTAGTTAAATTATGTAACTTAATTCTCTTTGGTTAATTGCTCTCATTATTATGACTGGCATCCTCTTATACTAAAAATGTAAGTCATATAGGCTTTACCAATGCAAATTTGGGGCACCTTCTTATTTGAAAGTAAATACTGCTAAACAGTGCTAAAATTGTAATTCTAATATTAACTACAAGGACGTTTATAAATAGCACTAAGCTATCATTGCTGATTCGTTGTTCTACAATTCTGCTACTTTCTGAAGTTGTCCTATTTTAGTATAACTTCATACACATAATATTCAAACATGATTTTCCATGTTATTTGATCCATGTAGCCTACCTTACTCACTGAAAAAGGCTTCGGTTGTTGATGATGTTGTTTATACTCCTAATATTCCATTTATTCTTAAGTACAC |
| MN625713 | GgWRKY70 | ACACTTTTTTCTTTTTGAGATTTCAACCATGACAATTGACAAGTGATCCAACCAAAAATACTACCATTTTCACAGGTATTTTTCATTCATTTAGAAAAACCTAAAAATGTTCATAACACAATCAAGAATGACGAAAATAGTTCATACGAATCGAAACTTCCAATTTTACAGATTTCATCATTTCATTTTACCATATATCCTCATCCCCTCTCTCTGACTAAACGGCTCATTAGCCAACTAACCAAAAGCCGAAAAAACTGAGCGAAGAAAACCCCTTGCTTGCACCACTTAAAATATGTACAGCATCATCTCAAGCCAATAAGAGGTTTTGATAAATTGAATCATGGGGGATTGATCCTGGTTAAGCTCTCTCAATGTAGTTTCATGCATTTGCAGATTGTGTTGGTAATTTGGGGTGGTTGTGGTCACCTTCATAGGTAACTATTAGCATGGTAGGCTCTTCCAAGCACCTCTCAACATGCTTCCTAGCAGGGCAGCCTCTCATGCTGCTGCACTTATAGTATCCCCTGAAAAAACAAGAGGAGGAATTACAATCAATTACCTCAAAAATAAAATCTACTGTTTACATTCAAAGATTAAACTTAACATGTGGCATATCTAATAGGAAATCTAGTTAATGACACCCTGAGTGATAAAGAGACATTTGTGATAGTCCCCTTCATCTTTTTTCATATAAATTGAAGGTGTTTATGTTGTCACCTCCAAAATACCAGATATTAATTTCTCATTCATGTATTCACTTTTCAGAAACAGAAGAGCCATGAAAGAGAGTCCTATAATTAATCTACAAAAATTCACCACATGGAACTGTGAATGCAATATTTGTAATAACAGATGAAGAGAAGCTAGTGAATTTTTCAGAAGAAAAATCTAGGACCTTTTCAATCACTCATGTCTGCAATGTTCTGATCCACTGAATACCTCCTAAGAACAAGTTACACATGCAGAAGTATGCAATATAGCAACTCAAATTGAAGCACAATCACTACCACTCACACCTTGTGTTGGCTCACTATATGCAGTTCTAAACTTATATTGTCCTTGCTTAATCCATTTTTAACTAATGTGAGACTTCATCACTCAAATTTGAATTTCCAACAAAACAAACCGCACAATAATCAATA |
| MN625714 | GgWRKY37 | TATCCGGGATTGATAACTATGCTTAAAAGAAATAACTACTCTAAATTGTCCTTCATAAGGAATAAGATATCTTGAATCATGGTAGTAGCATTATGCAGCAACTTGCAGCTGACTGTCCTTTGTGAAGGATTTTTAGCTCTTATTTTAGCATGTTTTATTATCTACTGGTTCCCTTTTCTTTTGCCGAAAGTTTGCTTCATTCTCCCCTTAGCTGGTTCTCATTTTTTTTGTCATTAAGAACAATGGATGGATGACAAGGATAATTGTTTTTTTTTTCAAGTAAATATTTTTTCCATGTGTTTTAGAATAAAAACTACTGTTTACACCAAATTTAGGATTAAAATCAGTTTTTTAAACAGTATCTTACAAAAGGCTAGTATTTAAAACCAAATTCAGAAGCAAAACCGGGCTTCAAAACCAGTTTCCATAAAAACTGGTTCTACAACAAAAACCAGTTCCGAAACCAGTTTAGTCCTAAACTGATTCACAAAACCGGAAACCGGTTCGACCAAAAGAAACCAGTTCTGTTTAAAAACCAGACCACAAAACAAAGTGGTTAAGTTTGGTTCAGGTTCTTGGACCATGCATAGCCCTAGTGGCAGCTGGCAACCCCTCTCGCTATAAGCTCCGGACCTTTCCACTCACAGATACCTTCTCTCGGTCTCTCTCTCTATGTGTGTGTGTCTATATATATATAGTTTCTTAGTGCCCTTGACCTTTCAGAAACATGTATGTACTGAACAATATGAAACTCCTTGTGAACATTTTTTAAATATAGGATGAAAACATGCAGCTATTTCTTAGAGCTTACAATGATCTTTATTTGATAATTATAAGGTGGAAACTCTTCAGAAAGAGTTACAACATGTGAGAAAGGAGAACAATACTCTAAGACTG |
| MN625715 | GgWRKY73 | AGCTGGTGTTATTCCACGATGAAGTCTATAAAGATGCACATGAGAAGAGTTCCGGTCACAGCTTGGAACTCCTAAACCCTTTCGTGCACATGATGGCATTATCCGGAAGATATTAATATTATTTCCATAAATCGATGCAGTGACCAGAAGTGTCCCACTAGGGTCAAAACATAGAGCAGATATAGGACTAGAGTGAGCTTTAAATTGTGAGATGATAGCTCTGGAAGCAAAATCCTTAATAATTACCTGCAATAGACCATATGAACCAAGAACAAGTAAGAGTTAATATTCTATTCTAAAGCATAAAACTTTCTAACTTATTAATCAGCTGAGCCATGATGAAACAATACCAACCGTTCCTGCATTATCCACATCATTTCCTGTAACCCTACTAACTTTCCAGCCAGAATTTGATGGTACTGGAGAACTAGAACCATCAGGAAGCAACTCGTGACAGTATTTGATTATTCCAGCAGCCAAATGTTTACTGGACTCCATTGCATAGCGAGCCACCAAACTACCACTGCTCGGAGATGTTGATGGACTAACTCCTGGAGACGGACTTAGGTTTTGTGGGCTTATGCATCCTACATTTGAGGGCAGCGGGTTGTTGGAAGCATATGCTAGCCACCTTGGACCCACAGCCATTGGACCATAACCCACATTAACTCCCATTGTTCCTTGTCCCGCTAACTGAGGAACAGGATAAGTGAGAACACTGAATTTATTCTCAAGAGTCAATGCATCGAAGCAGTATATTTGTGTGGCAAGACCAACAGCTACTATCTTTGAACTGCATCTGATCATACACACCATGGATCGAAATCTCAGAACATGAACATAACAGTGAGACTTCAAGGAGTAAAACCGAACAGCTGTTGCAGAGTTGACACCATTCCCAGATTGAGTCTCTACCTTACCATCTCGTCCTAAACTACCCAAATGGTTACTGTTTTGGCTAACACCGCCAGCATCATCCCCGGCAACAACCAGGAGCAATGGATGAGATTTTCTGAATCCCTCCTGGCAGTCATCAGAACCTACAGGGAAAGGCTGCATCTGTAAGAAAGAAACTGGCCCGTCGCGCTTTGAGACCAGCTCACTAAAGCCAGAGGCATCCTCCACATCAAGTACTTGAAATCCGTTCAGATAACCAAGTAAAAGAACACGTTTAAAGATAGACTGATCAAGCTCTAGTACATCGAAACCAGCCCAGGTAACCTGATCTTTGTGATCATCGGAGGCAGAGGAAATGGAAGCGGCGACGGAGGCACCGGCGGAGCGGACGGTGGAAGCGACGGTGCTGGCATTGGTGGAGACGGTCTTAAGGCAGGAAGAGATGATCCTCAACGAACTCGGCAACAAGCCGTTGTTCTTCCCCTTCCCTTTCTTCATCGTGTTGATTATTATTATAATAATATTATTATCCTTCAACTACTACTCTTCCCACCGACAACCACAACCACGCGTCGGCAACAAAAAGAGAAACAAAGGAAAAAAAGAAAAAACGTTTCGGATTGCAATCACCATCGCAGACGCTTTGAGAGTGTTTGAGTTTGCTAACAGCGTCGCGGTGGCAGACTCAGAGTTGAGTACTACTGCGGACTTTGGTTTCTCTTTCTCTATCGTCCATCGTTGCTGGTGACTGTTACACTACTCCTCCTCCTCCTCCTCCTCCTCAGAATAACAATGAATTAGCAGCACATGGTTCTATCAAGTTTTCGGTTTCTGCTGCAAACTCATCATTTGTGTCCACCTTAACCGGGGACAGTGAAAATTTGCAACAACGACCCTGTCGTTCTTCTTCTGGGTTCCAAATAAGCCACGTGTCACTTCAGGGGTCTTCTTAT |
| MN625716 | GgWRKY11 | GGGAGCCTCGTGCACTGAGCCGCCTTTTTTTAAATTGGATAATCTGTGGTCACTTCTTCAATTTGAGTAAGGGTTTTATTAGGTGATCTCAAGTTTTCCTGTGACCTTATCTGATTTCACTTCTGGGACTTCTTGCTTGTACTTTTTTCCTTTATTTTTTTGTCTTTTTTTTAACTTCTCAAATTTCATTAGAGCATGCTGTTTGGCTAATAATATGATAGCTCTTTTCTTGAATATGGTTCTACTGTAACCATTATGCTCCTACTGATTTATTAATCTGTCTAATTTCAGGCACAACCATCTCCAACAACTGGAAAGTTTCCATTTGTCTCAAATGGCAACATTCGGTGCTCAGAATTATCATCTGATGCTCCAGAAAAATGTAAAGATAATAACTTTGATGATATTTATGCATCATCCTTTGCTTTCAAGCCCACAAATGATTCGGGCTCCTCTTTTTATCATGGTGCTGGTAGAAAGATAAATCCAACTACACTTCCTCAGCAATCCCTTCCTGGTATTGAAGCTTCAGTTCAGTCAGAAAATTCTTTTCAATCTCAAAGTGCCGAAGCAATCAAAGTTCAAACCGAGAACAAAAGTGGCCTCCATCTTCAGGCAGACTTCACCGAACTGCCACCTCAAAAGGACAATGGAATTAAGACGTACTTGGCTGATCAAAGGGCTTTTGATACTGTTGGTGGTAGCATTGAACATTCTACACCACTTGAAGTTCAGGCAGATGAAGAGGGAGATCAAAGAGTCAACGGAGACTCATTGGCTGCTGGTGCTGGTGGTACTCCGTCTGAAGATGGATATAACTGGAGAAAATATGGGCAGAAGCAAGTTAAGGGTAGCGAGTTCCCGCGGAGTTACTACAAGTGTACGCATCCAAATTGCTCGGTTAAAAAGAAAGTTGAACGTTCTCACGAGGGACACATCACAGAGATCATCTATAAGGGAGCACACAACCATCCAAAACCTCCTCCAAGTCGTCGGTCAGGC |
| MN625717 | GgWRKY13 | TTTGATATCTGATCAGATTCTAGGAGAATTATCTTTCCCTTGATTCAGCAGATTGGTTTCCTTGTTTAGGACCAATTCTACCTTTATATTATGATGCTGTACTGATCGATTTTCTCATTGGAGAATAATAAAGAAAATTCCAATCTGTTCTTTTAGTACACCCAATCATCAAGTGCTGTGCAGTTATGTGTGGTTGTTAACAATTCAAGTTTAGATATGTTCTGATGGACATTGTATGGGATTTGTAGTGAGTTGATGGTTATATCTAGGCAATGATATGTAGATCTATATCTTCTTTTTGGTTATTTTGTCTGCCTTCTGTTTGCAGATAAATCCAACTACACTTCCTCAGCAATCCCTTCCTGGTATTGAAGCTTCAGTTCAGTCAGAAAATTCTTTTCAATCTCAAAGTGCCGAAGCAATCAAAGTTCAAACCGAGAACAAAAGTGGCCTCCATCTTCAGGCAGACTTCACCGAACTGCCACCTCAAAAGGACAATGGAATTAAGACGTACTTGGCTGATCAAAGGGCTTTTGATACTGTTGGTGGTAGCATTGAACATTCTACACCACTTGAAGTTCAGGCAGATGAAGAGGGAGATCAAAGAGTCAACGGAGACTCATTGGCTGCTGGTGCTGGTGGTACTCCGTCTGAAGATGGATATAACTGGAGAAAATATGGGCAGAAGCAAGTTAAGGGTAGCGAGTTCCCGCGGAGTTACTACAAGTGTACGCATCCAAATTGCTCGGTTAAAAAGAAAGTTGAACGTTCTCACGAGGGACACATCACAGAGATCATCTATAAGGGAGCACACAACCATCCAAAACCTCCTCCAAGTCGTCGGTCAGGC |
| MN625718 | GgWRKY49 | GACTTCAACAACCACGTAAGTGAACAATCTACTCAAGTAGAAGATCCAGGAAAAGGTCAATCATTTGCATCCTCACCATTAATCGAAAGTGAGAGAGCAGTTCCATCTAATGAATTAAGCTTATCATCACCTGGTCAAATGGTTAGTTCTGGGGCTAGTGCTCCTGTTGATGCCGATTTGGATGAATTTAACCACAAAGGCAACACAGCTACTGGGCCTCAAACATCACATGTTGAGGTTAGAGGCAGTGGACTTTCAGTTGCAGCCGAGAAAGTATCAAATGATGGATACAATTGGCGAAAATATGGGCAAAAACTTGTTAAAGGAAGTGAATTTCCACGCAGCTATTACAAATGTACGTATCCTAACTGTGAAGTGAAAAAACTTTTTGAACGTTCTCATGACGGTCAAATCACTGAGATAATTTACAAGGGAACACATGATCATCCTAAACCTCAACCAAGCAACCGATACTCTGTTGGTGCTGTTATGTCTATGCAAGGAGAGAGATCTGATAAGGCTTTTTTGGCTGGCCGAGACGGTAATACCAGCCAGATGCTTCTTGAGTTCTCGTTGCTTCCATTTTATTATTACTTATTGCAATGAAAATAATCTGGCTTTCTTTATCTTCTAATTCTTGAATTGTAGACAAAGCATCCAAT |
| MN625719 | GgWRKY60 | AGAGAGAGAGAGAGAGAGAGATCCAATGTTACAAGTCAACTGCGTCTCACTCTCTGACACACACAACCTTCAAACACAGACACACACAAAATACGCACACTCCAGAGTCCCTTATTTGAAATGACCCTTTTGCCCCTCCCTTTCTGCCTCGTTCATCTTCCGTTTTGACTCAACCACTCTCACTTTCACTCTCACACGCACGCGCACACTCTCTCTCTCTCTTACCACAATGGACGCCGGCGAAGCACACTCTGGGTCGGAGCTCCGGCGAAGGGGTCCAGGCGAGGCGGAATCGGAACATGAGGATCCGAATCGAACCGGTGGGTATCGACCCGATTCTGGCGCTGACGGAGCTGCGGCTTCCGGCACCACCGTCGGCGCTAGGTACAAGCTGATGTCGCCGGCGAAGCTCCCGATCTCTAGGTCGCCGGTTTTGACGATTCCGCCGGGGCTCAGTCCGACGGCGTTTCTGGAATCCCCGGTTCTGCTCTCAAACATGAAGGTGGAACCTTCACCAACAACAGGGTCCCTTCCTAGGCTTCAACAAACAGCACATGGTTCTGTGACTTCTGCTACATCTGCTGCATTTCCTGTAACCACTGTATGCTTCAATACCAATACTTTGGATGACGGAAAATCCAGCTTCTTTGAGTTCAAACCACACAGTAGATCAAATATGGTTCCTGCTGAATTAGACAACCGTGCATGTGAAAAATCTACTCAAATAGATGGTCAAGGAAAAGCTCAATCTTTTGCCTCATCACCATTAGTCAAAAGTGAAATTGCAGGCCCTTCAAATGAAATAAGTTTATCATCACCTGTTCAGATGGTTAGCTCAGGGGCTAGTGCTCATGTTGAAGTCGATTTGGACAAATTAAACTCTGGGGGCAACATAGCAACTGGGCTTCAAGTATCACAAGTTGAGGGTAGGGGCAGTGGACTCTCAGTTGCTGCCGAGAGATCATCTGATGATGGATACAACTGGCGAAAATATGGGCAAAAGCACGTTAAAGGAAGTGAATTTCCACGTAGTTATTACAAATGCACTCACCCTAATTGTGAAGTGAAAAAACTATTTGAACGCTCTCATGATGGTCAAATCACTGAGATAGTTTACAAGGGAACACATGATCATCCTAAACCTCAACCAAACCGCCGATACTCTGGTGGAAATATTATGTCTATGCAAGAAGAGAGATCTGATAAGGCTTCTTTGACTAGCCGAGATGGTAATGCCAGATGAATACTTCTCATTGATTCCATTTTATTCTTACTTAGTGCTAAGCAAATAATGTGGCTTTCTTTTTCTTCTAATTCATGAATTGTAGACAGAGGATATAATAATTATGGTCAGCCTTCTCACACAGCTGAGCCTGACGGCACTCCAGAGCTATCGCCTGTAGCAACAAATGATGATAGTCTAGAGGATGCAGGGTTTTTGTCAAACCGGAATAACGATGAGGTTGATGAAGATGATCCCTTCTCAAAGCGAAGGTTTGACAATTTAGGCCAAAGCTATAGCTTTTGCTGCTTGTATTTACTTGCTGATTACAAGTTTTCTGTTGAGCAGAAAA |
| MN625720 | GgWRKY80 | AGGTGGAACCTTCACCAACAACAGGGTCCCTTCCTAGGCTTCAACAAACAGCACATGGTTCTGTGACTTCTGCTACATCTGCTGCATTTCCTGTAACCACTGTATGCTTCAATACCAATACTTTGGATGACGGAAAATCCAGCTTCTTTGAGTTCAAACCACACAGTAGATCAAATATGGTAATGAATTACTTGATGTTAGTAAAACTGATAAATATAAACTTTTGACTAAGGTGGAGACTTGGACTTTATTTTTTTCCTCCTTTAATTTATCTGATCGTAACCTGTGTACTAAGCTAACAAAATAGGATGATTAAGTAATATAATCTTTTCACTAGTTCCTTTATTTTTCCAGGGGGTTGTAAAGTGTTAGATGGAAACATATTTGATTGCCATGTATAACGTTCTGGTTAATATTGTTTTGGATATTACTGGTTGCATAGTACTTACAGAATGCTATTCACTTATTTAAGGTTCCTGCTGAATTAGACAACCGTGCATGTGAAAAATCTACTCAAATAGATGGTCAAGGAAAAGCTCAATCTTTTGCCTCATCACCATTAGTCAAAAGTGAAATTGCAGGCCCTTCAAATGAAATAAGTTTATCATCACCTGTTCAGATGGTTAGCTCAGGGGCTAGTGCTCATGTTGAAGTCGATTTGGACAAATTAAACTCTGGGGGCAACATAGCAACTGGGCTTCAAGTATCACAAGTTGAGGGTAGGGGCAGTGGACTCTCAGTTGCTGCCGAGAGATCATCTGATGATGGATACAACTGGCGAAAATATGGGCAAAAGCACGTTAAAGGAAGTGAATTTCCACGTAGTTATTACAAATGCACTCACCCTAATTGTGAAGTGAAAAAACTATTTGAACGCTCTCATGATGGTCAAATCACTGAGATAGTTTACAAGGGAACACATGATCATCCTAAACCTCAACCAAACCGCCGATACTCTGGTGGAAATATTATGTCTATGCAAGAAGAGAGATCTGATAAGGCTTCTTTGACTAGCCGAGATGACAGAGGATATAATAATTATGGTCAGCCTTCTCACACAGCTGAGCCTGACGGCACTCCAGAGCTATCGCCTGTAGCAACAAATGATGATAGTCTAGAGGATGCAGGGTTTTTGTCAAACCGGAATAACGATGAGGTTGATGAAGATGATCCCTTCTCAAAGCGAAGGTTTGACAATTTAGGCCAAAGCTATAGCTTTTGCTGCTTGTATTTACTTGCTGATTACAAGTTTTCTGTTGAGCAGAAAA |
| MN625721 | GgWRKY23 | TGAAATTAACTTTCCGCAGTTACTTCTTTCTTTCAGGCAGTTTAGCAACCAATATGAACCGCCTCAAGGCTCCAACACACAGAATAGAAGAGCCCTTTTTACTCTCCTTAGATTAGTAGAACAACACCAATTCAATATCCTGTTCTTATATTTCCCCAAGACTCAGAATCAGTTTATCATTGAGAATAACGTCAGAGAATGAACCTAAATCACAGCACCAGCAAACACACACTCGAACTTATGGCATAACTATTGTTTCTATTTAAAAAGCAATAGTTCTGCAAAACATTTTCAATATTCAAATTAAGACAACTTTCAAAAAGACAACTTAAAAGCTTTAGTAGGCACAAAACAAGAATTACATGAGTCACAATATGTTGGAATTTAGATACATGTGTACCAAAATATACATCTTCACTTATTTATCCCTAATAATAATGATACCCAATGTAGTATTAATTGTGATTTGCATATTTAGTCAAACCGCCAATAGACAAGAAAAGAGTGAGCCAGAAGAAGGGAAAAGTAAAAGTTAGAATACAAATAGGAATCTTAATCCCACAACATATCTGTATATCCTATGTCATTGCCAACTTGTATGCTCTTCAGTAACTAGCTGCAGCTTCGTAAAACAATGCAAT |
| MN625722 | GgWRKY28 | TGAAATTAACTTTCCGCAGTTACTTCTTTCTTTCAGGCAGTTTAGCAACCAATATGAACCGCCTCAAGGCTCCAACACACAGAATAGAAGAGCCCTTTTTACTCTCCTTAGATTAGTAGAACAACACCAATTCAATATCCTGTTCTTATATTTCCCCAAGACTCAGAATCAGTTTATCATTGAGAATAACGTCAGAGAATGAACCTAAATCACAGCACCAGCAAACACACACTCGAACTTATGGCATAACTATTGTTTCTATTTAAAAAGCAATAGTTCTGCAAAACATTTTCAATATTCAAATTAAGACAACTTTCAAAAAGACAACTTAAAAGCTTTAGTAGGCACAAAACAAGAATTACATGAGTCACAATATGTTGGAATTTAGATACATGTGTACCAAAATATACATCTTCACTTATTTATCCCTAATAATAATGATACCCAATGTAGTATTAATTGTGATTTGCATATTTAGTCAAACCGCCAATAGACAAGAAAAGAGTGAGCCAGAAGAAGGGAAAAGTAAAAGTTAGAATACAAATAGGAATCTTAATCCCACAACATATCTGTATATCCTATGTCATTGCCAACTTGTATGCTCTTCAGTAACTAGCTGCAGCTTCGTAAAACAATGCAATTTAGCTTTGGACAGGTTCTGCATCAGATGTTGACTGCTTACTAAATTCACTTATTGTGCTAGGAGTATCATGACCAGCAACCACATTAAGGCAATCCTTACTTCCTTCTGATGTAGTTTTTACCTCATCTTTCGCATGTTCATTTGATCTACATGGAACTTTGGAACTTGAATGGCATACCGTGTCATTGCTGACAGAATCGTCTTTCATATCTGAGTCTTCATTTTGTTGCTCAGGCTGACTCAATTTATTTTCAGGGCCCTCATTACGAAGGCTGATCACCCGAAACTCAGCCATATCACCAGCCTCTGAGTTAGTAATTGACTCCTCATTTGGTCGCTTATTTAATCTGCTTTTTGAGTCCAAACAGTTGCGTTCCCCTGTATCAACACAAACAGTATTACCTCCCGATTTGGTTCCCAGCTTGCCATCAATGTCCATTGTGTGTGTGTTTGTAGCAGCATTATGAGTGACACTCCTTCCAGAGGGGATTTCATGATCATGTTGTCCCTCATAGGTAGTTATGACAATTTTTGAATCATGAGAGGCTCTTTCAACATGCTTTTTGACAGGGCATCCAGGATTTGAACATCGATAATAACTTCTTGGATTTGCATTCCCCTTAACCATTTTCTGCCCGTATTTGCGCCAGCGATACCCATCATTTACAAAATCAACCTCACTTGAAGTATGAACAGCAACACGAGATTCACAAGTTGACATGTCAACTCCAGTGACATCCACGTTACTACTGTCTTTCTTCCTGTTTGGGATCAATTTGGCAGTTAAATAAAGGTTAACTATCACATATATATATCAACCCCAACAATATTTTAGAAAACAGTGAAATAACGTACAGCCGCTTTGACTCAGGATCCTCCAAGTTATGAACCTCATACTTTGCTGTCAATTGTACATGTGCAGCTTTCAACTTGTCAGCTCCGGATACTTTTGAAGGTGGAAGGGAATTTAAAGGCTTGATCTGTTGAGGCATACATCCATGGTCAACGGATGATTTGTCTGCATGCATGATACCATGAAATATATCAAGAGCTTTATCATATAAGGATGGAAACTAAAACACTAACAAAAT |
| MN625723 | GgWRKY42 | TAAGAAAAGACTATGTCAAAAAAGGAATAGAATGTCATTTGATTAAACAAATAAAACCAGTAAGTATTTATTTATAATATAAAAAGGGGTATGTGACATTTATGCACATCTAGGAGGACGTAATTACAATTTCTCCTTGCACTTTTTTTTAACAGGGTAGGTTATTAAAGTCCCTGATGCAAGTTTTGCAGGCTTGCAGCGCATCTGTCTAGTCTTTCCAAGAAGTAAGTCCCTGACCTTCAGCAGAATCAGCACAAGCAGCTGATAGAACTTGTCATTAGAGTACATGTTCTTCCAAATAAAATGATGCCAATCAATTCTATATTCATTCCACTACTAGCAAACTGTTTCAGACAAGTTGAACAGGTGCACAAGCAAATACACACGAGCTGTTTCGCTAAAAGACTGACTACGCGCGAAGAAAAACAAGAAACAAGAAGAAAAGGATAAACATGGGAAGCTGTGAAATTACAAGATACCTGCCAATTAATTTATGTAGTTACAACTGCATGGTTTCTGAAAGATGAATTCAACAAGGCTTGATTTCAAAACCTATGCTCAGAAGCGTTCGAGCTGACTCTATCGCTTTCTCACCCCCAAGGTCCAAAGCTTCCCCAGTTAGTTCTCCTTCAGTGTCCACTGACCATTGAGTAGGAGTTTTCTGGTTTTGCGGCGAGTCGGGCTTCTTCACCTGCAAATTATTCATGGAGGCAGGTGCTGCAGCAGCCACAAGAGGAGCACTTGGTGGGCCATGACGTTTCTTGGGCACAGGCATGTCATGATCATGTACCCCCTTGTATGTTATGATGACAGCATCTGAGTTGTCTACAGCGGTCTCAATGTGCTTTCGGACAGGACATCCAGCTGAAGTGCATCTGTAGTAGTTTCTGGGATGTGGATTCCCCTTCACCATTTTCTGTCCATACTTGCGCCATCGGTATCCATCACCTGATATCCCGACATCCCCTGCTGCATGTACGACAAGCTTGGGTTTCTTTTCAAGTTTTACAAGAGAATCCGAGTCCGTTAAGTCACCTTTCTCCTTTCTGGTTATCAAATTAAAGGAGAGAACATAAATCATTATATCCTGCCACAATTAGAAGAAATTGGCTTATTGTCTACACTTTTTTCTATAAAAAATATGATT |
| MN625724 | GgWRKY62 | TAAGAAAAGACTATGTCAAAAAAGGAATAGAATGTCATTTGATTAAACAAATAAAACCAGTAAGTATTTATTTATAATATAAAAAGGGGTATGTGACATTTATGCACATCTAGGAGGACGTAATTACAATTTCTCCTTGCACTTTTTTTTAACAGGGTAGGTTATTAAAGTCCCTGATGCAAGTTTTGCAGGCTTGCAGCGCATCTGTCTAGTCTTTCCAAGAAGTAAGTCCCTGACCTTCAGCAGAATCAGCACAAGCAGCTGATAGAACTTGTCATTAGAGTACATGTTCTTCCAAATAAAATGATGCCAATCAATTCTATATTCATTCCACTACTAGCAAACTGTTTCAGACAAGTTGAACAGGTGCACAAGCAAATACACACGAGCTGTTTCGCTAAAAGACTGACTACGCGCGAAGAAAAACAAGAAACAAGAAGAAAAGGATAAACATGGGAAGCTGTGAAATTACAAGATACCTGCCAATTAATTTATGTAGTTACAACTGCATGGTTTCTGAAAGATGAATTCAACAAGGCTTGATTTCAAAACCTATGCTCAGAAGCGTTCGAGCTGACTCTATCGCTTTCTCACCCCCAAGGTCCAAAGCTTCCCCAGTTAGTTCTCCTTCAGTGTCCACTGACCATTGAGTAGGAGTTTTCTGGTTTTGCGGCGAGTCGGGCTTCTTCACCTGCAAATTATTCATGGAGGCAGGTGCTGCAGCAGCCACAAGAGGAGCACTTGGTGGGCCATGACGTTTCTTGGGCACAGGCATGTCATGATCATGTACCCCCTTGTATGTTATGATGACAGCATCTGAGTTGTCTACAGCGGTCTCAATGTGCTTTCGGACAGGACATCCAGCTGAAGTGCATCTGTAGTAGTTTCTGAAAATTCAAGCAGTTCATCAGAAGAAAAATAAAAGATTTCAATTTCAATGGAAAATTGAAGAAAACAAAAAGGAAAGTCAACAGCCCATATAAAAGAGATAAGGTGGGTTTAAGGGTCAGTTTGGTCACAGTTCAATATAATTCATAAAGACAAAAAAACAGAAAAAACAGTTTGGCAAATTATTTGTTGTTTGGAGATTTTAAACCTAGTTTCCAGAAATCTTTTCTTAAATTAAGACGGGAAAAAAGAGAAAATTTGAAGCTATTTTTCTTTTTCAGTTTTTAAAAGAATTGATTTGTACTTTGGCATCATTGTAAATCATGGAAAACTTGTGATTTGGATTGTAAAGACATGTCTTCATCTTCTCTTCTCCAAGCGTGTTTCATTTTTCCTCTGCCTGCTGATCTGCTCTTCTTCCCAGGGTATGCTACCAAGCCCTCGATAGTTGGGATTTCTCCATGCCAGCAGTGACAGCTTTCCACACCACTGTCTTGGCCTGCCGTGGCAAGACCTCACCCTACATTCCATGGCCGGCTATCCAGCAGATGAGGAAGTGCCTCTCAATGGACGACCACCACAAACCATCTTCATTTAAGCCATCATGGACTCACTAAGAAAGTGGCATTGTCTAGCATAGTGATATCTACCGCTAAAAGCACTCAAAATCTTGTACTACGGCGCAGCCAAGTTAGGATGAAGATTTTAGGAGATGAACAGCAGAAAATTCATATAAGCACTTTTTTTAAAAAAGTGTTTAGGAGATGTTTATCCAAACAATTTACTGACTCTATTATTTCAACAGTTTATCATGAAACAAGTTGTGAAATTAAGGTTAAAATCAGACTTTAAAAACAGAAAATAGAAAAACAATTCAAAATAGGCCACAAATTGCTAGCAAATTTTACTTTAGAGAAATGCTGCTTCATCACTAAGATATCTGTCCTTACCCTTTTCCCCATAGGAAGCAGCTCTTTTCAGCAAAGCACAGAAGCAAAAGGACTACACCAACAACACCACAAATTTGACCTCAGAAGATAAGAAATCTTATCCTCCCTGTTGTGGAAGGGATAAACTGATTACTATTCCTACTGCAAATGATGCAAACACAAACTAAGCATGCAAAATATTTTGAGAGAATATTTGCATTAAGAGGGTTTTGGACAATAAAAATGCGGATATATGTCCCAGTTGCTGATATTGTTGATTTCTAGCACAGAAAAAAAAAAGCAAAAATGCATCCGCAAGCTTATATAAATAAGATGCCTCATTGTCTTTCTTCAAACTGCTTACAAGCAAATAAACGCAGCTTGCAACTTTTTACTGATTTTATATTTTGTTTTGTTTTTTAAATTTTAAACACTCATTGAACCCAAATACAACTCAACTTTCACCATCTTCAATTAAAGATACTGATTTACCAAGGAAAAATTATGAGATTCATGTTAAGAACAAATAAAAAAAAAGAAAGCAAGCTATATATAGGAATGCACCTGGGATGTGGATTCCCCTTCACCATTTTCTGTCCATACTTGCGCCATCGGTATCCATCACCTGATATCCCGACATCCCCTGCTGCATGTACGACAAGCTTGGGTTTCTTTTCAAGTTTTACAAGAGAATCCGAGTCCGTTAAGTCACCTTTCTCCTTTCTGGTTATCAAATTAAAGGAGAGAACATAAATCATTATATCCTGCCACAATTAGAAGAAATTGGCTTATTGTCTACACTTTTTTCTATAAAAAATATGATT |
| MN625725 | GgWRKY74 | ACGATAATCACAAATTTGCACGTTAAACTGAATGAAAAAAAAAAACTACCATGTCCATAGCTAACTAGTACTGATCAATAAGGTCTTTTCGGGTGCTTTATGGTGATGCCAATGATTTACAATAAAAAACACTAGCATATGATACACTCCAGATTAAAAGAAGGATGGAAAAATTAATGTATAATTGTACATAATGACAAAATATGGGAGCAGATAGATTTCGTATACAAAGGCAGCTAGCAGAGGTGGAAAGTCATTGAAGGACAACGGTCCCACAATACTACTGATTCCTGAATACAAATTACCACCACCAATACTGTTATTATCAGTAGTTGCTGCATTAATCGAAACTGCCATCATCTTCTGATGATGAACTTCACTTATTATATATGAGCATAATTTAGGATAACGGGAATAGAACATTTCAAATGATGGGTTGTGTCAAGCCTATCAAAATTTGTGAAAATCTATCAAAAGAGAGAGACACACACAATATGTTTTCACATGGCAAGGTCATGTGAAAATCTATCATAAGAGGATCTATTCCCATTTAGTTATATATATACTGTTCTTGTTAAGTACTAAATAATTTTGAAGTGTCAATACTCAACATGGTTTATCTTTGCTAAGAGAAGCTTGTGAATCTTTTTCAGAGGCCACACTACTTCCTGTGTTCTTGATCAGTGGCATCTCATGGTTGTGCTTTCCTTCATATGTAGTCACAAATGATCTCGGGTCATCCATTGCTCTCTCCACGTGCTTGCGAACGTTACACTTCACATTAGTACATCTATAATAACTTCTGGGATATGGATTGCCTTTCACCACTTTCTGCCCATATTTCCTCCAGCGAAAGCCATCTCCAAGTATTTCAGAATCCATAGAGCTTTGCATCACAACGCGTGGCTCTACTAAACCTTCCTCTGATGCTGCCGCTTCATTGGATTGGTTCTCATTTTTCCTGAAGGATATGAATGAACCCTTTAATTAAAAATTGACCATGAGTATGACAAGGTAGTTAGCTAGCTCAAAAATTAGAATTACTAATTATGTTATTACTGTATGGACAGATTTTGTTTTTGTTAATGATTTTTATATATCTTTTCTAAATCACCCTTTAGACAAGTTCTTTGGAATAGCAACATGGACAATGCACAAACTTAGGTACATAAGTCCTGAAATTATGTAACTAACTAATAATTTGTGCCACATAACATGAAGTTATATATTGCAAACTCAAGCAGCTACCTTCCTTTTTGCTTTAGACATGGCAAGAGTTT |
| MN625726 | GgWRKY78 | GCTCGTAAGCGCGACCCTCTGTTGGTGGTAACAAAAGAAATTTAGGGTTTTCTTCTGAGCCCCCCCTTAGGGTTCCCTTCTTCCCCTGCCCCCTTTTCTTAATACCCAAACCAAGATCAAACAAATCTCATACCCGTTCACCAATCGCATAACAAAAAAAGAGGGACGAACAAATTAATTTGCAAAAACAGAAAACAAAAGAAGAAAGGGGGAAAAACCTAAAACCAGCGATGACGGGGAAAGCGAAGCCGAAGAAGCACACGGCGAAGGAGATCCAGGCGAAGGTGGACGCTGCGACGACGAATCGCGGTGGCGGAAAAGCTGGTCTTGCAGATCGTTCTGGAATCGAGAAGGGTGGGCACGCGAAGTGGGAGTGCCCTCACTGCAAAGTCACAGCACCAGATGTGAAATCGATGCAGATTCACCACGACGCTCGTCACCCTAAGATCCCCTTTGAGGAGGCCAAGATCGTCAACCTCCACGCCACTGTCGCCGTCGCCGACACTTCCTCCAAGCCTCGCCCTGGTATTCGCGGCAGCCTCAAGAAGTGATTATCACAATCACTTTTATTCGCCCCTTCATACATGCCTTTCACTATCCGTTCTCGTTTGTAATTTTTCATCTTTTAAGTTTACGTTTTTAGAGGGTTATCGAGTCATTTGGGACCTGGGTACGCTGCTTAGTTACTATTCCTTTTTCTATGGTGTGAGTTTGTGATGCGTTGTATGAAGTGGTCGAGAAGAAGGGTATTTTTTGGTGGGAAAATGGGATTGCTATTCATTCGGCCCTTCTTTTTAAGCCTGGAATCTATATTGTTCCCATGATGATGGCAATTAACGTGATGTTATCGAGTTACTACTATTACCACTGCTGTTGCTACTGCTCTCTTGTTGAATATTCTTTCCCTCTCTTGATGTAATACATGTGCTACTACGCTGGTCAGACCAATTTCATATTTCATATCACAATATACAAGGAGAGCGTTGTTACATCTCTAAATTAGTTTTGGTGCAACAGAACTCGAAAGACCACTTTTCTGATAATTGACAATGGCAATATTTAGTTATGTCTTCCTGGGCTGAGATTTGCTACTGTCTAAAAAATTGAATGAGTCACTGCAATAAGCGAATTGGAATCGGTGGATGAAGATTAGTGAAGATTTTTTATAAGTATTTGTTTAGATGTTAGTCATTCCAATTTCATTCAACGGCTCTAAGTCTGCACTATTTTTTGACTGTGTAATAAATCGAGTACATAAGT |
|  |  | TCATGAGTAGGTTGGGAACTTTTCAAACTCTAATTCTTTGTTTGTTGAGTAATTTTAGACTGTTTGGATGATGAAGTGACGCGGTTGAAGAAGGTTTTTATAGCTATGTTCCTATTTCTTATTAGGTGAATCTTTGTCTTGATGGTATTGATATTCGGAACTTTGTTTGGGTATGACTGTATAATACCTATCCTGATCAATCTGTTTGCAGCCAACGGGAATGAATTTGTTATAAGTGCTGTCGTTTTTGCTGGAATAGATTAGTGATCTATATACCTTTATGTAGGATCATATGACTTTTTTCTTAATCGAATGTTTATTTCTGCAAAAAATATATTGGAGTTGCACTGTTGCGATTGCTTTTTCCTGGTTGGAGTTTGCTTATAAGTTCAATGTATCATCTGGTTTCGTCATTTCAATGACTTGAGAAAATACCCTTTTCCGAAGGCATTTGTTGCTGGGTCTTGTTTCCTGTAATTTATCCTCGGGTTAAGTATTGGAATTTCTAGACCAAATATCAGTCTCACATATATGTATTATGTAGTGATTCCTGCGCGTATTTACCAGTTTGCTGAGCACATTAGATTTTGGGATCCCTTTTATATACTCAATCTGTTTCATAGCATACTTTTTTTTTTATTTCTGCCAGAGATTGCATGTGCAATTGCTTCTCTTTTTGTTGCTAAGAAGTAAGTAGATATTATTGAATAGTTAACTGCATTGAGTGGTTTAACTCTCAAACTCCTATCACAGATTTTAAAATTTTTAGGTCGTTGTTATTTCTGTTTTTGCCTTATGTGTAAGTTTTACTTAATCTGGATTCCAACAGTAGAAAAAACACGCAATTGCCACAGTCAAGCATTTTGGATCGTTGAATTGAGATCACAACTCACGTTCTAAGTCTGAATTCTAAACCCGATTTACAAAGTCCAAACATGAGCTGTCAGATCTCAATCCAATATTCTGAGATGATTAATTGCGGCAACTGTGTGTTTTTGTACAATGGGGGATCTGGATCTAAGTTTTATCTACAACAGGGG |
| MN625727 | GgWRKY57 | GCACAAAGGTCTTAATTCCATTTTTTTTCCTTTACACTCTTTAAATAAGAAATCCGAGTTTCCACTTTCTACGAAGTGGACTTTGCGCTGAGTTCAAGATAACACAGACACACACGAAACCAAACCCCCATCATTTCGGTGCCACTATCCGATTCATTCATTCCCATTATTATTATTTTTATATCATTATTATTAATATCATCGTTGTCCAAAAATCGAAACCAACCCACCAACCGCCGCAAAAAAAATGTCCACCACCAGTGCCGGCGTCGATGCCGTGATGGCGGCAGCCCCCGCGCGCCCCACCATCACTCTCCCGCCGCGTCCCACCGCCGAGGCCTTCTTCGCCGGCGGCGGCGGTGTCAGTCCCGGCCCCATGACCCTCGTCTCCAGCTTCTTCGCCAACGACTCCTCCGCCACCTCCTTCTCGCAGCTCCTCGCCGGAGCCATGGCCTCCCCACTCTCCTTCGCCGCCGCCGGCGACAACAATTCCGGCAGGGAAGACGATGGGACCCACAAGGGCGTGGGGTTCAAGCAGAGCAGGCCCATGAACTTGGTCATTGCTCGTTCCCCTGTCTTCACTATCCCACCCGGTTTGAGCCCTTCAGGTTTTCTCAACTCCCCCGGTTTTTTTTCCCCACAGGTAAAATATTTGTGGCTGAATTCGAAAAAGGGGTGTGATTTTATGTGTCAAAGTTTCAATTTTTGTTTTCAATTATTTCGAAAGAGTGACACGTGATCGGTTGTTGTGGATGAGGCAATTCAGGGTGTTTATCAGTGTTTTGTAGGAAATTACTCAGTAATATACTCTCAATAATAATTCATATTTGATCTAAATTTTATCGTGTTATATTTCATTTAAAATTTAGTACAATTATATTGTGTGGTAGACACGTATGATGTTTGGGGCGATCCAAATAAACAAAGTATTTTTGTGTTATAGAAGGGATTGATGAAGCGATAGAGTTTAGATCGCATTTTAACAGAAACCGTATGGATATTATATATATCATATAGTAAAATGACTTCTGGTTGCGTTGCCTAGATTTTCTTGTATGATAATTTTGACGGGGTGGTTTAGCAACAATGAAAAATGAAGACACAGTTTGCGGCTGAAATTTTTATTTGAAATACAGTCTCTGCAAGTCACTTCATAATGAAAAAGAAAAAGAAATGAAATGTTTTTCCAAGTTTATATTAGCTAAGCAACTCAATGTATGGTTGTGCTTTAGCATTCTACTTGTTCATGACGACATGCTAAATTTCATAAATATTATTGTAGATCTGTTTTCCTCTTGTACAAAAATAAGAAGATTGTAAAGTAGTTCTTGCCGGGTAATTGTGCAGGGTCATGCATTGTATTACATCATTTGTTGGTCATGTTGCTGGGCATTTTTCTCATTATTATTTATCAATCTGATCCATTTCCCTTGAGGATGGTGGATATACTCGTGTGAGGTTTTCACCCTCTCTTCAATTCAATGTATACAGAGCCCCTTCGGG |
| MN625728 | GgWRKY10 | TGGTGAATTGGGAAATTTTGGGCTCTTTTTGACGAGGCTTGAAATTTGCGGTAAAAGTACAAATCAAGATTGCCTACCGGTAAAAAAACAAAGTTATTAAGTTATATAGTAGTCGAGGGATCGACATCATGTGATGAAAATAGGACAACTGATAGTTTGAAGAGTTTGTATATTCAATCCAATTGACTAAAGTCAGAATATGAATCACACCAATCTCTTTTAACGATGCTGATCTCTTGACTCCAGCGAATTTGTGCTAGCAATCCTAATTTCGTGCGAAACTACTCATCTTGTAATTCACCCTCTAATTGGGTAGATTGACACGATTATATCCAGACCGTACGTAATAAACACCACAAGCTAGCGTATCTGATTGATTTTCCTTATGCGTTATATTCACCTCCATGATCAATTATTTACAGTCAATAGTGATAGCCCGATTTGTATATTATTTGGAAACATAATACCTATCAAAATCCTTTGTTATGGAGAGAAAAGAAGCGAGTTCCTTCTGTTTATTTGTATATGAGAATCAGTACAAATTAAATGTGTACTACAAAAAGCGGCAGAAACTATAGTTATGATTTAGTACTAACTTACTTGGTAAGGAAAATTAATAACCATTCGTAGATGGAGTACTACTACTATATTAAGCATTTTCTCATGATAATTCACTCTAATATTGTGGAATGCCTCATAACACTTGGTAAGAGCTAGCACATGCAGTTACGAGAACTAGAGGTTGCTATAGTCCACGAAACCGCCAAAACTTG |
| MN625729 | GgWRKY36 | TATACATTTAAACACTAAAGGGGGAGATGTAAAAGTAGGACGAATAAAAGCAAGAAGAAAAACAAAGTTCAAAACCAAAAGCTCCCTTTCCCATATATAACAAGCAACCATCTAAATTAAGTCTTAGCTGAAATTTCATTCTCAGAAAAGAAAAAAAAAAGCTAACTTTATCTCTTTTTTTCCTAGACCTCTTCCACCTTTTCCCTCTCTCTCTCTCTTGTTTCTTCTCAATTCCATGGCGAATGAGAAGGACCGTTATTACAATCCCTTCTATGATCACAACCACCATGATGATGAACTCAACAACAACAATCACTCAAACAATATTCCATTCTTTACCTCACAACAAAACTTTGAAGGATTATTAATGGATCCCTCTCACACCACAAGTTTCAGTGACTACTTACATGGTAGTTCCATGGACTACAACACCCTCTCAAGAGCATTTGACTTGTCTTGCTCATCATCATCTCATGAAGTGAAACCCAGTAATAATAGTACTACTGCAGCTGCAGGAGACTCACCACTAGTGGGGAGGAACATCAATAGTGAGAACCAATCAACAACACTCAATTCCTCAGTGTCTTCCTCATCTAATGAAGAAGCTGAAGAAGAAGACTCAATTACCAAAAGCAACAAGAAAGATAAGCAGCAGCCAACAAAAGGGTGTACTGAAGATGGAGATCATGATGATCAAAAGTCTAAGAAAGAGTAGGTTTAATTTTTCCACCTCTTCTTTGTGTACCTCAATTTCTTCTTGCTTTTCTGGTAGATCGATGATCAATGGAGGGTAAGTAAGAAATTTTTTCCTCGAATATTAGATTCTCTTTTTCTTCGTTTTTTTTTATAAGCAAAAAGATGGCATTAATTGGAGTACAAAGGATACCTCAATCCTTACAACAGAACTCATACATCAAAAAGAACTCAAACCAGCAACATACAATTGAATCAATTGATGAGTTGGAATAGGATCTGATTATATATCTTAATAACTTATGTTATTTTCTTTGAGGTAGAATTTTGAATTACAAATGCAATTATATGAGTATCACCTACCTAGTTAGTTAATTAAAATTAATATTGTCTTTTCAACTATTTGTGATGTTTTCAGTGTCTGATCTTTATGATCAAATGAGTTTGGTCAATCTTGCCTATACTCTATCTCTTAATATACTATACCGTTCTGTGAATGTTGTGTAGGTTTTGTTCATATATGACTAAAATAGTGGAACCAACAAGGATAGGCATACATATAGCCAG |
|  |  |  |
| MN625730 | GgWRKY47 | TAAGAAAAGACTATGTCAAAAAAGGAATAGAATGTCATTTGATTAAACAAATAAAACCAGTAAGTATTTATTTATAATATAAAAAGGGGTATGTGACATTTATGCACATCTAGGAGGACGTAATTACAATTTCTCCTTGCACTTTTTTTTAACAGGGTAGGTTATTAAAGTCCCTGATGCAAGTTTTGCAGGCTTGCAGCGCATCTGTCTAGTCTTTCCAAGAAGTAAGTCCCTGACCTTCAGCAGAATCAGCACAAGCAGCTGATAGAACTTGTCATTAGAGTACATGTTCTTCCAAATAAAATGATGCCAATCAATTCTATATTCATTCCACTACTAGCAAACTGTTTCAGACAAGTTGAACAGGTGCACAAGCAAATACACACGAGCTGTTTCGCTAAAAGACTGACTACGCGCGAAGAAAAACAAGAAACAAGAAGAAAAGGATAAACATGGGAAGCTGTGAAATTACAAGATACCTGCCAATTAATTTATGTAGTTACAACTGCATGGTTTCTGAAAGATGAAT |
| MN625731 | GgWRKY38 | CCTCTCTCTCTCTCTCTCTCAAGTCTATATCTCTTTTCTCTCTCTCTCTCTGATTCCATTCAAATTGATGGCCAGAGGGGGTGGACTCTCCATTGATTCAGATCCAACTGGGAGCTTCTTCCACCACAAGCCAATAGTTCTCAACTCTTTTCCTGAAGACAACAACAACATCATCAGCAGTAACAGCCATCATCCCAAGTGGAAACTTGGTCACAATATGGATGGCACAGTTAATAAGAGGTCTTCTAATTCATCCAATACTCCCAACAGCACCACCATACCCTTCCAAGTCAACCTTAGCTGTACCCACGACAGCCATGCCTCTTCTCCACCCTCTGATCATGATCACAACAACAACAACAGACCCCTCATCGATGAAATGGATTTCTTCTCTTCTCATCACAATAAGAACAATGACAACTTTGCCTCTGCCTCTACCTCTGCACCTCACACACATGATCACCACTCCCCCACTCCTGCTATCTTGGAATTGAAAGTAAACACTGGTCTGAATCTTCTTACCACCAACACTAGCAGTGATCAATCCATGGTGGAGGATGACATATCATCAAATTCACAAGACAAAAAAGCTAAGCTTGAGCTGGTTGTTCTTAAAGCTGAGCTAGAGAGGATGAAGGTGGAGAATCATCGACTGAGGAACATGCTTGATGAGGCTAACACAAACTACAACGCCCTGCAGATGCATTTGGCGAGTTTGAGCAGGACAGAAAGGCCGAGGAGGAGGGTGATCTTGAAGAGCAGCAACAACAACAATCTGGGAATATTGGTGGAGGAGCTTCTTTGGTGCCAAGGCAATTC |
| MN625732 | GgWRKY17 | AGGTGGAACCTTCACCAACAACAGGGTCCCTTCCTAGGCTTCAACAAACAGCACATGGTTCTGTGACTTCTGCTACATCTGCTGCATTTCCTGTAACCACTGTATGCTTCAATACCAATACTTTGGATGACGGAAAATCCAGCTTCTTTGAGTTCAAACCACACAGTAGATCAAATATGGTAATGAATTACTTGATGTTAGTAAAACTGATAAATATAAACTTTTGACTAAGGTGGAGACTTGGACTTTATTTTTTTCCTCCTTTAATTTATCTGATCGTAACCTGTGTACTAAGCTAACAAAATAGGATGATTAAGTAATATAATCTTTTCACTAGTTCCTTTATTTTTCCAGGGGGTTGTAAAGTGTTAGATGGAAACATATTTGATTGCCATGTATAACGTTCTGGTTAATATTGTTTTGGATATTACTGGTTGCATAGTACTTACAGAATGCTATTCACTTATTTAAGGTTCCTGCTGAATTAGACAACCGTGCATGTGAAAAATCTACTCAAATAGATGGTCAAGGAAAAGCTCAATCTTTTGCCTCATCACCATTAGTCAAAAGTGAAATTGCAGGCCCTTCAAATGAAATAAGTTTATCATCACCTGTTCAG |
| MN625733 | GgWRKY6 | TAAGAAAAGACTATGTCAAAAAAGGAATAGAATGTCATTTGATTAAACAAATAAAACCAGTAAGTATTTATTTATAATATAAAAAGGGGTATGTGACATTTATGCACATCTAGGAGGACGTAATTACAATTTCTCCTTGCACTTTTTTTTAACAGGGTAGGTTATTAAAGTCCCTGATGCAAGTTTTGCAGGCTTGCAGCGCATCTGTCTAGTCTTTCCAAGAAGTAAGTCCCTGACCTTCAGCAGAATCAGCACAAGCAGCTGATAGAACTTGTCATTAGAGTACATGTTCTTCCAAATAAAATGATGCCAATCAATTCTATATTCATTCCACTACTAGCAAACTGTTTCAGACAAGTTGAACAGGTGCACAAGCAAATACACACGAGCTGTTTCGCTAAAAGACTGACTACGCGCGAAGAAAAACAAGAAACAAGAAGAAAAGGATAAACATGGGAAGCTGTGAAATTACAAGATACCTGCCAATTAATTTATGTAGTTACAACTGCATGGTTTCTGAAAGATGAATTCAACAAGGCTTGATTTCAAAACCTATGCTCAGAAGCGTTCGAGCTGACTCTATCGCTTTCTCACCCCCAAGGTCCAAAGCTTCCCCAGTTAGTTCTCCTTCAGTGTCCACTGACCATTGAGTAGGAGTTTTCTGGTTTTGCGGCGAGTCGGGCTTCTTCACCTGCAAATTATTCATGGAGGCAGGTGCTGCAGCAGCCACAAGAGGAGCACTTGGTGGGCCATGACGTTTCTTGGGCACAGGCATGTCATGATCATGTACCCCCTTGTATGTTATGATGACAGCATCTGAGTTGTCTACAGCGGTCTCAATGTGCTTTCGGACAGGACATCCAGCTGAAGTGCATCTGTAGTAGTTTCTGAAAATTCAAGCAGTTCATCAGAAGAAAAATAAAAGATTTCAATTTCAATGGAAAATTGAAGAAAACAAAAAGGAAAGTCAACAGCCCATATAAAAGAGATAAGGTGGGTTTAAGGGTCAGTTTGGTCACAGTTCAATATAATTCATAAAGACAAAAAAACAGAAAAAACAGTTTGGCAAATTATTTGTTGTTTGGAGATTTTAAACCTAGTTTCCAGAAATCTTTTCTTAAATTAAGACGGGAAAAAAGAGAAAATTTGAAGCTATTTTTCTTTTTCAGTTTTTAAAAGAATTGATTTGTACTTTGGCATCATTGTAAATCATGGAAAACTTGTGATTTGGATTGTAAAGACATGTCTTCATCTTCTCTTCTCCAAGCGTGTTTCATTTTTCCTCTGCCTGCTGATCTGCTCTTCTTCCCAGGGTATGCTACCAAGCCCTCGATAGTTGGGATTTCTCCATGCCAGCAGTGACAGCTTTCCACACCACTGTCTTGGCCTGCCGTGGCAAGACCTCACCCTACATTCCATGGCCGGCTATCCAGCAGATGAGGAAGTGCCTCTCAATGGACGACCACCACAAACCATCTTCATTTAAGCCATCATGGACTCACTAAGAAAGTGGCATTGTCTAGCATAGTGATATCTACCGCTAAAAGCACTCAAAATCTTGTACTACGGCGCAGCCAAGTTAGGATGAAGATTTTAGGAGATGAACAGCAGAAAATTCATATAAGCACTTTTTTTAAAAAAGTGTTTAGGAGATGTTTATCCAAACAATTTACTGACTCTATTATTTCAACAGTTTATCATGAAACAAGTTGTGAAATTAAGGTTAAAATCAGACTTTAAAAACAGAAAATAGAAAAACAATTCAAAATAGGCCACAAATTGCTAGCAAATTTTACTTTAGAGAAATGCTGCTTCATCACTAAGATATCTGTCCTTACCCTTTTCCCCATAGGAAGCAGCTCTTTTCAGCAAAGCACAGAAGCAAAAGGACTACACCAACAACACCACAAATTTGACCTCAGAAGATAAGAAATCTTATCCTCCCTGTTGTGGAAGGGATAAACTGATTACTATTCCTACTGCAAATGATGCAAACACAAACTAAGCATGCAAAATATTTTGAGAGAATATTTGCATTAAGAGGGTTTTGGACAATAAAAATGCGGATATATGTCCCAGTTGCTGATATTGTTGATTTCTAGCACAGAAAAAAAAAAGCAAAAATGCATCCGCAAGCTTATATAAATAAGATGCCTCATTGTCTTTCTTCAAACTGCTTACAAGCAAATAAACGCAGCTTGCAACTTTTTACTGATTTTATATTTTGTTTTGTTTTTTAAATTTTAAACACTCATTGAACCCAAATACAACTCAACTTTCACCATCTTCAATTAAAGATACTGAT |
| MN714227 | GgWRKY20 | TGAAATTAACTTTCCGCAGTTACTTCTTTCTTTCAGGCAGTTTAGCAACCAATATGAACCGCCTCAAGGCTCCAACACACAGAATAGAAGAGCCCTTTTTACTCTCCTTAGATTAGTAGAACAACACCAATTCAATATCCTGTTCTTATATTTCCCCAAGACTCAGAATCAGTTTATCATTGAGAATAACGTCAGAGAATGAACCTAAATCACAGCACCAGCAAACACACACTCGAACTTATGGCATAACTATTGTTTCTATTTAAAAAGCAATAGTTCTGCAAAACATTTTCAATATTCAAATTAAGACAACTTTCAAAAAGACAACTTAAAAGCTTTAGTAGGCACAAAACAAGAATTACATGAGTCACAATATGTTGGAATTTAGATACATGTGTACCAAAATATACATCTTCACTTATTTATCCCTAATAATAATGATACCCAATGTAGTATTAATTGTGATTTGCATATTTAGTCAAACCGCCAATAGACAAGAAAAGAGTGAGCCAGAAGAAGGGAAAAGTAAAAGTTAGAATACAAATAGGAATCTTAATCCCACAACATATCTGTATATCCTATGTCATTGCCAACTTGTATGCTCTTCAGTAACTAGCTGCAGCTTCGTAAAACAATGCAAT |

**Table S7 Real-time primer sequences of the twenty six genes investigated in the present study**

| SNo. | Gene Name | Name | | Sequence (5’ to 3’) | Tm |
| --- | --- | --- | --- | --- | --- |
| 1 | Actin | RTActinF | | AGCCTGGATGGCAACATACATAGC | 50°C to 65°C |
|  |  | RTActinR | | ATGACATGGAGAAGATCTGGCATCA |  |
| 2 | GgWRKY2 | RTFGgWRKY2 | | TGCAATGTGCGCAAACATGTCGAGAGAG | 60°C |
|  |  | RTRGgWRKY2 | | TTCCAAAATCCATATCTTTAAGCAAAGG |  |
| 3 | GgWRKY4 | RTFGgWRKY4 | | GATGTTGCAAAACCCTTTGGACCCTCA | 58°C |
|  |  | RTRGgWRKY4 | | TACTGGTCCCATTAATTTCATTATTCAC |  |
| 4 | GgWRKY5 | RTFGgWRKY5 | | GATAACTACTAATTCGATGTCTAAGATG | 62°C |
|  |  | RTRGgWRKY5 | | GTGACAGTCCTATGTGAAGAAGCTGGAT |  |
| 5 | GgWRKY8 | RTFGgWRKY8 | | AGGAAATATGGACAGAAAGTAGTTAAAG | 62°C |
|  |  | RTRGgWRKY8 | | GTTGTTCAGAGAATTTCTGTTCATGTTA |  |
| 6 | GgWRKY14 | RTFGgWRKY14 | | GGCACTCCAGAGCTATCGCCTGTA | 60°C |
|  |  | RTRGgWRKY14 | | CCATTTTTCTGCTCAACAGAAAAC |  |
| 7 | GgWRKY15 | RTFGgWRKY15 | | AGGAGATGAAGATGCCCTTGGACCAGAA | 60°C |
|  |  | RTRGgWRKY15 | | TTACCTTGCATTTGGGTTTCCTTTAACT |  |
| 8 | GgWRKY20 | RTFGgWRKY20 | | TTGTGTGAGAGCAAGATGTGACACCCCA | 60°C |
|  |  | RTRGgWRKY20 | | GCAGTAGCTGACAGAGGAAGAGG |  |
| 9 | GgWRKY24 | RTFGgWRKY24 | | AGATGGAGACGGCGTCGTCCGCCATTCTCGA | 65°C |
|  |  | RTRGgWRKY24 | | CGTGAACCGCCGCCGGTCGTCCAGCA |  |
| 10 | GgWRKY29 | RTFGgWRKY29 | | GCACTACTACTTCAGCTGAAATAA | 52°C |
|  |  | RTRGgWRKY29 | | ATCATTATTAATGGAAGAGATCTGCATC |  |
| 11 | GgWRKY36 | RTFGgWRKY36 | | GTACCCAAGGAGCTATTACAGAT | 58°C |
|  |  | RTRGgWRKY36 | | GAACCTGCAAAAAGGGATGGTAT |  |
| 12 | GgWRKY38 | RTFGgWRKY38 | | TACCGGTGCACCATGGCTGCCGGT | 58°C |
|  |  | RTRGgWRKY38 | | AGTGTCCTTGTGAGGAAATTTGCA |  |
| 13 | GgWRKY40 | RTFGgWRKY40 | | ACAGTGTGGCTATGGCAGGCACATAA | 58°C |
|  |  | RTRGgWRKY40 | | ACTCTTCAGGAAGATCCAAATTGA |  |
| 14 | GgWRKY44 | RTFGgWRKY44 | | TTACGTCATGTGCCTACAAAGCTG | 60°C |
|  |  | RTRGgWRKY44 | | TGTTCCCATATTGCAACGATAGTA |  |
| 15 | GgWRKY45 | RTFGgWRKY45 | | TGACGATACCGATCGGTGACGTG | 60°C |
|  |  | RTRGgWRKY45 | | ACGCGGCTTCTCTCAACTTGCTTC |  |
| 16 | GgWRKY51 | RTFGgWRKY51 | | AAGTCTACCGTCTCCGATGAGAAA | 62°C |
|  |  | RTRGgWRKY51 | | ACCGTGCATTTGCTGTTTGTGCAC |  |
| 17 | GgWRKY53 | RTFGgWRKY53 | | TGAGAGCAGCAATAACAACAATAGCAAT | 55°C |
|  |  | RTRGgWRKY53 | | ATATGCTCTTGGACAAGGGTTATC |  |
| 18 | GgWRKY54 | RTFGgWRKY54 | | TATGTCAGGACCGAAGCATCTGATACCG | 60°C |
|  |  | RTRGgWRKY54 | | TGATTGTGCTCCCCTTCATAAGTA |  |
| 19 | GgWRKY55 | RTFGgWRKY55 | | CAATTACAGAGGAGGAATCATTA | 62°C |
|  |  | | RTRGgWRKY55 | TGCACCTTCTTTTTCACTGGGCAGCTTG |  |
| 20 | GgWRKY56 | | RTFGgWRKY56 | ATATCATCTCGCCTAAGGTTTACA | 60°C |
|  |  |  | RTRGgWRKY56 | TGCAACCAGTATAGTAGGGTCTTCTAC |  |
| 21 | GgWRKY57 | | RTFGgWRKY57 | TCACAGAGCTGGATTGGAAATCAAAACA | 58°C |
|  |  |  | RTRGgWRKY57 | AGAAAAACTTGCCTTCTCTTAGGGT |  |
| 22 | GgWRKY58 | | RTFGgWRKY58 | AGCATGTTGAAAGAGCCTCTCATGATT | 60°C |
|  |  |  | RTRGgWRKY58 | TGAGTCCAAACAGTTGCGTTCCCC |  |
| 23 | GgWRKY59 | | RTFGgWRKY59 | ATGCACTCACCCTAATTGTGAAGTGAA | 60°C |
|  |  |  | RTRGgWRKY59 | TCATCTGGCATTACCATCTCGGCT |  |
| 24 | GgWRKY62 | | RTFGgWRKY62 | TGAGTCCCATCTTCAAGAAATCAACAAG | 58°C |
|  |  |  | RTRGgWRKY62 | TTGATAGCCATCTTTTATTATCAAACTA |  |
| 25 | GgWRKY69 | | RTFGgWRKY69 | TCAGAATTCACAACAGCACAAGAGAAAA | 60°C |
|  |  |  | RTRGgWRKY69 | CTCTAAAAGCAAAGCTTGAACAT |  |
| 26 | GgWRKY70 | | RTFGgWRKY70 | TGAAATGTGGGAGCAGCAGTGCT | 65°C |
|  |  |  | RTRGgWRKY70 | GAAAGAGTGAAGAAATTTTCCAG |  |

FIGURE S2 Co-expression data of protein-protein associations

FIGURE S1 Conserved domain analysis of WRKY proteins identified from *Glycyrrhiza uralensis*
